# Causal relationship between human blood metabolites and risk of ischemic stroke: a Mendelian randomization study

**DOI:** 10.3389/fgene.2024.1333454

**Published:** 2024-01-19

**Authors:** Menghao He, Chun Xu, Renyi Yang, Lijuan Liu, Desheng Zhou, Siyang Yan

**Affiliations:** ^1^ The First Hospital of Hunan University of Chinese Medicine, Changsha, Hunan, China; ^2^ Hunan University of Chinese Medicine, Changsha, Hunan, China; ^3^ Changde College of Science and Technology, Changde, Hunan, China

**Keywords:** blood metabolites, ischemic stroke, Mendelian randomization, causal relationship, stroke

## Abstract

**Background:** Ischemic stroke (IS) is a major cause of death and disability worldwide. Previous studies have reported associations between metabolic disorders and IS. However, evidence regarding the causal relationship between blood metabolites and IS lacking.

**Methods:** A two-sample Mendelian randomization analysis (MR) was used to assess the causal relationship between 1,400 serum metabolites and IS. The inverse variance-weighted (IVW) method was employed to estimate the causal effect between exposure and outcome. Additionally, MR-Egger regression, weighted median, simple mode, and weighted mode approaches were employed as supplementary comprehensive evaluations of the causal effects between blood metabolites and IS. Tests for pleiotropy and heterogeneity were conducted.

**Results:** After rigorous selection, 23 known and 5 unknown metabolites were identified to be associated with IS. Among the 23 known metabolites, 13 showed significant causal effects with IS based on 2 MR methods, including 5-acetylamino-6-formylamino-3-methyluracil, 1-ribosyl-imidazoleacetate, Behenoylcarnitine (C22), N-acetyltyrosine, and N-acetylputrescine to (N (1) + N (8))-acetate,these five metabolites were positively associated with increased IS risk. Xanthurenate, Glycosyl-N-tricosanoyl-sphingadienine, Orotate, Bilirubin (E,E), Bilirubin degradation product, C_17_H_18_N_2_O, Bilirubin (Z,Z) to androsterone glucuronide, Bilirubin (Z,Z) to etiocholanolone glucuronide, Biliverdin, and Uridine to pseudouridine ratio were associated with decreased IS risk.

**Conclusion:** Among 1,400 blood metabolites, this study identified 23 known metabolites that are significantly associated with IS risk, with 13 being more prominent. The integration of genomics and metabolomics provides important insights for the screening and prevention of IS.

## Introduction

Stroke remains the second leading cause of death and the third leading cause of disability worldwide (GBD, 2019 Stroke Collaborators, 2021). Each year, approximately 795,000 individuals experience a new or recurrent stroke, with 87% of these being IS. IS occurs when a blockage in the brain’s arteries leads to ischemic damage or death of brain tissue, resulting in neurological dysfunction. Its high incidence, mortality, and disability rates are the main characteristics of this common clinical condition, placing a significant burden on individuals, families, and society (Tsao et al., 2023).

Metabolites are intermediate or end products of metabolic reactions, which not only influence the occurrence and development of diseases but also serve as targets for therapeutic interventions (Chen et al., 2023). Understanding the relationship between genetic variations and metabolites is of great significance in unraveling the biological mechanisms underlying human diseases (Gieger et al., 2008). With the advancement of high-throughput technologies, we are now able to measure hundreds of circulating metabolites and conduct concurrent genotyping in large population-based studies (Kettunen et al., 2016). In recent years, an increasing number of studies have identified metabolites from stroke patients and animal models that could improve the diagnosis and prediction of ischemic stroke outcomes (Zhang et al., 2022). For example, in a related study (Sun et al., 2019), two serum long-chain dicarboxylic acids (metabolites of fatty acid ω-oxidation) were associated with IS and cardiogenic embolic stroke, suggesting that pathways related to intracellular hexadecanedioic acid synthesis or its clearance from circulation may mediate the risk of ischemic stroke. However, the detailed pathophysiological mechanisms of ischemic stroke metabolites remain elusive. Therefore, a comprehensive and thorough analysis of the causal relationships of metabolites in ischemic stroke is urgently needed.

MR is a statistical method used to estimate causal effects of exposure factors on outcome variables, providing robust evidence for causal relationships between exposure and disease risk (Zuccolo and Holmes, 2017). When randomized controlled trials are lacking, causality is not feasible, or there are potential confounding factors or reverse causality, MR can serve as an effective alternative for assessing causal relationships (Davey Smith and Hemani, 2014).

Whether blood metabolites have a causal relationship with IS remains unclear. In this study, we systematically evaluate the potential causal relationships within a two-sample MR framework using Genome-wide association study (GWAS) summary data. We also conduct a series of sensitivity analyses and heterogeneity analyses to assess the causal effects of blood metabolites on the risk of IS, providing new insights for the prevention and treatment strategies of IS.

## Methods and materials

### Research Design

**FIGURE 1:**
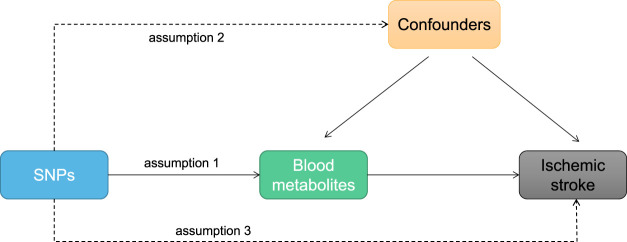
MR study design diagram: Hypothesis 1, genetic variation is closely associated with exposure; Hypothesis 2, genetic variation is independent of confounding factors; Hypothesis 3, genetic variation only affects outcome through exposure.

This study employed a two-sample MR design to systematically evaluate the causal relationship between 1,400 human blood metabolites and the risk of IS. Single nucleotide polymorphisms (SNPs) were used as instrumental variables to eliminate confounding factors. The MR design should adhere to the following three basic assumptions: 1) relevance assumption: a close association between SNPs and the exposure; 2) independence assumption: no association between SNPs and confounding factors; 3) exclusion restriction assumption: SNPs can only affect the outcome through the exposure factor. The second and third assumptions are collectively referred to as horizontal pleiotropy and independence, respectively, and can be tested using a series of statistical methods. (Refer to Figure 1 for further details).

### Data source

Previously, Shin et al. conducted a comprehensive exploration of the genetic impact on human metabolism through a GWAS of untargeted metabolomics, recruiting 7,824 participants from two European population cohorts. They successfully identified 486 metabolites with genetic effects on human serum metabolites. After rigorous quality control, a total of 486 metabolites were analyzed, including 309 known metabolites and 177 unknown metabolites (Shin et al., 2014). In the latest study (Chen et al., 2023), a GWAS was conducted on 1,091 blood metabolites and the ratios of 309 metabolites. The data can be obtained from the GWAS Catalog database (GWAS Catalog (ebi.ac.uk)). The catalog numbers for the 1,400 blood metabolites are GCST90199621-GCS90201020.

The data on IS are derived from a genome-wide association analysis published by Sakaue et al., in 2021, which includes 11,929 ischemic stroke patients, 472,192 healthy individuals, and more than 24.17 million SNPs. The original study obtained informed consent from all participants (Sakaue et al., 2021). These data can be accessed on the website: https://gwas.mrcieu.ac.uk. (GWAS ID: ebi-a-GCST90018864).

### Instrumental variable selection

A unified selection criterion was used for the genetic variations of the 1,400 metabolites. First, considering that the number of SNPs for achieving full genome-wide significance metabolites may be limited, we relaxed the threshold by setting *p*-value to be less than 1 × 10^−5^ (Yang et al., 2020). However, since this sample size was large and we obtained numerous results, we further increased the *p*-value and ultimately selected a stricter threshold in the MR analysis. The selection criteria for strong positive exposure results were that *p* < 1 × 10^−8^ was significant for association analysis results (Wang et al., 2023). After extracting the significant SNPs corresponding to each metabolite, linkage disequilibrium was analyzed, which was considered to exist if the linkage disequilibrium parameter r^2 was <0.1 and the distance between SNPs was within 500 kb (Yang et al., 2020). In addition, to eliminate bias caused by weak instrumentality, the *F*-value of each SNP was calculated, and SNPs with an *F*-value < 10 were considered weak instruments (Choi et al., 2019).

### MR analysis

The IVW method was used as the primary MR method to estimate the causal effect relationship between metabolites and IS. This method calculates weighted estimates by taking the inverse of the variances under the assumption that all instrumental variables are valid. IVW is the main method in MR studies, which combines the Wald ratio of each SNP to obtain a summary estimate (Pierce and Burgess, 2013). However, there may still be unknown confounding factors causing genetic pleiotropy and bias in the estimation of effect sizes. Therefore, MR-Egger regression and weighted median methods were used as complementary methods to validate the causal effect of exposure on the outcome. The “leave-one-out” method was used to detect any instrumental variables that may affect the estimation of causal effects. This involves gradually removing individual single SNP and calculating the Meta effect of the remaining SNPs. We observe whether the results change after removing each SNP to determine if the results are influenced by any specific SNP (Feng et al., 2022; Zhang et al., 2023). Horizontal pleiotropy was evaluated based on the Egger intercept, and if *p* > 0.05, it was considered to have no horizontal pleiotropy (Cai et al., 2022). In addition, heterogeneity tests were performed on the instrumental variables obtained, and if the test result had *p* > 0.05, the influence of heterogeneity could be ignored.

## Results

**FIGURE 2:**
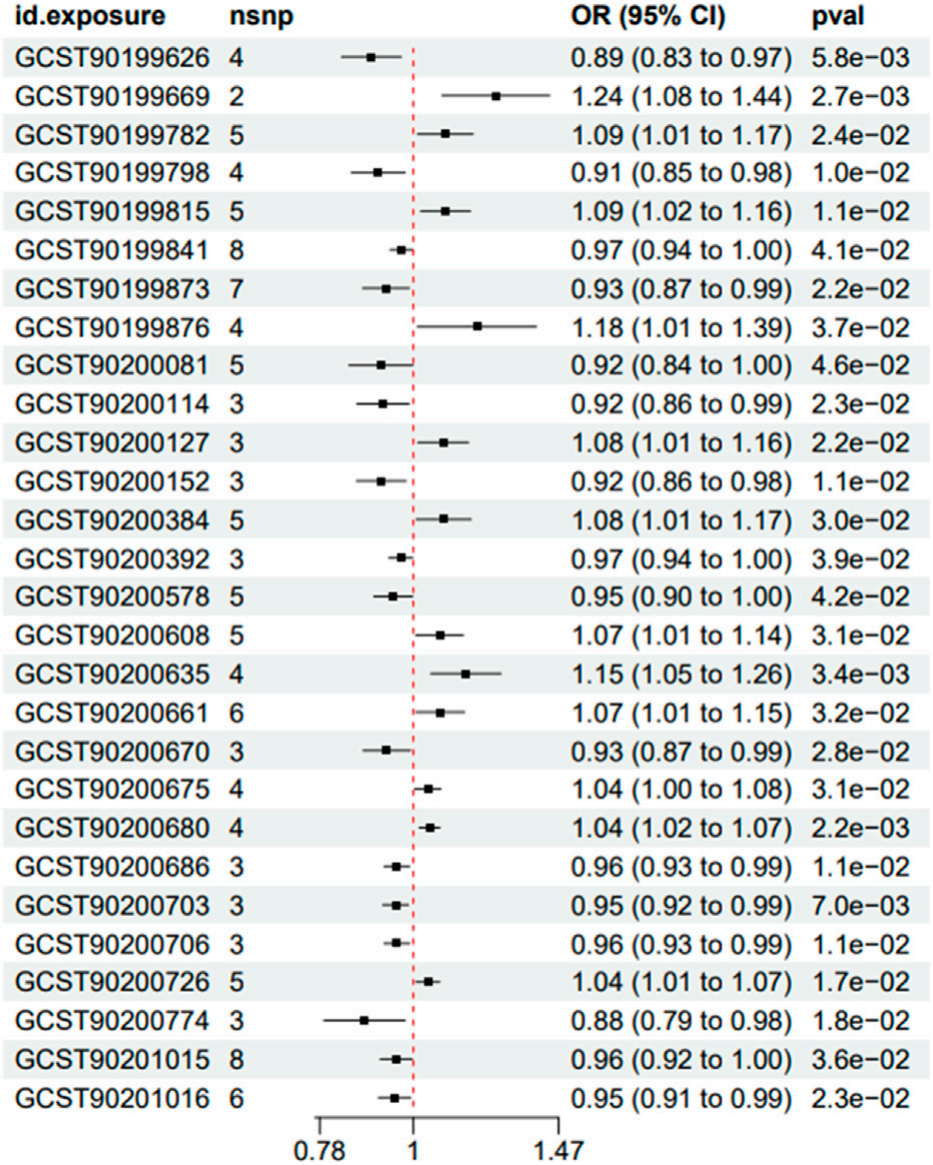
Forest plots showed the causal associations between serum metabolites and IS; The analysis method is IVW.

**FIGURE 3:**
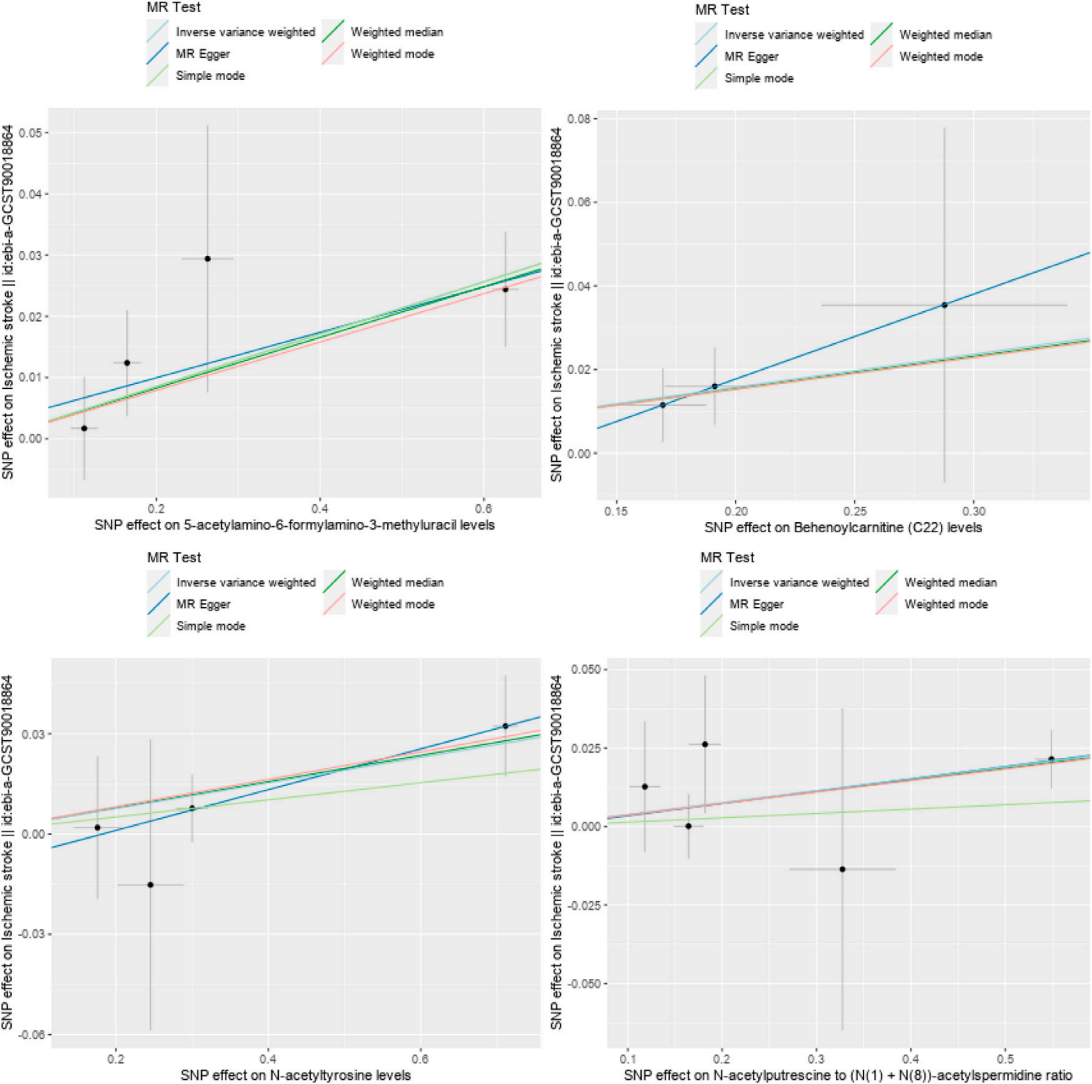
Scatter plots of four metabolites related to reducing the risk of IS.

A total of 28 metabolites related to IS were preliminarily detected using the IVW method, including 23 known metabolites and 5 unknown metabolites. Among the 23 known metabolites, 9 were potentially associated with an increased risk of IS, namely,: Acetylcarnitine levels (Biocrates platform), Glutamine degradant, 1-ribosyl-imidazoleacetate, Palmitoyl sphingomyelin (d18:1/16:0), Behenoylcarnitine (C22), Guanidinoacetate, N-acetyltyrosine, 5-acetylamino-6-formylamino-3-methyluracil, and N-acetylputrescine to (N (1) + N (8))-acetylspermidine ratio. Additionally, 14 metabolites were potentially associated with a decreased risk of ischemic stroke, namely,: Xanthurenate, Isobutyrylglycine, Glycocholenate sulfate, Pregnenolone sulfate, 1-oleoyl-2-docosahexaenoyl-GPC (18:1/22:6), Glycosyl-N-tricosanoyl-sphingadienine (d18:2/23:0), 2-butenoylglycine, Orotate, Bilirubin (E,E), Bilirubin degradation product, C_17_H_18_N_2_O_4_ (3), Biliverdin, Uridine to pseudouridine ratio, Bilirubin (Z,Z) to androsterone glucuronide ratio, and Bilirubin (Z,Z) to etiocholanolone glucuronide ratio. Refer to Figures 2, 3 for further details.

**FIGURE 4:**
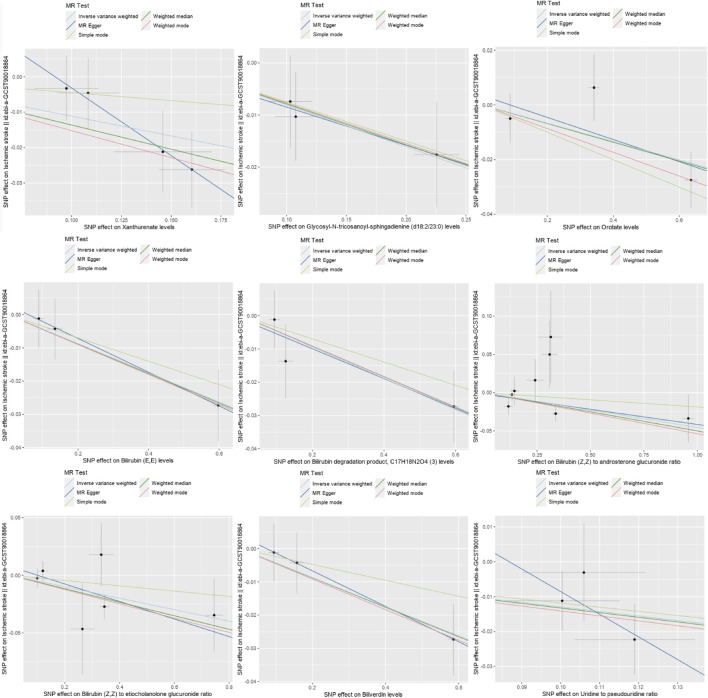
Scatter plots of nine metabolites related to increasing the risk of IS.

In order to eliminate the influence of other confounding factors on the results, the causality between blood metabolites and IS was evaluated using a combination of MR-Egger regression, Weighted median, Simple mode, and Weighted mode methods on top of the IVW analysis. Multiple-effect and heterogeneity tests were conducted as well. The results revealed that among the 23 known metabolites, 13 showed *p*-values smaller than 0.05 in 2 MR models (Figures 3, 4 for further details). Unfortunately, X-25422, which belongs to the unknown metabolites, had 3 MR models with *p*-values smaller than 0.05.

**FIGURE 5:**
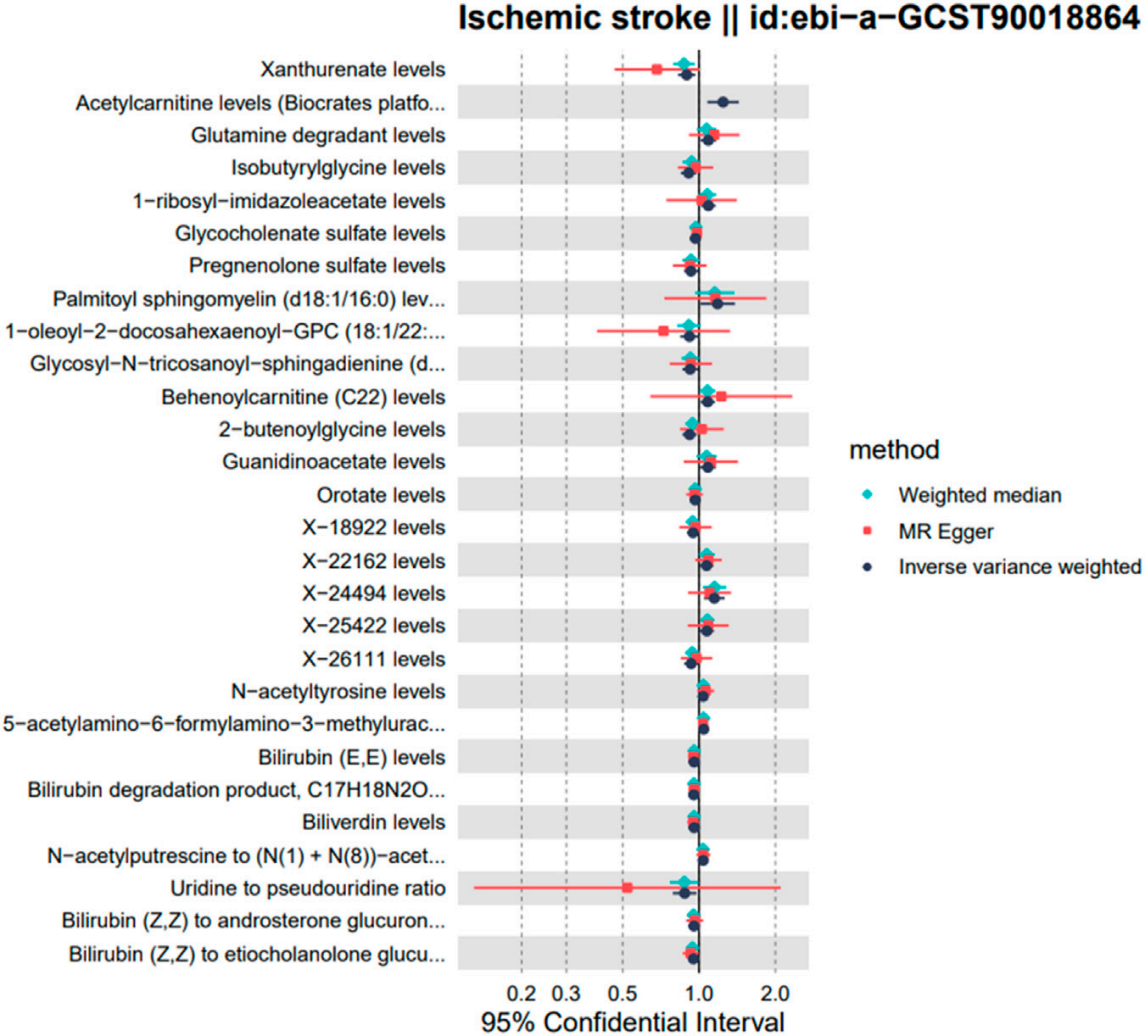
Forest plots showed the causal associations between serum metabolites and IS; The analysis method is IVW, weighted median and MR egger.

**FIGURE 6:**
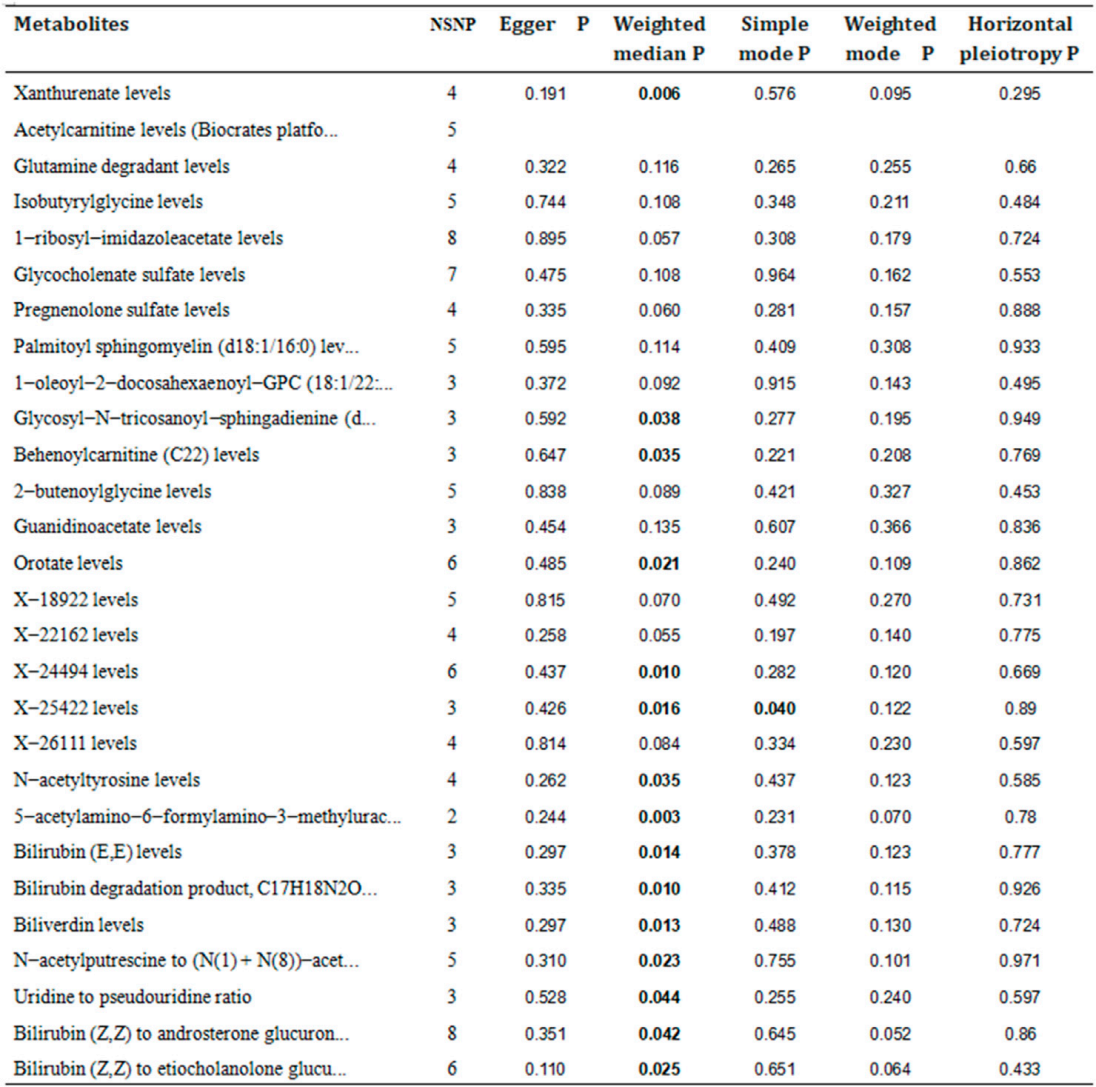
*p*-values for 4 MR models (MR egger, weighted median, simple mode, weighted mode) and horizontal pleiotropy.

Except for 2-butenoylglycine, which showed a different direction of causality in the MR-Egger regression compared to the IVW method, the other metabolites showed consistent directions of causality across all four analysis methods. Horizontal pleiotropy analysis based on MR-Egger intercept showed that all 28 metabolites had *p*-values greater than 0.05, indicating a low risk of horizontal pleiotropy (Refer to Figures 5, 6 for further details).

**FIGURE 7:**
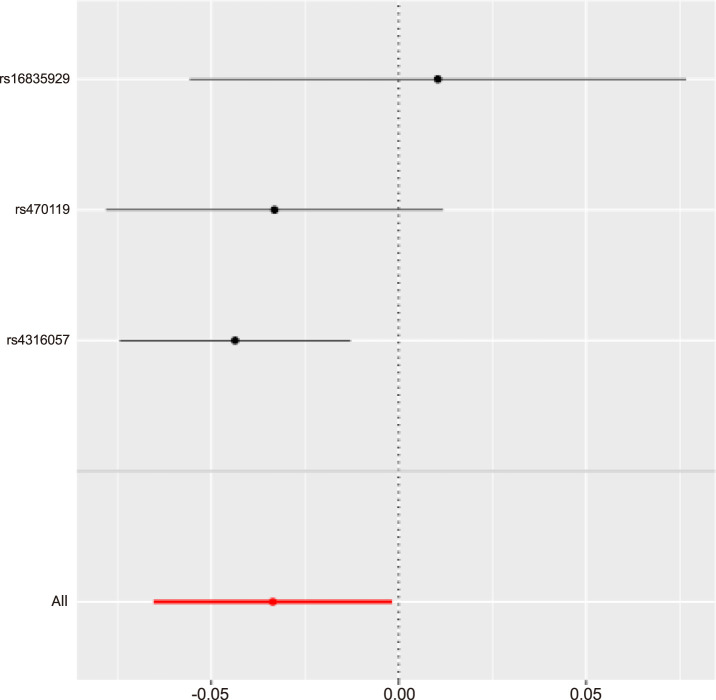
MR leave-one-out sensitivity analysis for Orotate levels on IS|||Id:ebi-a-GCST90018864.

Sensitivity analysis using leave-one-out method revealed that the SNP rs4316067 had a strong influence on the causality between Orotate and ischemic stroke (Refer to Figure 7 for further details). Additionally, due to insufficient instrumental variables, the value of Acetylcarnitine levels (Biocrates platform) could not be detected by the other four methods apart from IVW.

## Discussion

Our results confirm the causal relationship between 28 metabolites and IS. Among these 28 metabolites, 5 are unknown metabolites, while 13 known metabolites show potential causal relationships with IS.

This study utilized available GWAS data and two-sample MR methods to explore the causal relationship between IS and blood metabolites. Extensive sensitivity analyses were conducted to control for confounding factors and enhance the reliability of the results. This study could be one of the first to combine metabolomics and genomics to investigate the causal relationship between serum metabolites and IS.

Among the metabolites that contribute to increased risk of IS, 5-acetylamino-6-formylamino-3-methyluracil is a caffeine metabolite (Birch Kristensen et al., 2018). However, caffeine has neuroprotective effects and can also regulate stroke risk factors such as blood glucose and lipid levels (Fan et al., 2023). This finding seems contradictory to our study results. In a study (Zheng et al., 2022), high caffeine intake was associated with an increased risk of cardiovascular diseases. Therefore, different caffeine contents may lead to different outcomes in relation to disease. Behenoylcarnitine (C22) is a type of acylcarnitine, which is an important lipid carrier that reflects the mitochondrial β-oxidation status. It plays a crucial role in fatty acid β-oxidation, and elevated levels of acylcarnitines indicate β-oxidation dysfunction (Xu et al., 2016). Furthermore, studies have shown a potential association between acylcarnitines and an increased risk of stroke (Seo et al., 2018; Huang et al., 2023), but research specifically on Behenoylcarnitine (C22) and its implications is limited. Similarly, there is limited research on N-acetylputrescine, acetylspermidine, and ischemic stroke. However, a metabolomics study has suggested that N-acetylputrescine may serve as a diagnostic biomarker for Parkinson’s disease (LeWitt et al., 2023), which is a potential cause of cardioembolic and large artery strokes (Fang et al., 2022). Additionally, research has shown that N8-acetylspermidine is a novel biomarker in ischemic cardiomyopathy, and higher levels of N8-acetylspermidine are associated with increased mortality in patients with ischemic cardiomyopathy (Nayak et al., 2020). Therefore, investigating the association between N-acetylputrescine, acetylspermidine, and stroke may provide a new research direction. N-acetyltyrosine is an endogenous trigger factor in mammalian and insect mitochondria. Currently, there are no reports of the presence of N-acetyltyrosine in the blood of healthy individuals, but it has been found in human serum (Matsumura et al., 2020). Studies have found that pre-treatment with N-acetyltyrosine can reduce serum levels of peroxidized lipids. Additionally, as an effective mitochondrial inducer, N-acetyltyrosine can enhance stress resistance in insects and mice (Matsumura et al., 2020; Matsumura et al., 2023). This also offers a new avenue of research for us.

Among the metabolites associated with reducing the risk of IS, Xanthurenate is a metabolite of tryptophan metabolism, where over 95% of tryptophan in dogs is metabolized to kynurenine, and Xanthurenate is one of the intermediate products (Ngui et al., 2020). Kynurenate is an effective endogenous N-Methyl-D-aspartic acid (NMDA) glutamate receptor antagonist, making it a potential therapeutic tool for reducing excitotoxicity and neuroinflammation (Hertelendy et al., 2018). Additionally, kynurenate appears to have a neuroprotective effect in hippocampal ischemia-induced injury (Sas et al., 2008). Glycosyl-N-tricosanoyl-sphingadienine is one of the metabolites of sphingolipids, which are considered central hub of sphingolipid metabolism and can influence critical metabolic functions. However, high levels of sphingolipids have been observed in the serum of stroke patients with poor prognosis. Furthermore, sphingolipids are considered pro-apoptotic molecules, and elevated levels of long-chain sphingolipids are associated with blood-brain barrier disruption (Ouro et al., 2022). This contradicts our research findings. Orotate is a precursor of pyrimidine biosynthesis and can be absorbed by red blood cells and hepatocytes, converted to uridine, and used in the pyrimidine salvage pathway (Löffler et al., 2015). Previous studies have suggested potential therapeutic effects of orotate in hypertension-related complications and myocardial infarction (Choi et al., 2006; McCarty and DiNicolantonio, 2014). However, there is limited research on orotate in the context of stroke disease. Bilirubin (E,E), Bilirubin degradation product, Bilirubin (Z,Z) are isomers or metabolites of bilirubin. Although there is limited research on these substances, a significant body of research has shown a close relationship between bilirubin metabolism and stroke. A previous Mendelian randomization study found a significant causal relationship between high levels of bilirubin and reduced stroke risk in the Korean population (Choi et al., 2020). However, recent research has found a negative correlation between serum bilirubin elevation and stroke prognosis. After stroke occurrence, ischemic injury induces the release of endogenous bilirubin, which increases neuronal excitability and ischemic infarct volume through the regulation of the TRPM2 pathway (Liu et al., 2023). Despite the growing evidence of the anti-inflammatory and neuroprotective effects of bilirubin, there is still controversy regarding the association between serum bilirubin levels and the occurrence and prognosis of stroke. This may be due to the different mechanisms by which serum bilirubin levels affect stroke before and after its onset (Wang et al., 2020). Androsterone glucuronide and etiocholanolone glucuronide are metabolites of human steroid hormones (Wang et al., 2021). Although research has shown an association between neurosteroid glucuronides and acute ischemic stroke, the specific mechanisms are not yet clear. Therefore, the Bilirubin (Z,Z) to androsterone glucuronide ratio and Bilirubin (Z,Z) to etiocholanolone glucuronide ratio are new research directions for understanding the mechanisms of IS. Biliverdin is a product of heme metabolism and has significant anti-inflammatory and antioxidant effects. Research has shown that biliverdin inhibits the NF-κB pathway through the Nrf2/A20/eEF1A2 axis and suppresses cell apoptosis to alleviate brain ischemia-reperfusion injury (Bai et al., 2023). Additionally, biliverdin reductase, also known as biliverdin, converts biliverdin to bilirubin. The biliverdin-bilirubin oxidative-reductive system formed during this conversion process can consume a large amount of oxygen free radicals (McDonagh, 2010). This may be a potential reason for its ability to reduce the risk of stroke. Uridine is a pyrimidine nucleoside that plays a crucial role in RNA synthesis and metabolism (Yamamoto et al., 2011). Pseudouridine is an isomer of uridine and the most abundant post-transcriptional RNA modification. In an observational study of a stroke case cohort, pseudouridine was associated with IS, while uridine was negatively correlated with stroke risk (Ament et al., 2022). The opposite direction of the association between uridine and pseudouridine seems to suggest the role of the Uridine to pseudouridine ratio in stroke risk. Furthermore, plasma uridine plays a critical role in energy homeostasis and thermoregulation. It regulates leptin signaling and may influence glucose and insulin metabolism (Deng et al., 2017). Pseudouridine and uridine have also been identified as related to the incidence of atrial fibrillation (Alonso et al., 2019). Given that diabetes and atrial fibrillation can also trigger ischemic stroke, further research on the association of the Uridine to pseudouridine ratio with IS warranted.

Previous research on metabolomic characteristics of acute IS thrombi has shown that diacylglycerol, phytosphingosine, galabiosylceramide, glucosylceramide, and 4-hydroxynonenal are related to thrombus formation. Metabolite enrichment analysis revealed a correlation between the glycolytic phenotype and thrombus formation (Arul et al., 2023). In our study, we found that Glycosyl-N-tricosanoyl-sphingadienine, as a metabolite of ceramide, has a potential causal relationship with IS. Additionally, we discovered that the Uridine to pseudouridine ratio is associated with IS, and plasma uridine is related to glucose and insulin metabolism. Furthermore, a metabolomic study identified 21 different metabolites (such as 4-hydroxyphenylpyruvic acid, cafestol, and (O-acyI)-1-hydroxy fatty acid (36:3)) between hypertensive IS patients and healthy individuals, some of which are acetyl-related (Zhao et al., 2023). These findings are consistent with our results; however, our study showed significant differences, possibly due to factors such as ethnicity, sample size, and the complexity of the diseases (hypertension and IS). Metabolomic characteristics play a crucial role in the management of various diseases, and increasing research has identified metabolites from ischemic stroke patients and animal models, which can improve the diagnosis and prediction of IS outcomes.

In conclusion, our research findings are partially consistent with previous studies. Based on existing literature, there is a significant association between serum metabolites and the risk and development of IS. Our study identified four acetyl-related metabolites that are associated with an increased risk of IS, suggesting that acetylation modifications may play an important role in the progression of IS. On the other hand, we found four metabolites related to bilirubin metabolism that are associated with a decreased risk of IS, indicating that bilirubin may also play a role in reducing IS risk. However, existing studies have shown different effects of bilirubin before and after stroke occurrence, which is important for screening before stroke, treatment, and prognosis. Neuroamide and its metabolites have also been found to play an important role in stroke, along with several metabolites involved in amino acid and glucose metabolism pathways. Given the complexity of the relationship between IS and metabolites, further investigation of protein acetylation modifications, heme metabolism, and neuroamide metabolic pathways may provide new directions for the diagnosis and clinical management of IS. While some studies have reported on other metabolites, the research in this area is still very limited.

The main strength of this study is the inclusion of a wide range of blood metabolites, in short, a total of 1,400 metabolites were included for MR analysis, making it the most comprehensive study to investigate the association between blood metabolites and IS. Furthermore, the MR design was applied to eliminate the influence of confounding factors and reverse causality. However, our study has some limitations. Firstly, both GWAS datasets used in our study were from European populations. It would be worthwhile to investigate whether the same results apply to other ethnic groups, which should be explored in future research. Secondly, we utilized summary-level GWAS data from large sample sizes, but IS also categorized into several subtypes. It is important to further investigate whether different subtypes of IS have different metabolite associations. Additionally, due to the unique nature of the blood-brain barrier, metabolites in the nervous system may have differences compared to blood metabolites. Moreover, the specific metabolic pathways of the identified metabolites with causal relationships in this study are important areas for future research. Thirdly, when selecting instrumental variables, we set a strict threshold of 1 × 10^−8^ for *p*-values due to the large number of metabolites available. However, this resulted in a smaller number of SNPs for individual metabolites, and despite conducting multiple sensitivity analyses, there may still be confounders affecting their pleiotropy (such as common genetic variations that may affect multiple traits). Lastly, although MR analysis provides valuable insights into metabolites related to IS, it should be noted that our study findings should be validated through rigorous randomized controlled trials, basic research, and future replication experiments using larger GWAS datasets of IS and metabolites.

In summary, this MR study identifies 23 known blood metabolites that are associated with the risk of IS, with 13 metabolites showing potential causal associations with IS risk. This provides preliminary evidence for the impact of blood metabolite dysregulation on the risk of IS.

## Data Availability

The original contributions presented in the study are included in the article/Supplementary Material, further inquiries can be directed to the corresponding authors.

## Abbreviations

GWAS, Genome-wide association study; IS, Ischemic stroke; IVW, inverse variance-weighted; MR, Mendelian randomization; SNP, Single nucleotide polymorphism.
