# Supplementary material for: Causal relationship between human blood metabolites and risk of ischemic stroke: a Mendelian randomization study
Source: Front Genet. 2024 Jan 19;15:1333454. doi: 10.3389/fgene.2024.1333454 (PMC10834680; doi:10.3389/fgene.2024.1333454)
Supplement: Supplementary file 1 [file DataSheet1.ZIP › serum_metabolites/GCST90199798_leaveOneOut_plot.pdf]

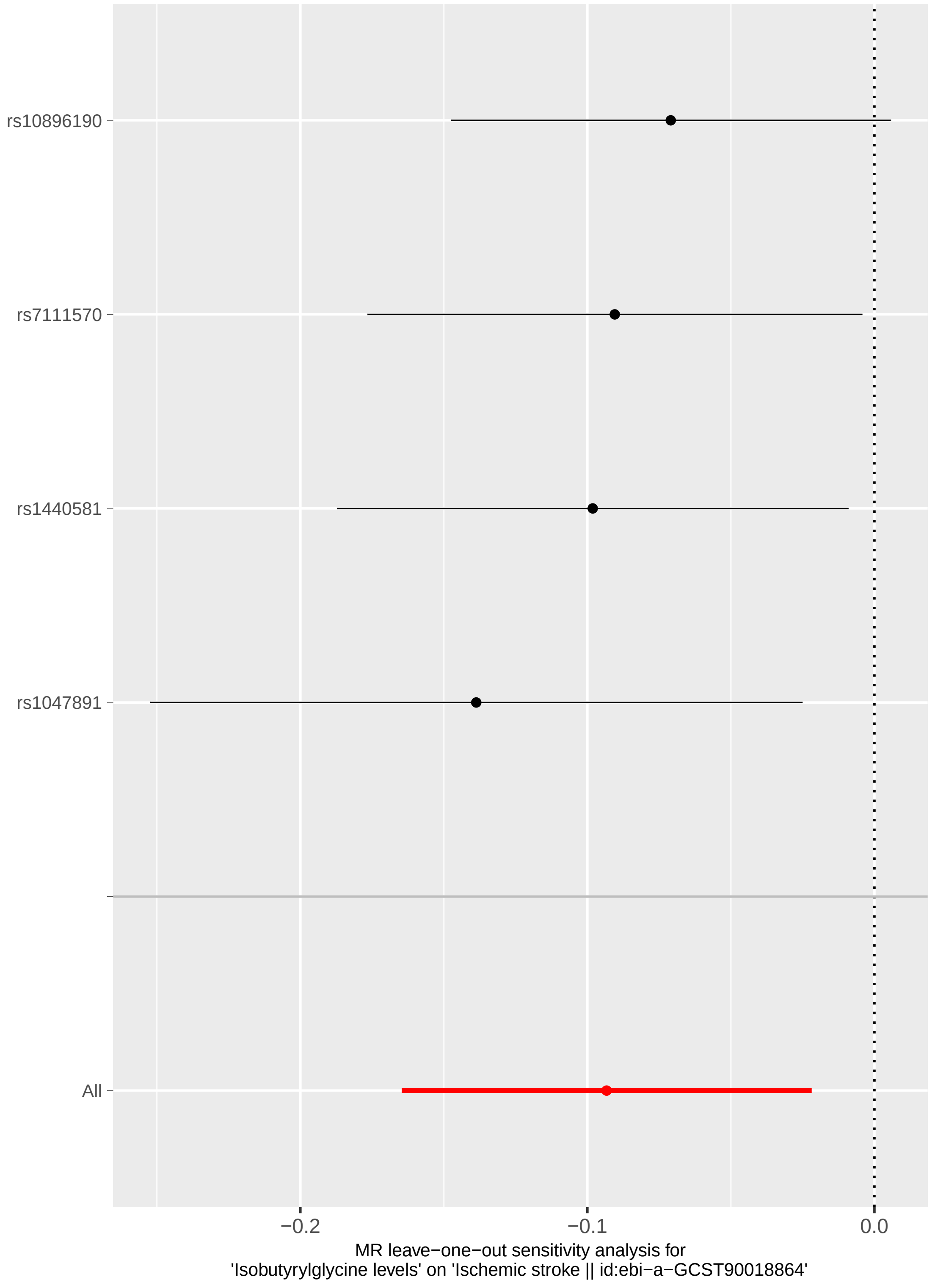
rs10896190
rs7111570
rs1440581
rs1047891
All
−0.2
−0.1
0.0
MR leave−one−out sensitivity analysis for
'Isobutyrylglycine levels' on 'Ischemic stroke || id:ebi−a−GCST90018864'
