# Supplementary material for: Causal relationship between human blood metabolites and risk of ischemic stroke: a Mendelian randomization study
Source: Front Genet. 2024 Jan 19;15:1333454. doi: 10.3389/fgene.2024.1333454 (PMC10834680; doi:10.3389/fgene.2024.1333454)
Supplement: Supplementary file 1 [file DataSheet1.ZIP › serum_metabolites/GCST90199841_leaveOneOut_plot.pdf]

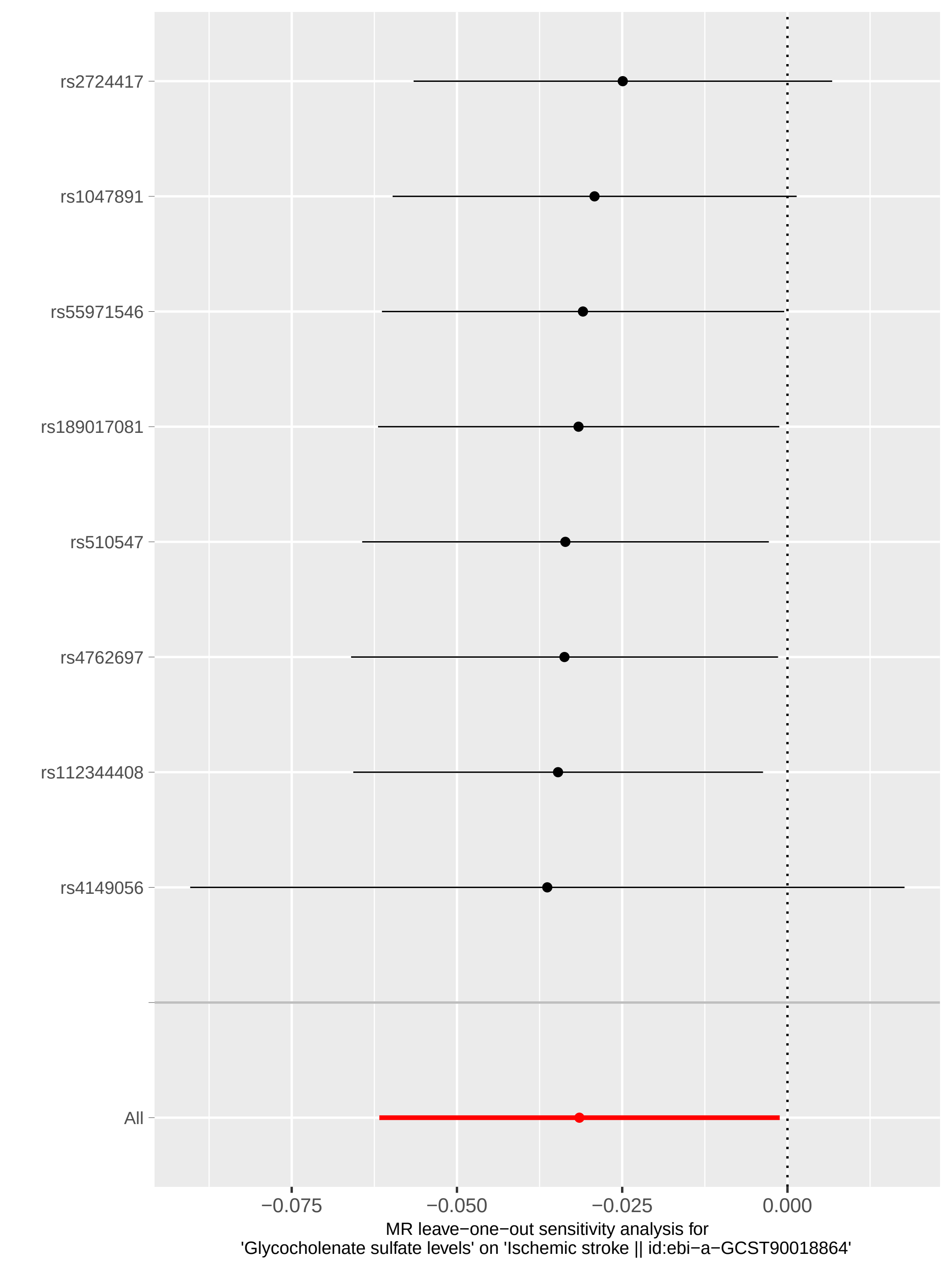
rs2724417
rs1047891
rs55971546
rs189017081
rs510547
rs4762697
rs112344408
rs4149056
All
−0.075
−0.050
−0.025
0.000
MR leave−one−out sensitivity analysis for
'Glycocholenate sulfate levels' on 'Ischemic stroke || id:ebi−a−GCST90018864'
