# Supplementary material for: Causal relationship between human blood metabolites and risk of ischemic stroke: a Mendelian randomization study
Source: Front Genet. 2024 Jan 19;15:1333454. doi: 10.3389/fgene.2024.1333454 (PMC10834680; doi:10.3389/fgene.2024.1333454)
Supplement: Supplementary file 1 [file DataSheet1.ZIP › serum_metabolites/GCST90199876_leaveOneOut_plot.pdf]

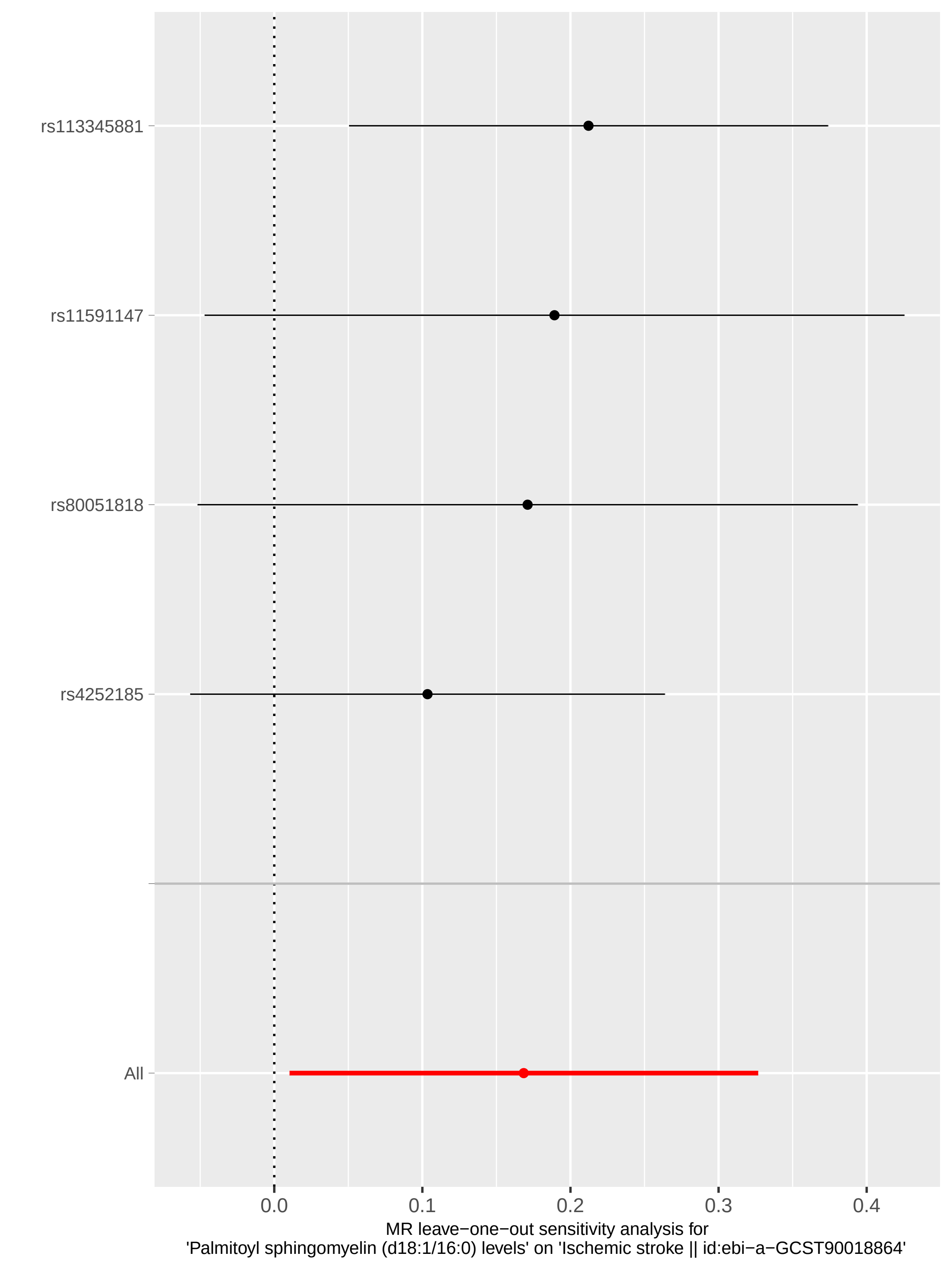

rs113345881
rs11591147
rs80051818
rs4252185
All
0.0
0.1
0.2
0.3
0.4
MR leave−one−out sensitivity analysis for
'Palmitoyl sphingomyelin (d18:1/16:0) levels' on 'Ischemic stroke || id:ebi−a−GCST90018864'
