# Supplementary material for: Causal relationship between human blood metabolites and risk of ischemic stroke: a Mendelian randomization study
Source: Front Genet. 2024 Jan 19;15:1333454. doi: 10.3389/fgene.2024.1333454 (PMC10834680; doi:10.3389/fgene.2024.1333454)
Supplement: Supplementary file 1 [file DataSheet1.ZIP › serum_metabolites/GCST90200081_leaveOneOut_plot.pdf]

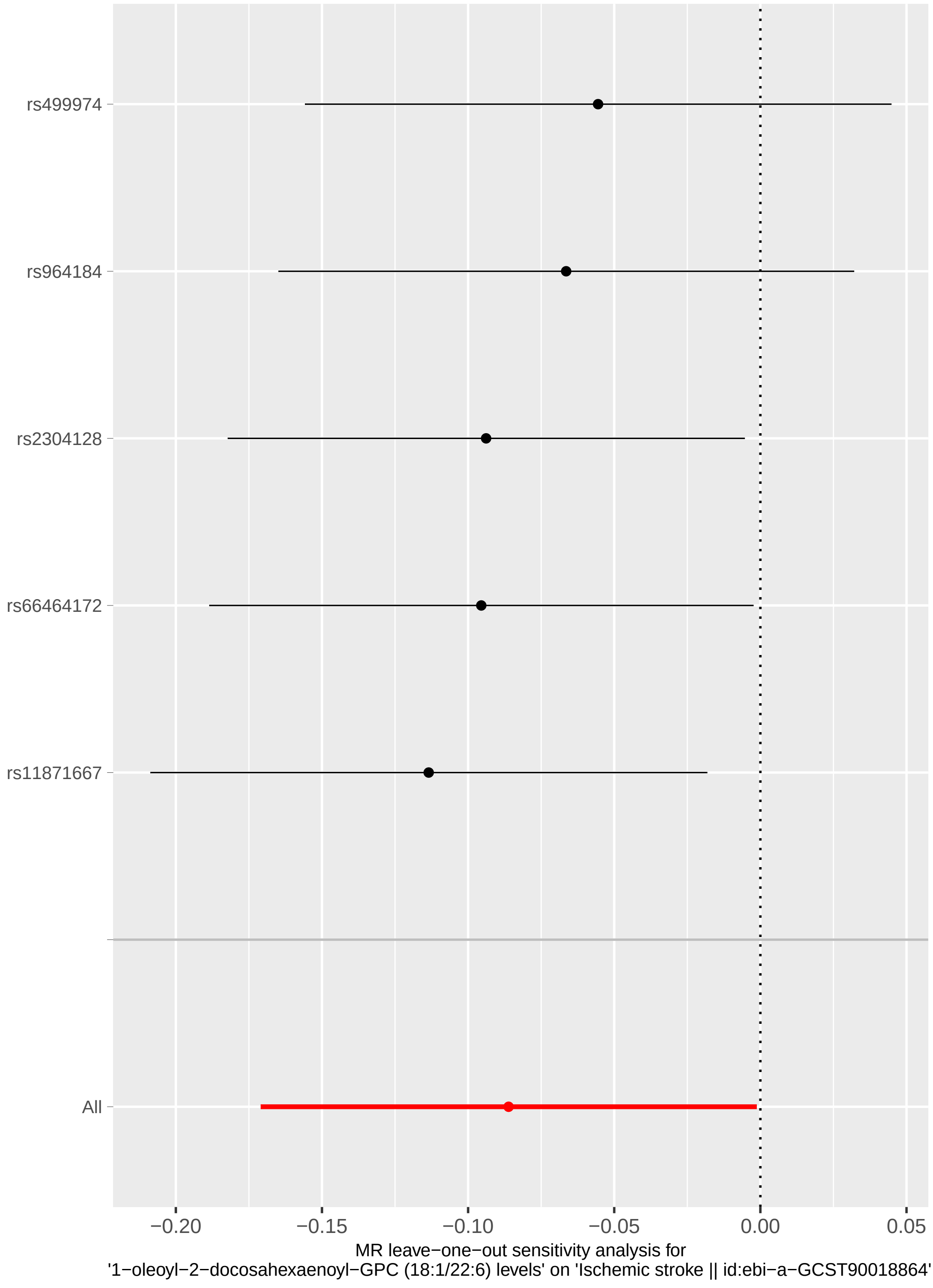
rs499974
rs964184
rs2304128
rs66464172
rs11871667
All
−0.20
−0.15
−0.10
−0.05
0.00
0.05
MR leave−one−out sensitivity analysis for
'1−oleoyl−2−docosahexaenoyl−GPC (18:1/22:6) levels' on 'Ischemic stroke || id:ebi−a−GCST90018864'
