# Supplementary material for: Causal relationship between human blood metabolites and risk of ischemic stroke: a Mendelian randomization study
Source: Front Genet. 2024 Jan 19;15:1333454. doi: 10.3389/fgene.2024.1333454 (PMC10834680; doi:10.3389/fgene.2024.1333454)
Supplement: Supplementary file 1 [file DataSheet1.ZIP › serum_metabolites/GCST90200675_scatter_plot.pdf]

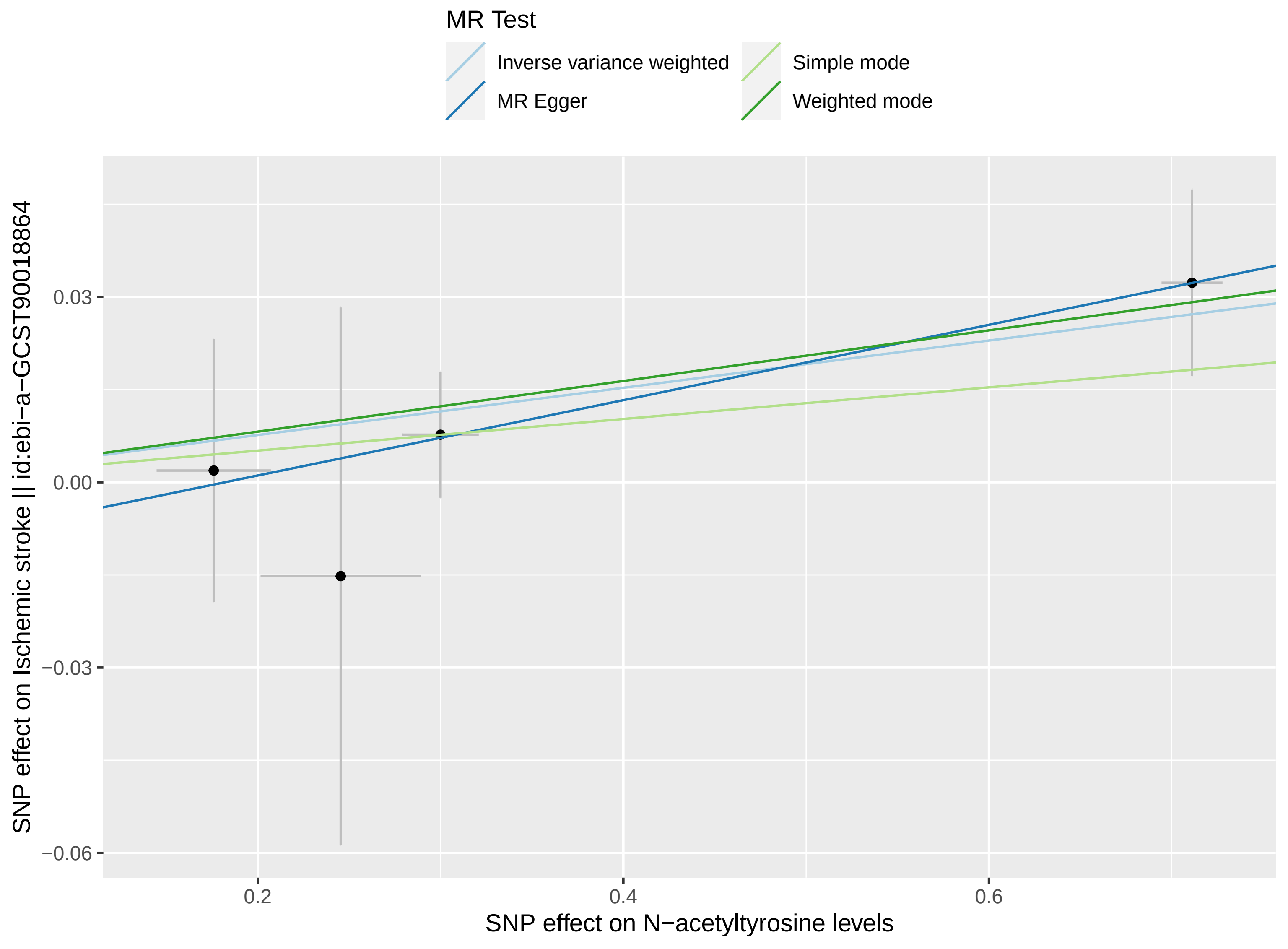
MR Test
Inverse variance weighted
MR Egger
Simple mode
Weighted mode
0.03
0.00
−0.03
−0.06
0.2
0.4
0.6
SNP effect on N−acetyltyrosine levels
SNP effect on Ischemic stroke || id:ebi−a−GCST90018864
