# Supplementary figures and images for: Causal relationship between human blood metabolites and risk of ischemic stroke: a Mendelian randomization study (part 2 of 2)
Source: Front Genet. 2024 Jan 19;15:1333454. doi: 10.3389/fgene.2024.1333454 (PMC10834680; doi:10.3389/fgene.2024.1333454)

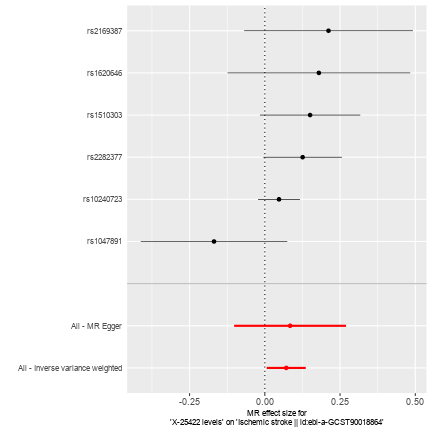

Supplement: Supplementary file 1 [file DataSheet1.ZIP › serum_metabolites/figure/X25422_levels_against_Ischemic_stroke__idebiaGCST90018864chunk5-1.png]

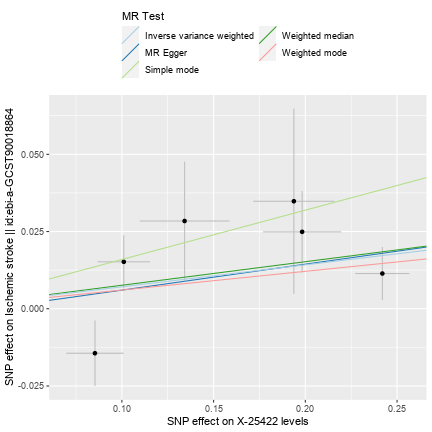

Supplement: Supplementary file 1 [file DataSheet1.ZIP › serum_metabolites/figure/X25422_levels_against_Ischemic_stroke__idebiaGCST90018864chunk6-1.png]

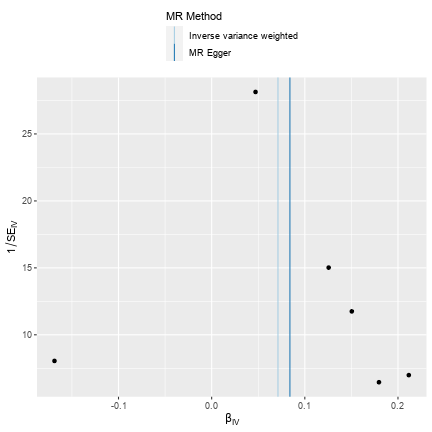

Supplement: Supplementary file 1 [file DataSheet1.ZIP › serum_metabolites/figure/X25422_levels_against_Ischemic_stroke__idebiaGCST90018864chunk7-1.png]

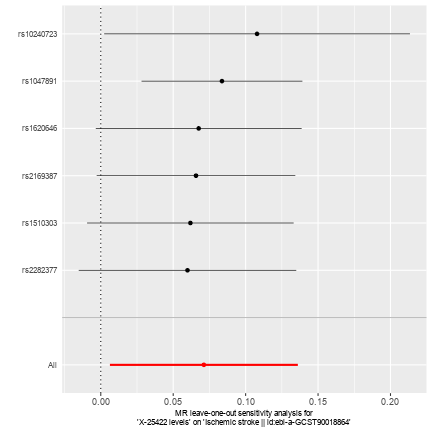

Supplement: Supplementary file 1 [file DataSheet1.ZIP › serum_metabolites/figure/X25422_levels_against_Ischemic_stroke__idebiaGCST90018864chunk8-1.png]

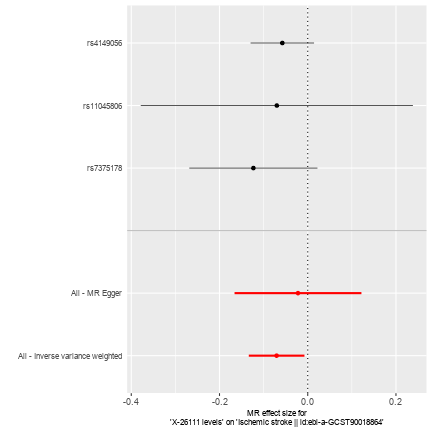

Supplement: Supplementary file 1 [file DataSheet1.ZIP › serum_metabolites/figure/X26111_levels_against_Ischemic_stroke__idebiaGCST90018864chunk5-1.png]

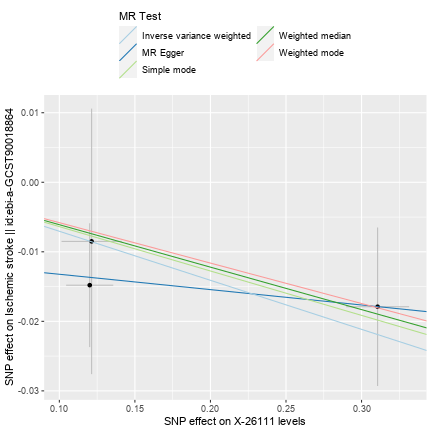

Supplement: Supplementary file 1 [file DataSheet1.ZIP › serum_metabolites/figure/X26111_levels_against_Ischemic_stroke__idebiaGCST90018864chunk6-1.png]

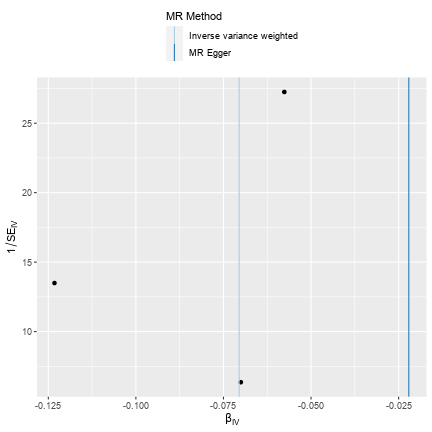

Supplement: Supplementary file 1 [file DataSheet1.ZIP › serum_metabolites/figure/X26111_levels_against_Ischemic_stroke__idebiaGCST90018864chunk7-1.png]

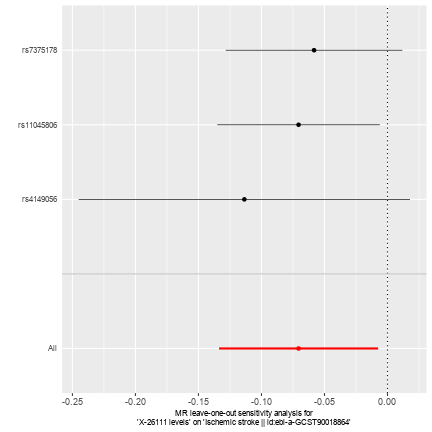

Supplement: Supplementary file 1 [file DataSheet1.ZIP › serum_metabolites/figure/X26111_levels_against_Ischemic_stroke__idebiaGCST90018864chunk8-1.png]

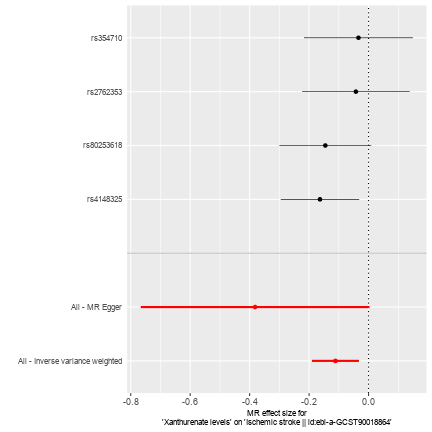

Supplement: Supplementary file 1 [file DataSheet1.ZIP › serum_metabolites/figure/Xanthurenate_levels_against_Ischemic_stroke__idebiaGCST90018864chunk5-1.png]

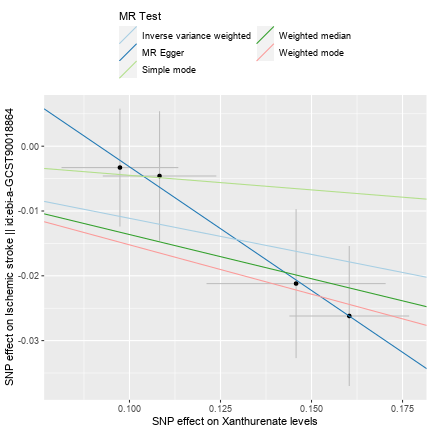

Supplement: Supplementary file 1 [file DataSheet1.ZIP › serum_metabolites/figure/Xanthurenate_levels_against_Ischemic_stroke__idebiaGCST90018864chunk6-1.png]

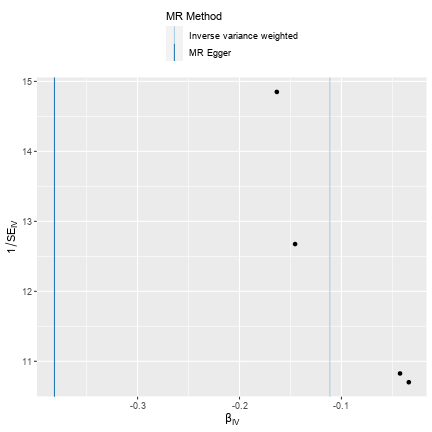

Supplement: Supplementary file 1 [file DataSheet1.ZIP › serum_metabolites/figure/Xanthurenate_levels_against_Ischemic_stroke__idebiaGCST90018864chunk7-1.png]

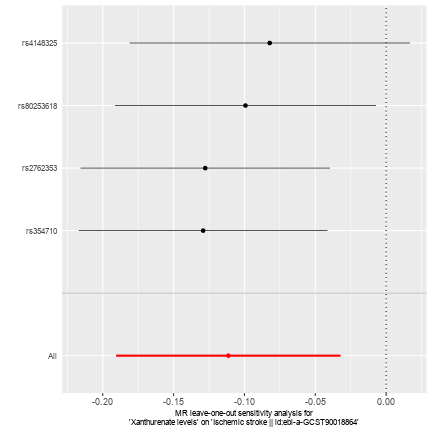

Supplement: Supplementary file 1 [file DataSheet1.ZIP › serum_metabolites/figure/Xanthurenate_levels_against_Ischemic_stroke__idebiaGCST90018864chunk8-1.png]

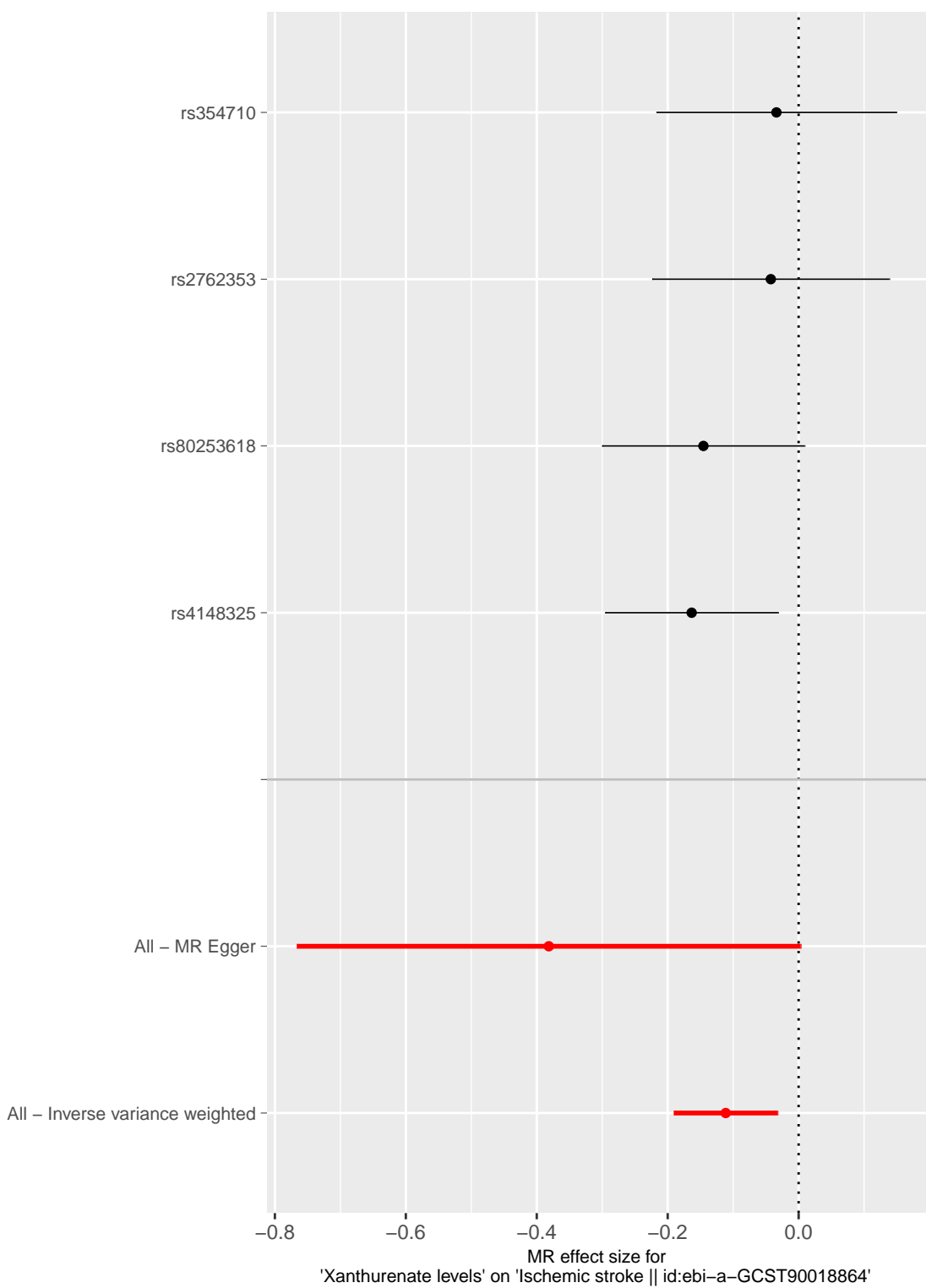

Supplement: Supplementary file 1 [file DataSheet1.ZIP › serum_metabolites/GCST90199626_forest_plot.pdf]

# MR Method

- Inverse variance weighted
- MR Egger

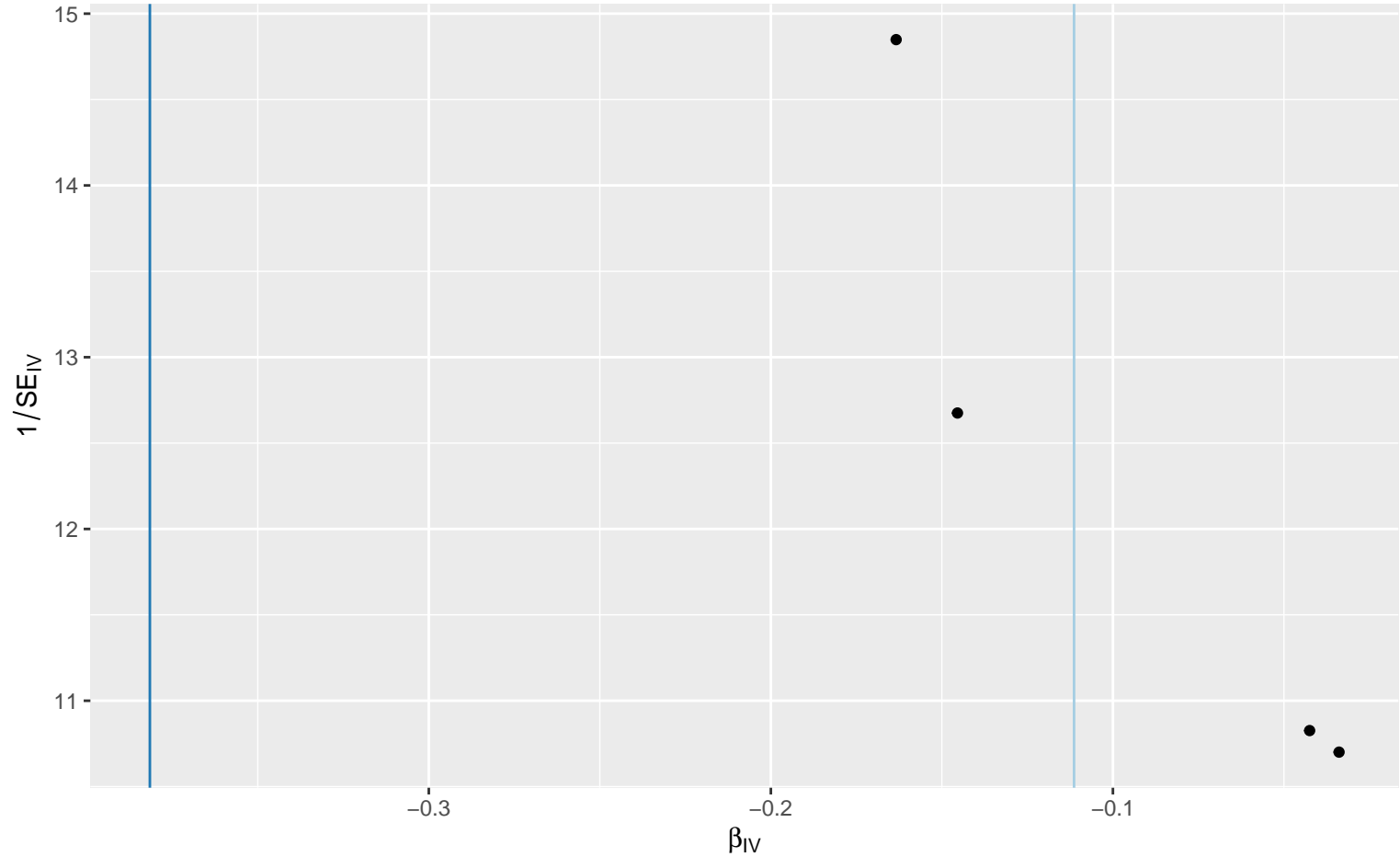

Supplement: Supplementary file 1 [file DataSheet1.ZIP › serum_metabolites/GCST90199626_funnel_plot.pdf]

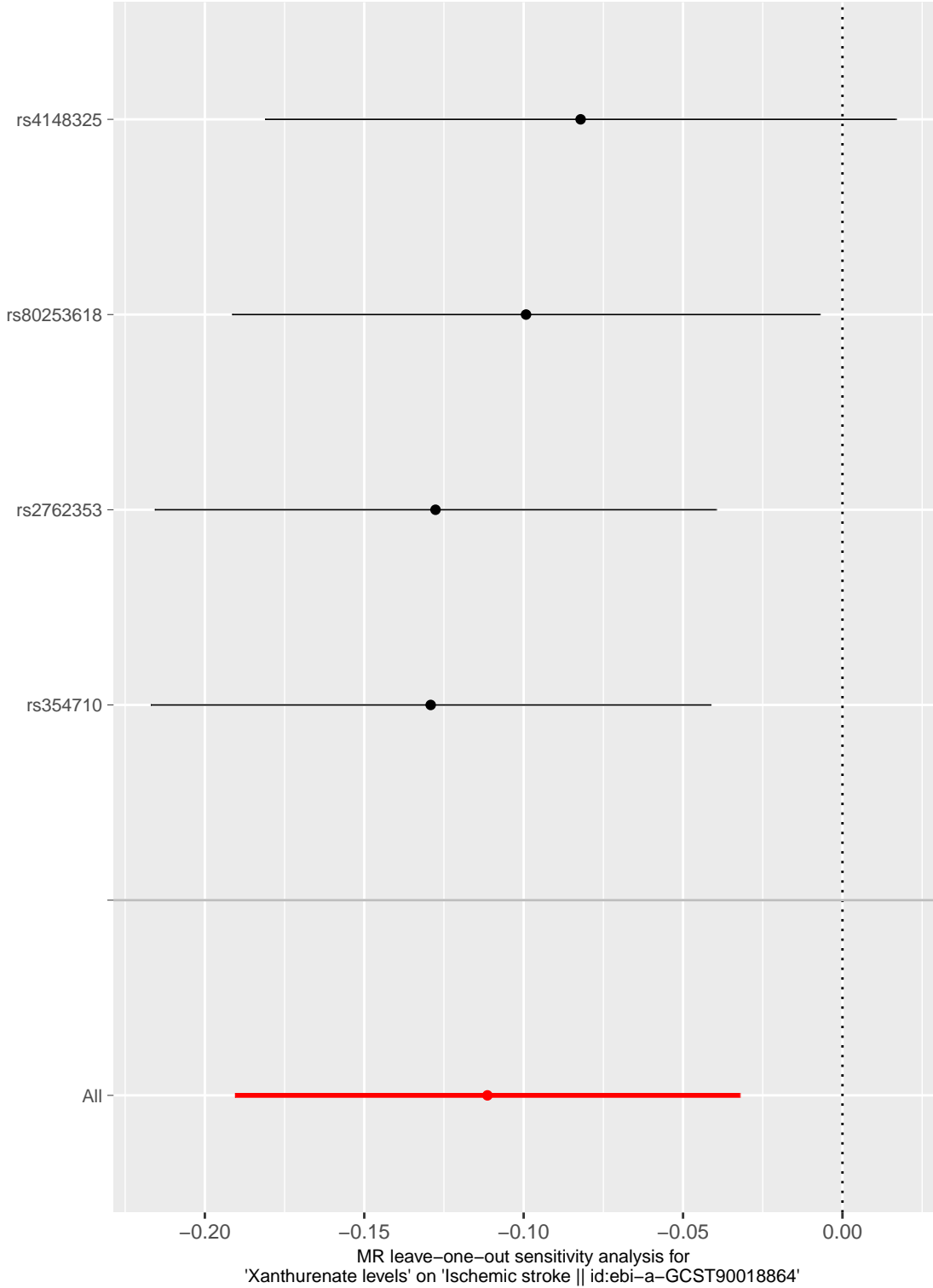

Supplement: Supplementary file 1 [file DataSheet1.ZIP › serum_metabolites/GCST90199626_leaveOneOut_plot.pdf]

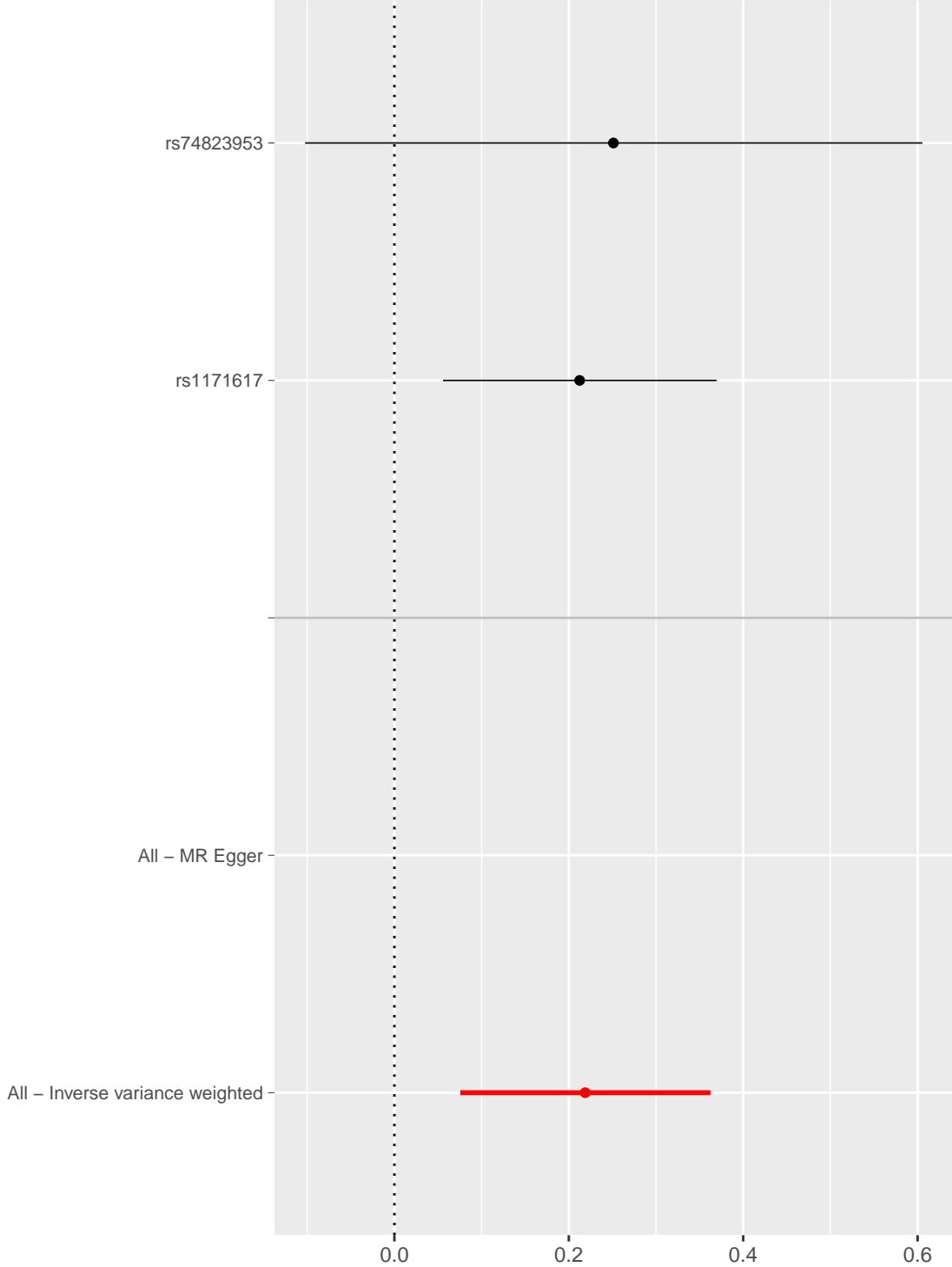

MR effect size for  
'Acetylcarnitine levels (Biocrates platform)' on 'Ischemic stroke || id:ebi-a-GCST9001886'

Supplement: Supplementary file 1 [file DataSheet1.ZIP › serum_metabolites/GCST90199669_forest_plot.pdf]

# MR Method

- Inverse variance weighted
- MR Egger

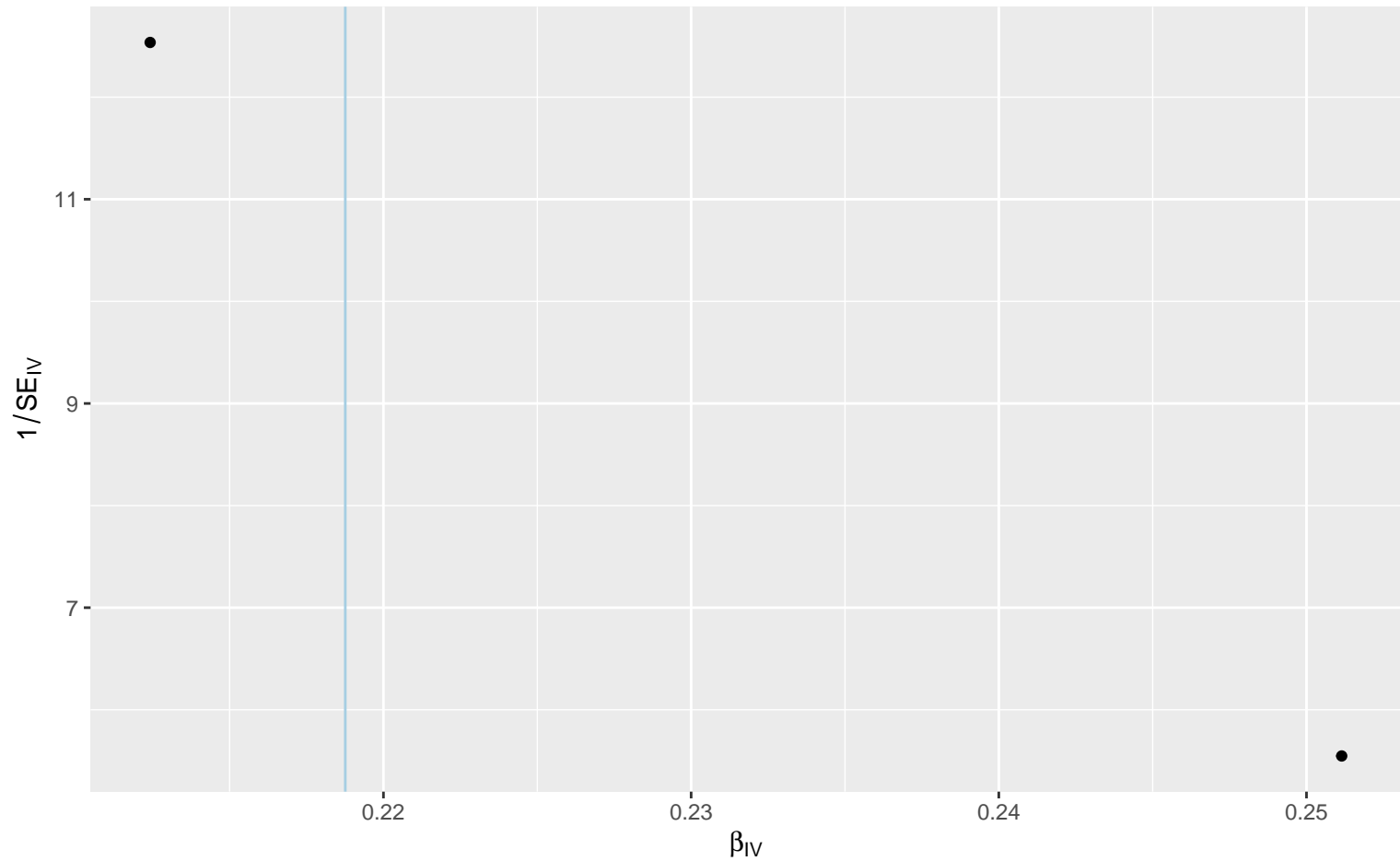

Supplement: Supplementary file 1 [file DataSheet1.ZIP › serum_metabolites/GCST90199669_funnel_plot.pdf]

# MR Test

Inverse variance weighted

SNP effect on Ischemic stroke || id:ebi-a-GCST90018864

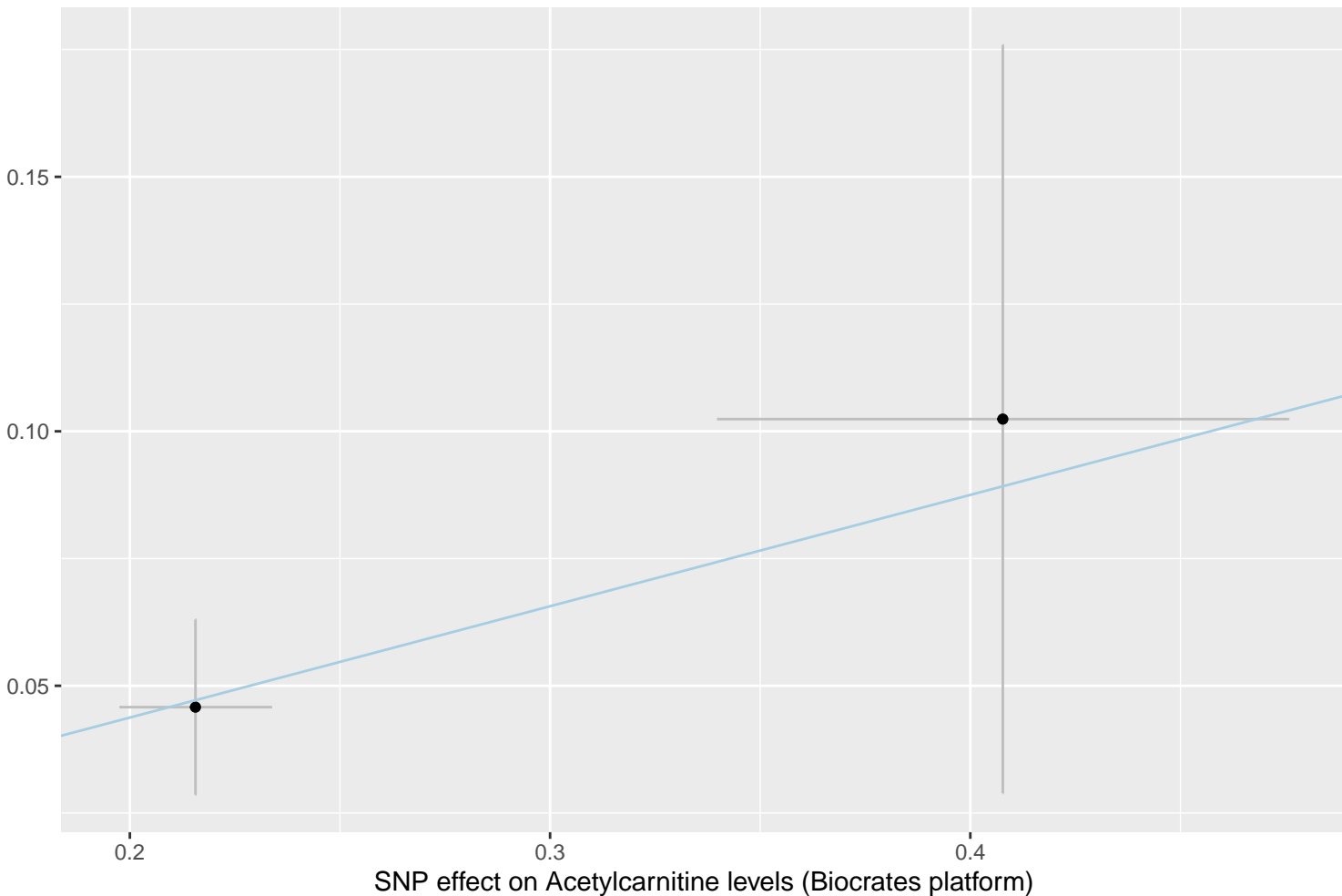

Supplement: Supplementary file 1 [file DataSheet1.ZIP › serum_metabolites/GCST90199669_scatter_plot.pdf]

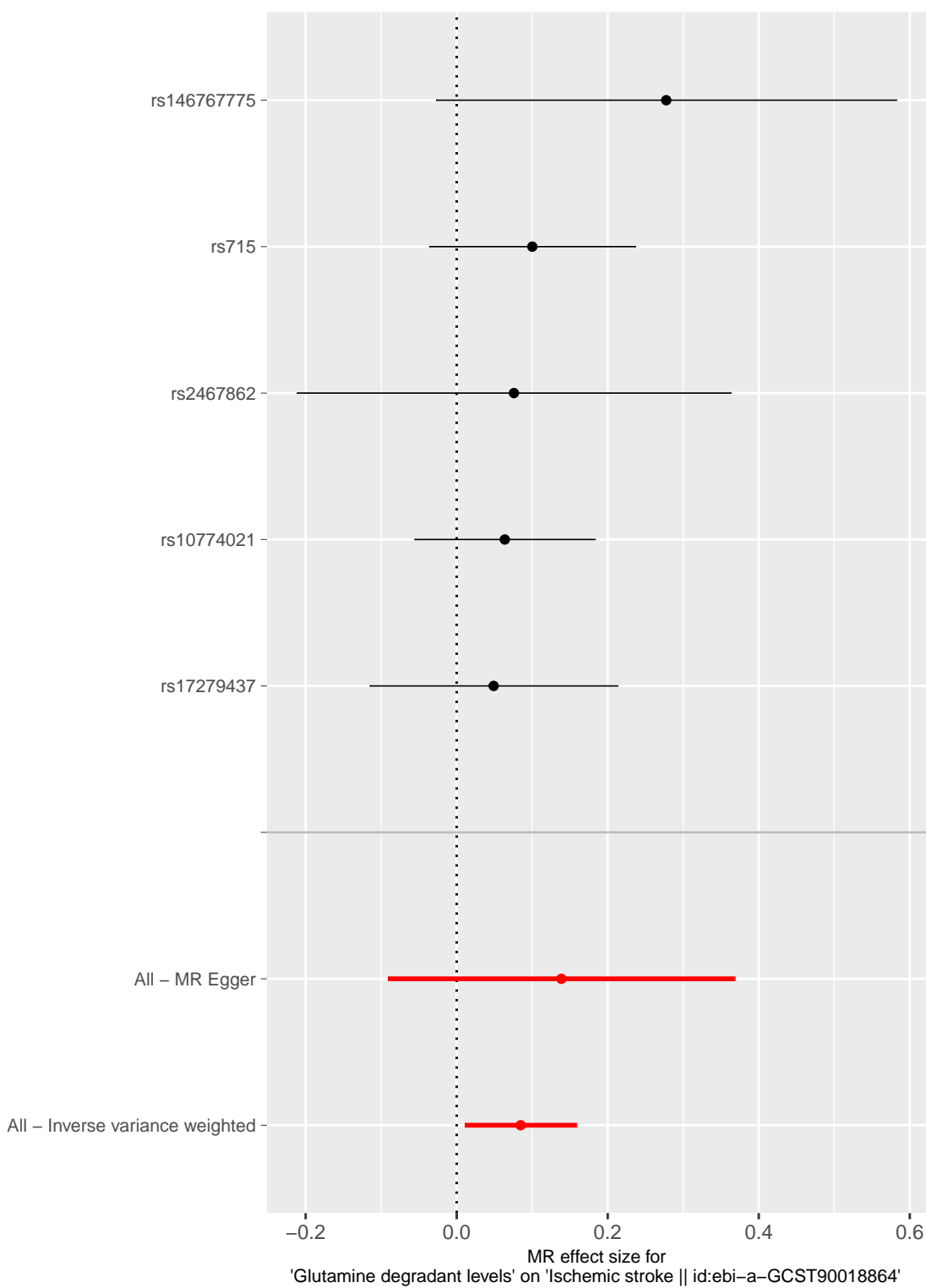

Supplement: Supplementary file 1 [file DataSheet1.ZIP › serum_metabolites/GCST90199782_forest_plot.pdf]

# MR Method

- Inverse variance weighted
- MR Egger

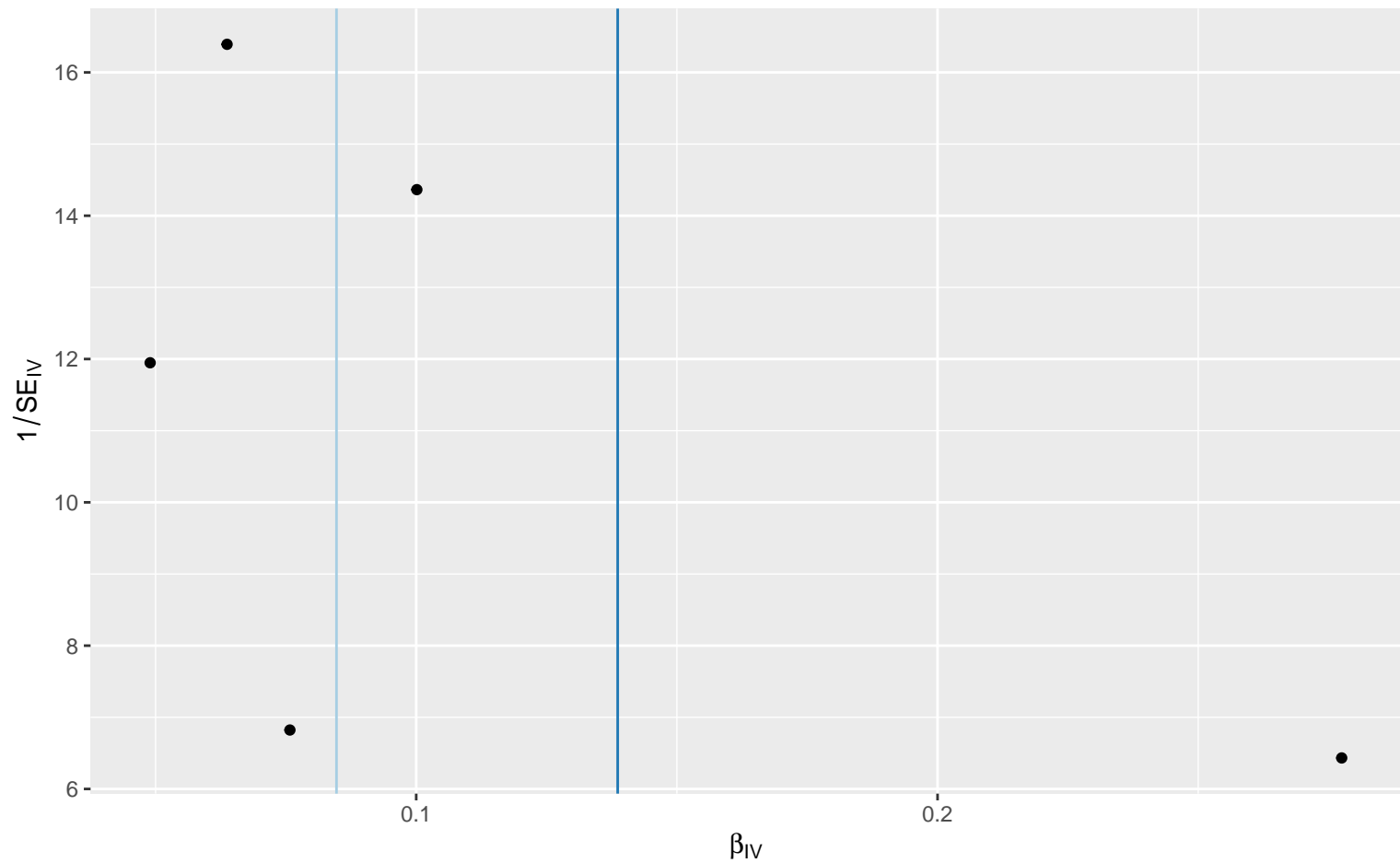

Supplement: Supplementary file 1 [file DataSheet1.ZIP › serum_metabolites/GCST90199782_funnel_plot.pdf]

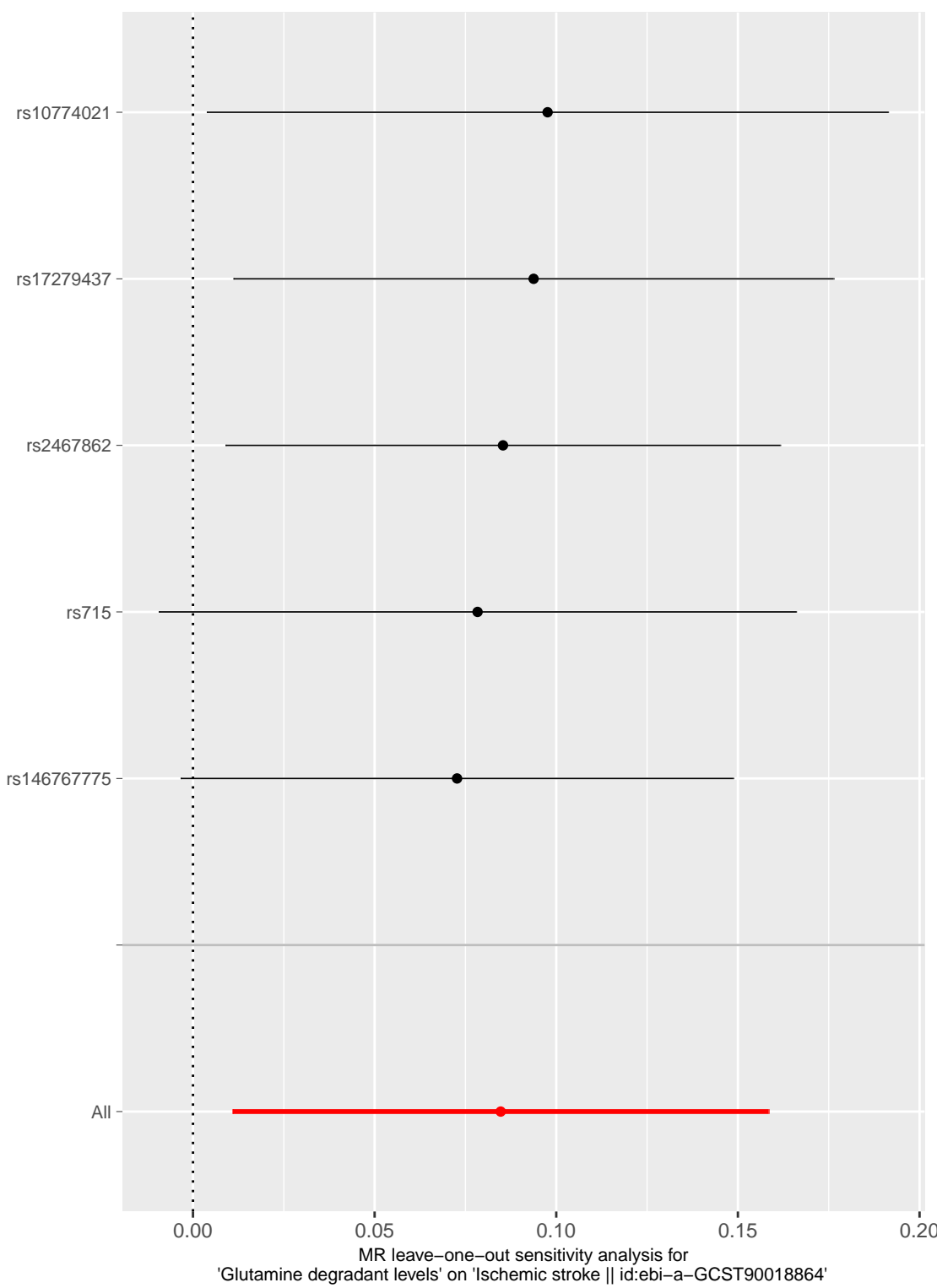

Supplement: Supplementary file 1 [file DataSheet1.ZIP › serum_metabolites/GCST90199782_leaveOneOut_plot.pdf]

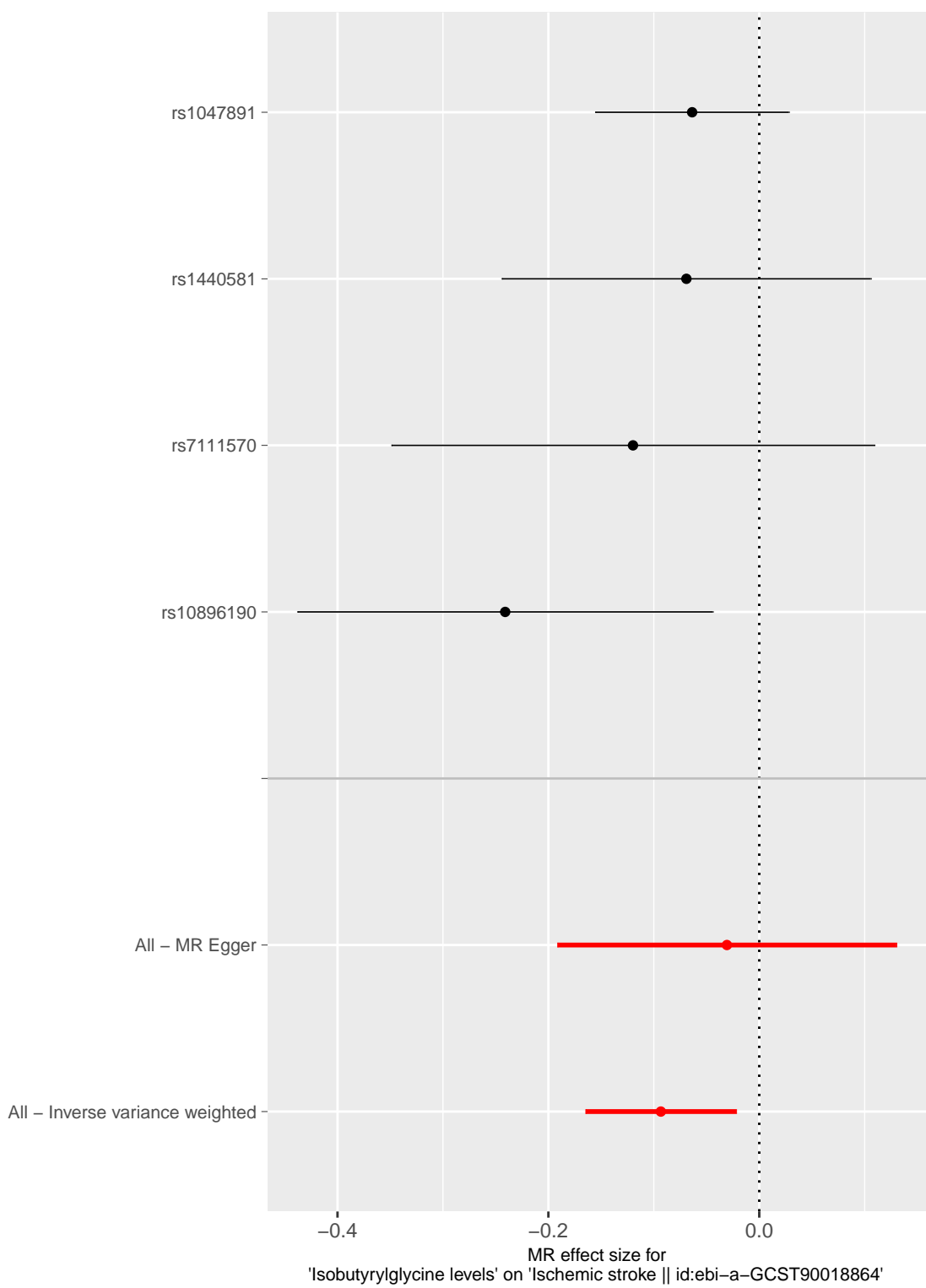

Supplement: Supplementary file 1 [file DataSheet1.ZIP › serum_metabolites/GCST90199798_forest_plot.pdf]

# MR Method

- Inverse variance weighted
- MR Egger

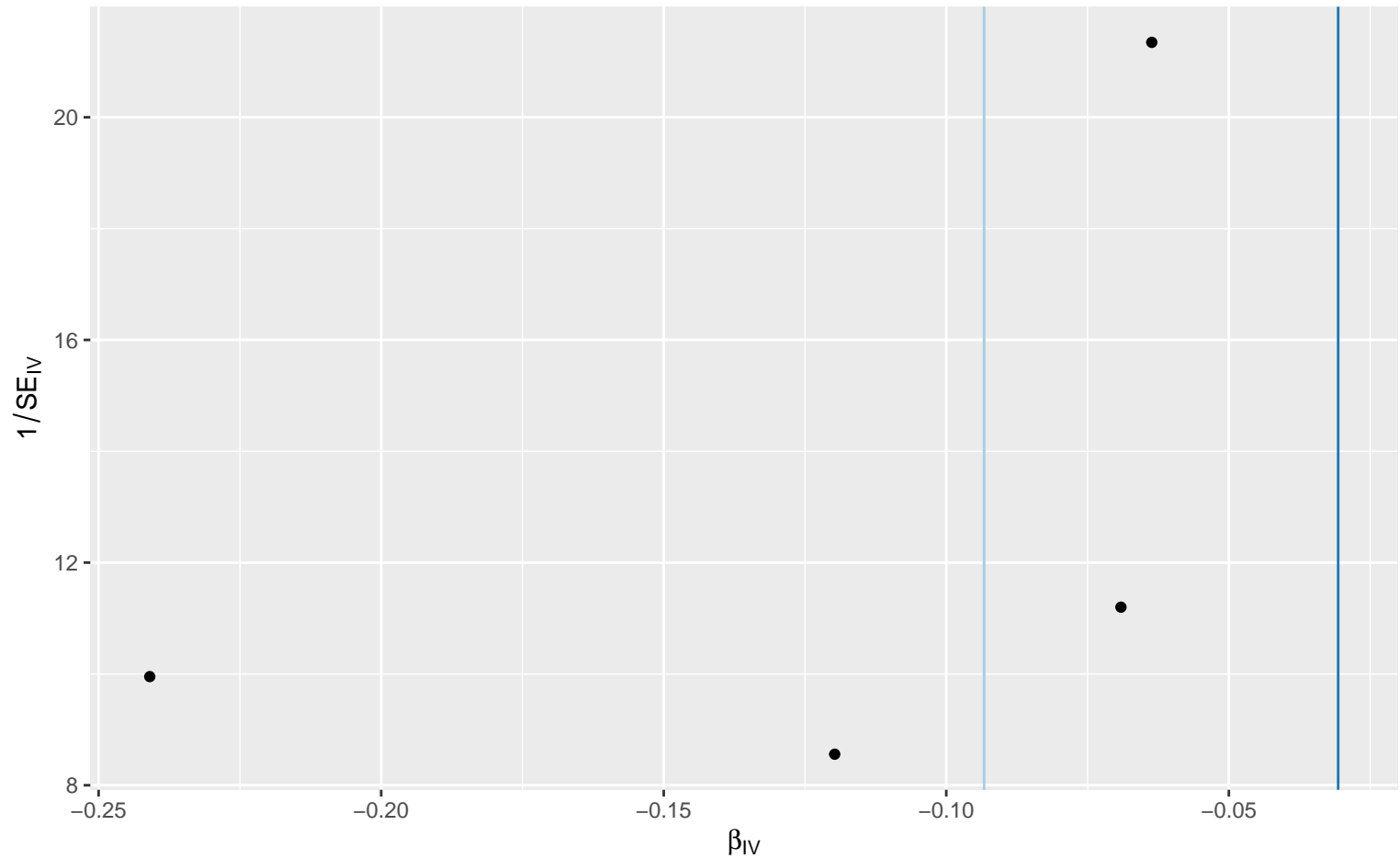

Supplement: Supplementary file 1 [file DataSheet1.ZIP › serum_metabolites/GCST90199798_funnel_plot.pdf]

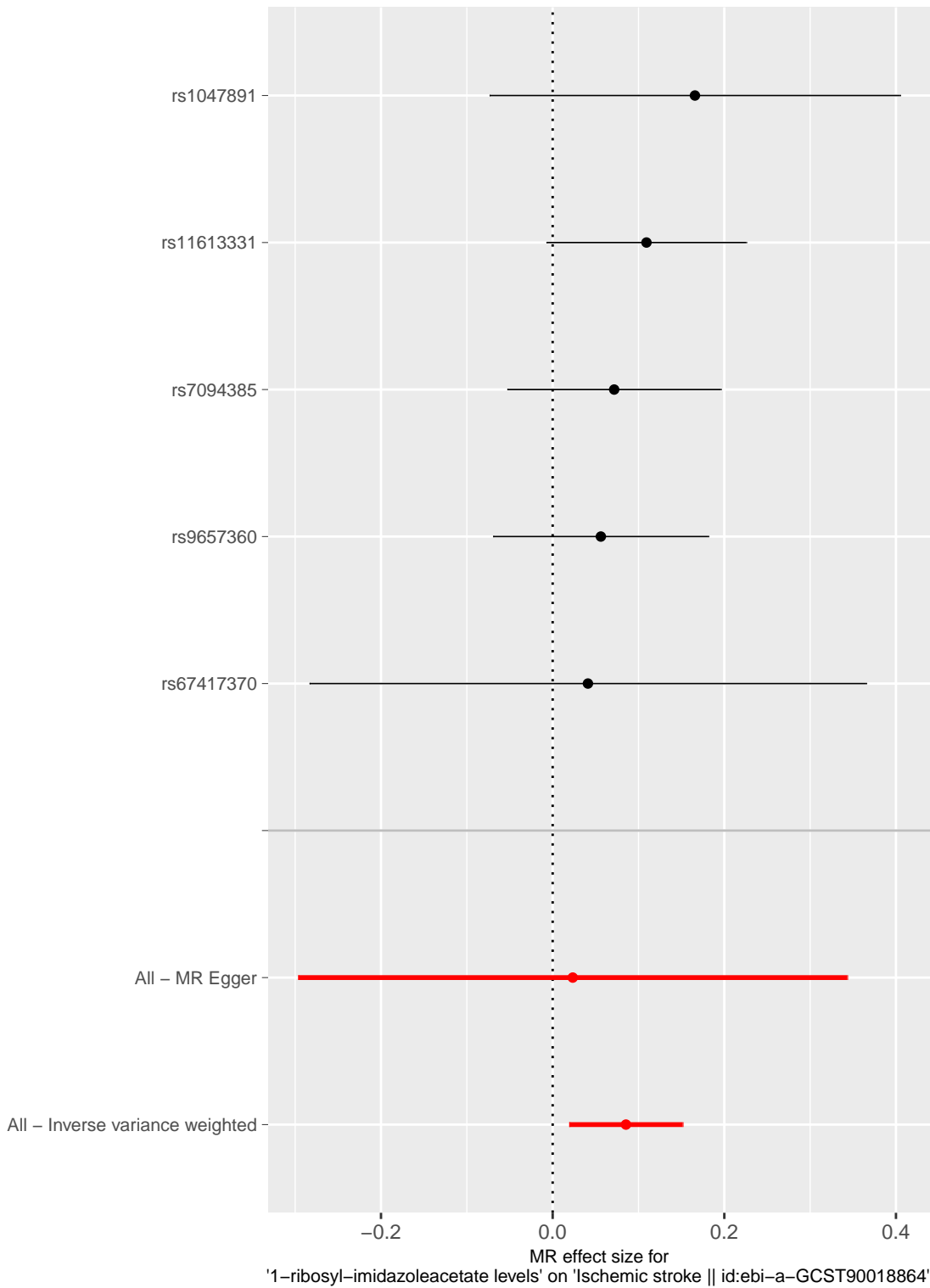

Supplement: Supplementary file 1 [file DataSheet1.ZIP › serum_metabolites/GCST90199815_forest_plot.pdf]

# MR Method

- Inverse variance weighted
- MR Egger

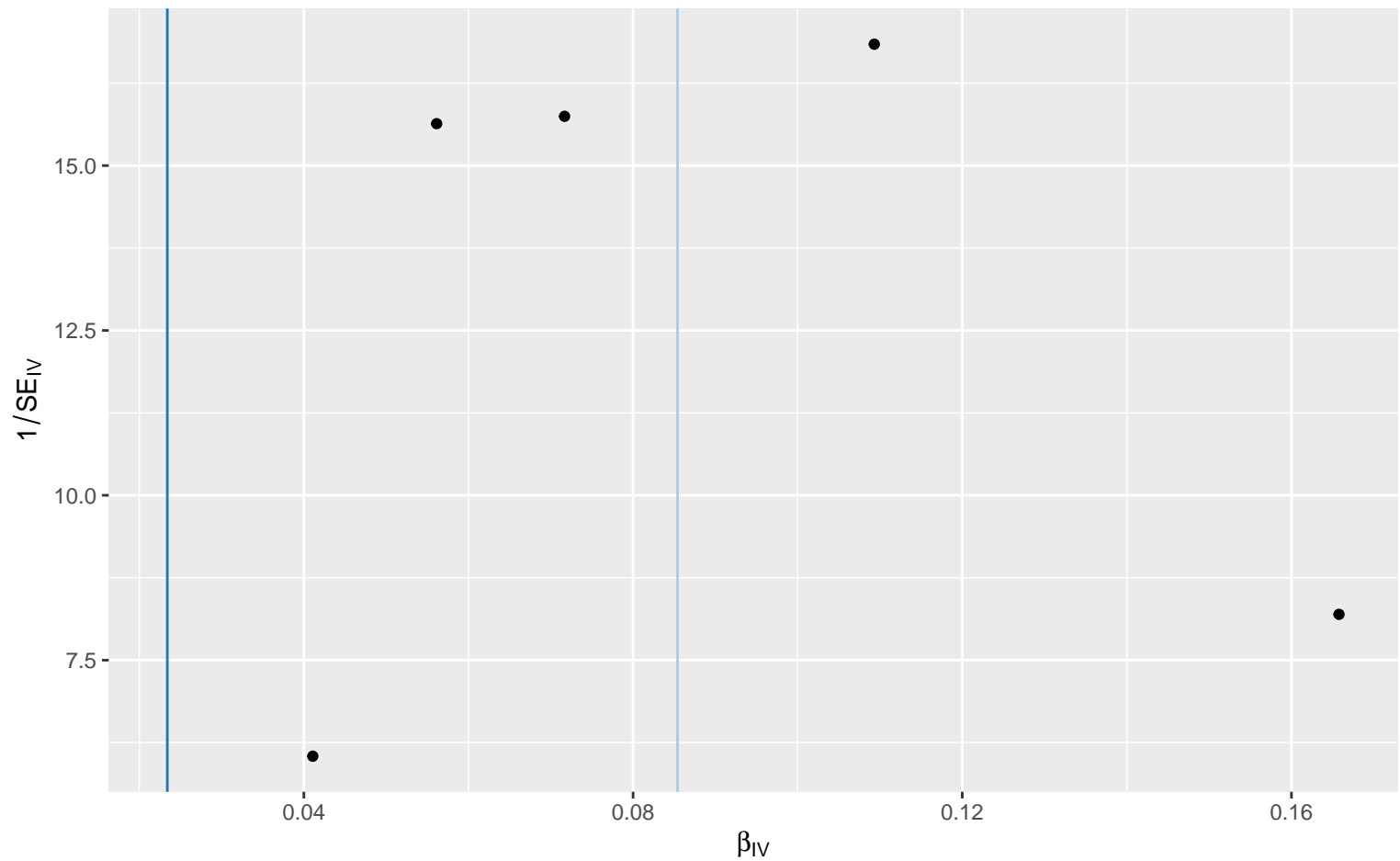

Supplement: Supplementary file 1 [file DataSheet1.ZIP › serum_metabolites/GCST90199815_funnel_plot.pdf]

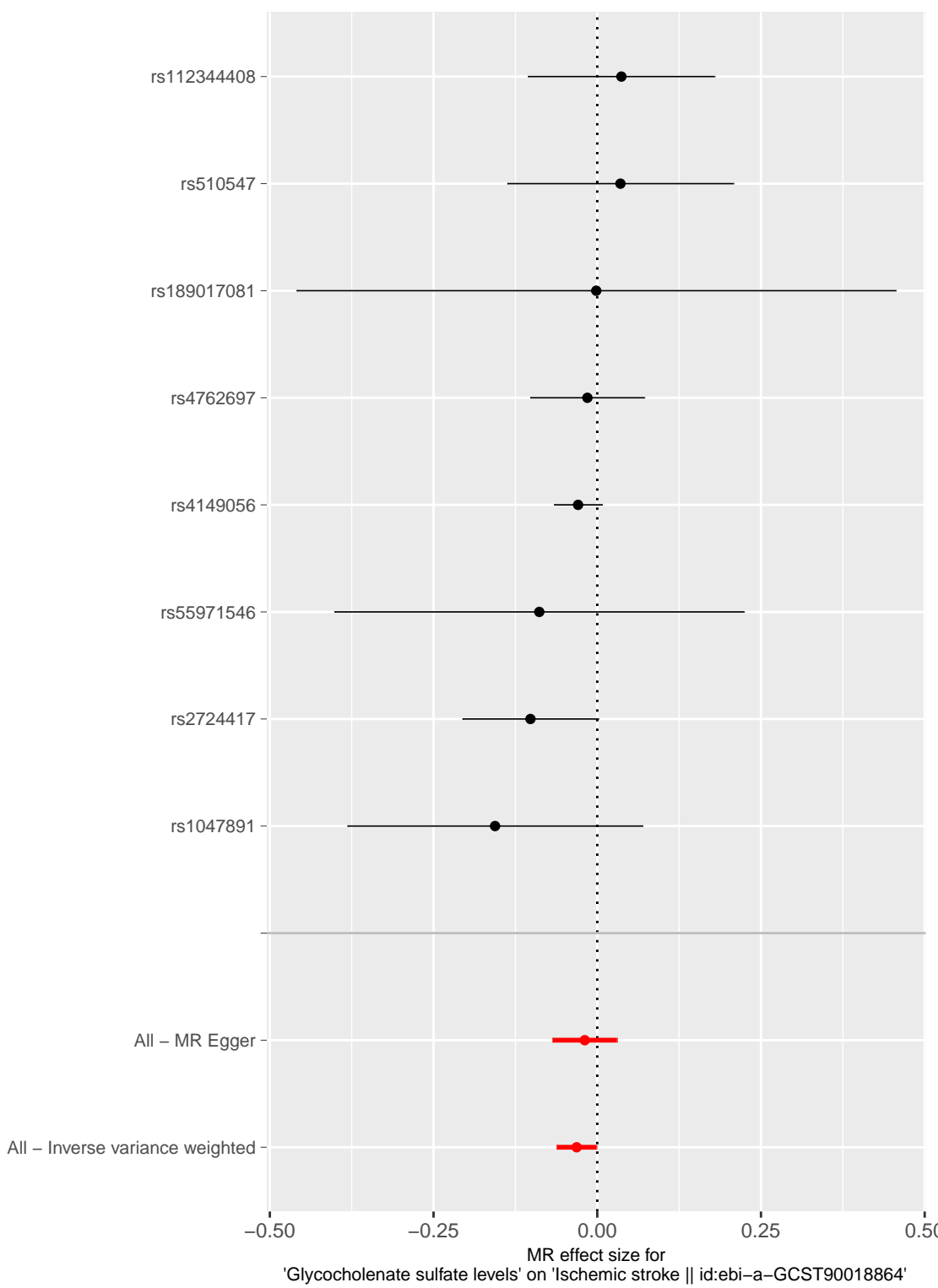

Supplement: Supplementary file 1 [file DataSheet1.ZIP › serum_metabolites/GCST90199841_forest_plot.pdf]

# MR Method

- Inverse variance weighted
- MR Egger

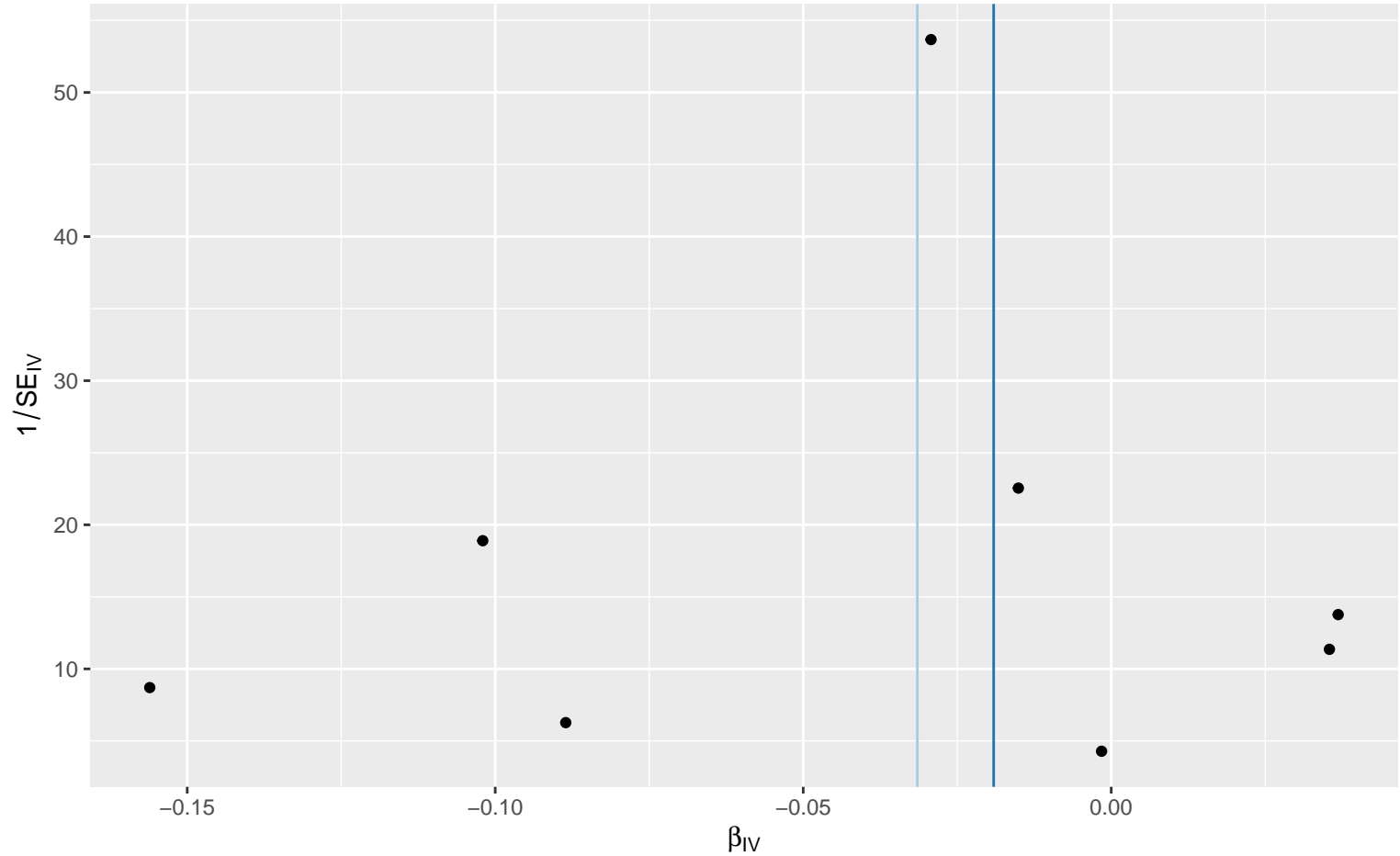

Supplement: Supplementary file 1 [file DataSheet1.ZIP › serum_metabolites/GCST90199841_funnel_plot.pdf]

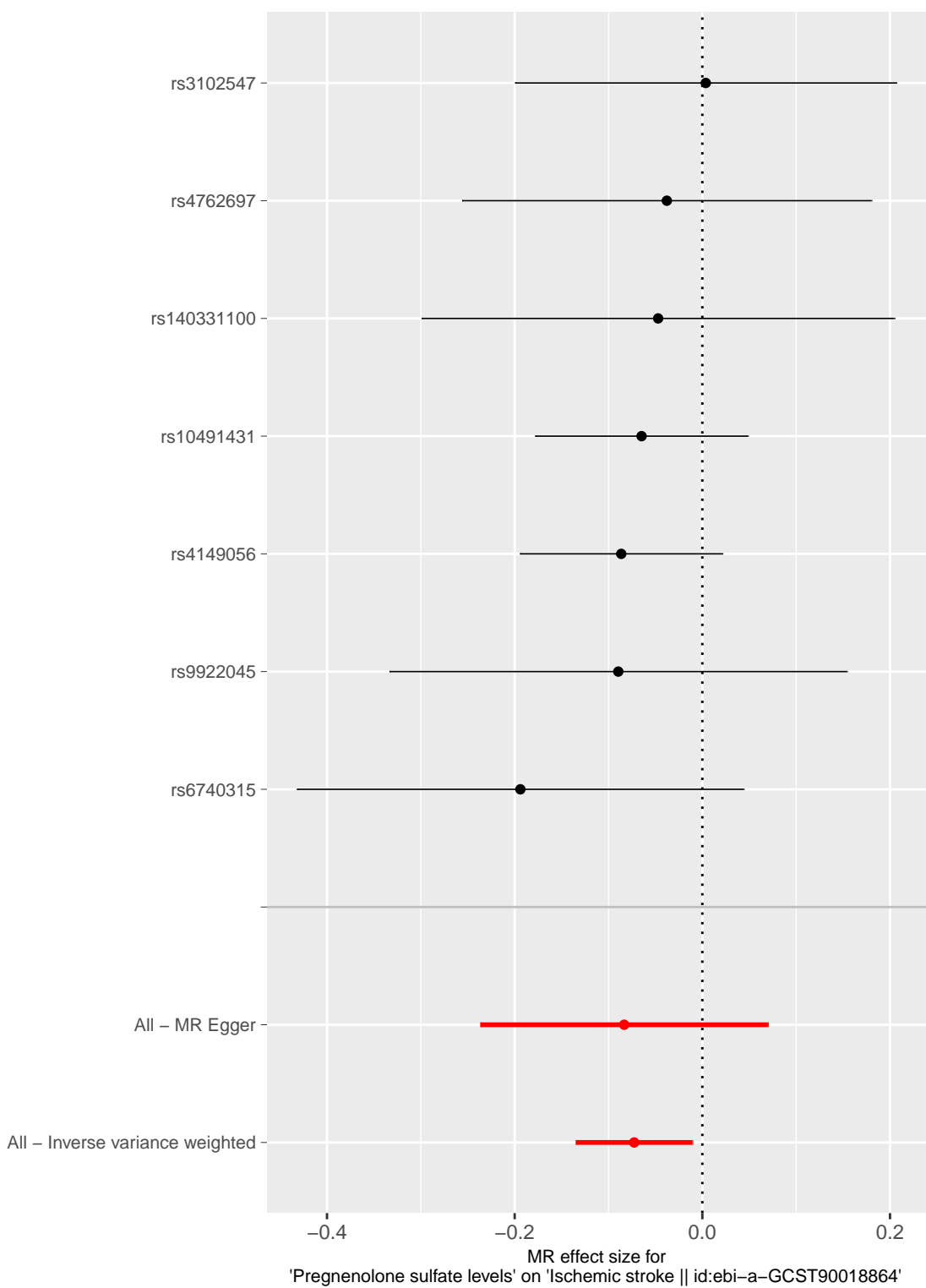

Supplement: Supplementary file 1 [file DataSheet1.ZIP › serum_metabolites/GCST90199873_forest_plot.pdf]

# MR Method

- Inverse variance weighted
- MR Egger

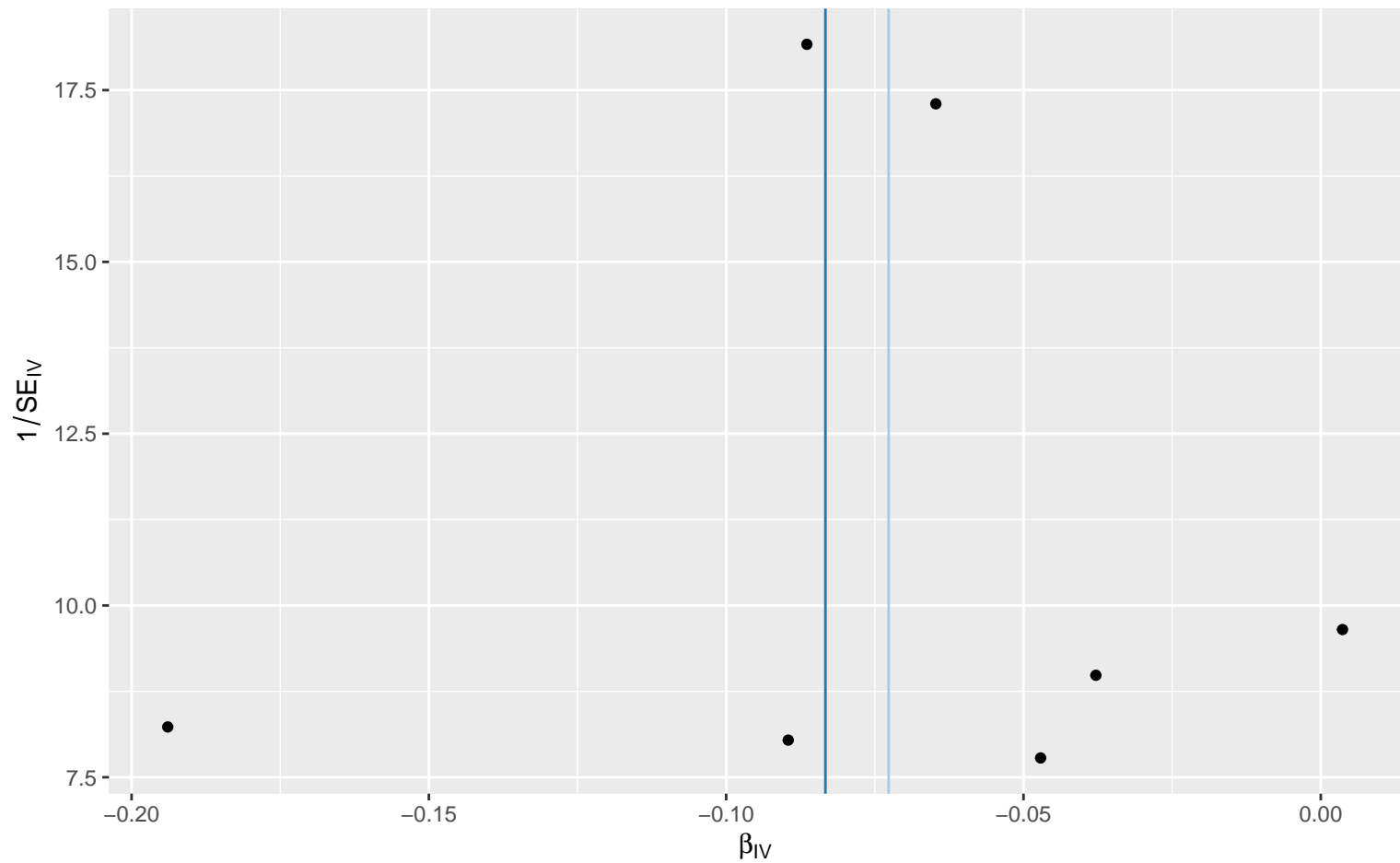

Supplement: Supplementary file 1 [file DataSheet1.ZIP › serum_metabolites/GCST90199873_funnel_plot.pdf]

# MR Test

- Inverse variance weighted
- MR Egger
- Simple mode
- Weighted mode

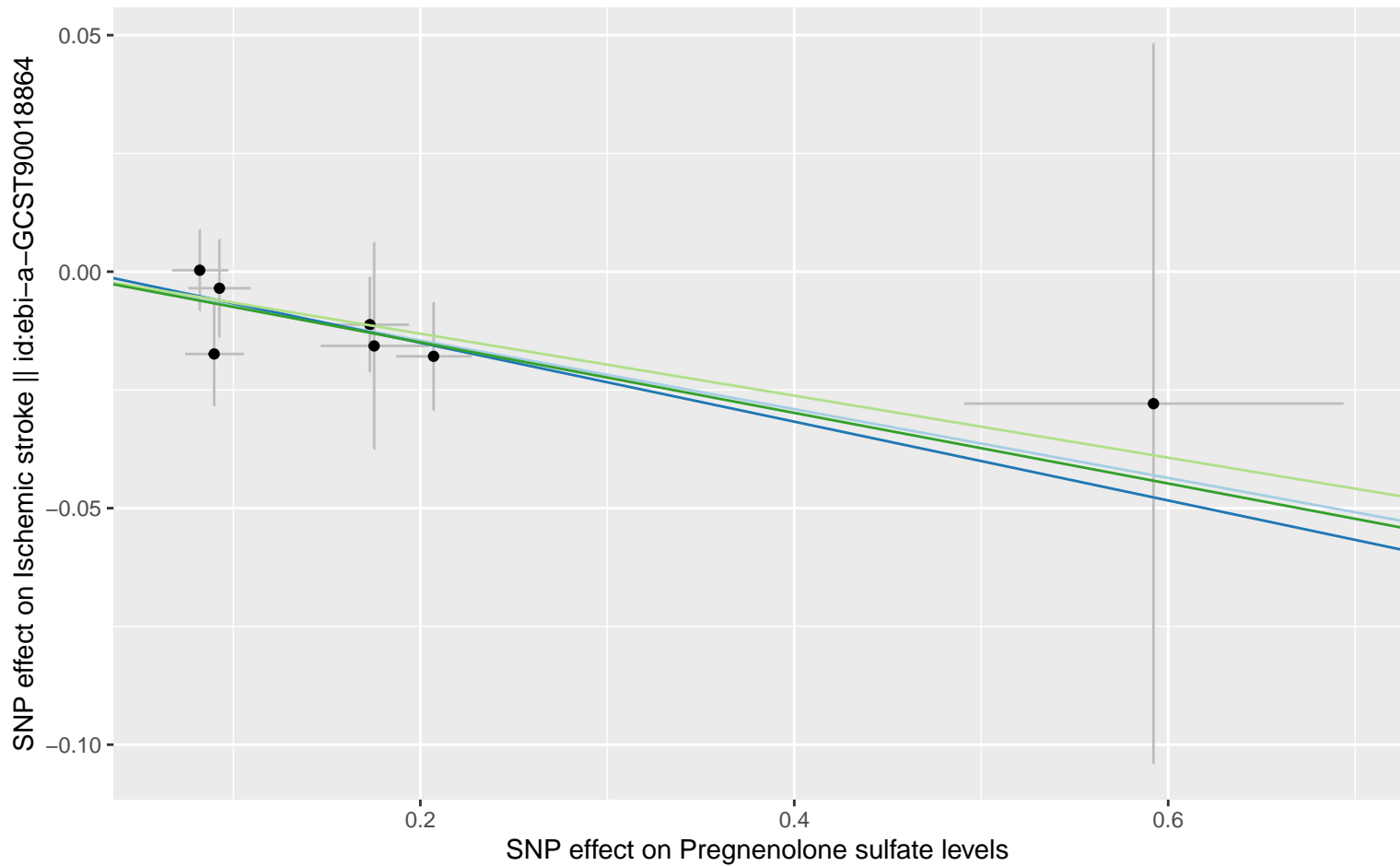

Supplement: Supplementary file 1 [file DataSheet1.ZIP › serum_metabolites/GCST90199873_scatter_plot.pdf]

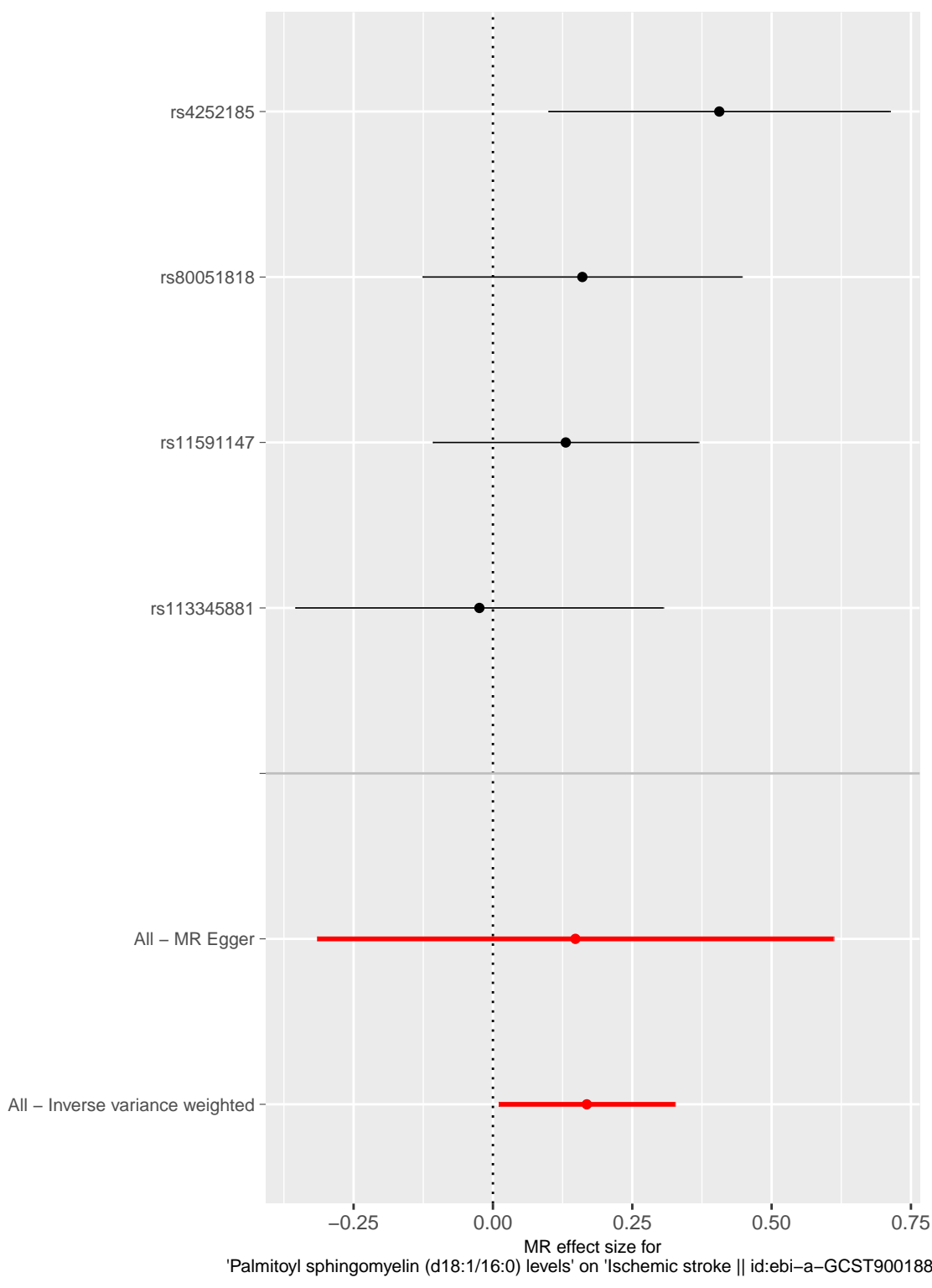

Supplement: Supplementary file 1 [file DataSheet1.ZIP › serum_metabolites/GCST90199876_forest_plot.pdf]

# MR Method

- Inverse variance weighted
- MR Egger

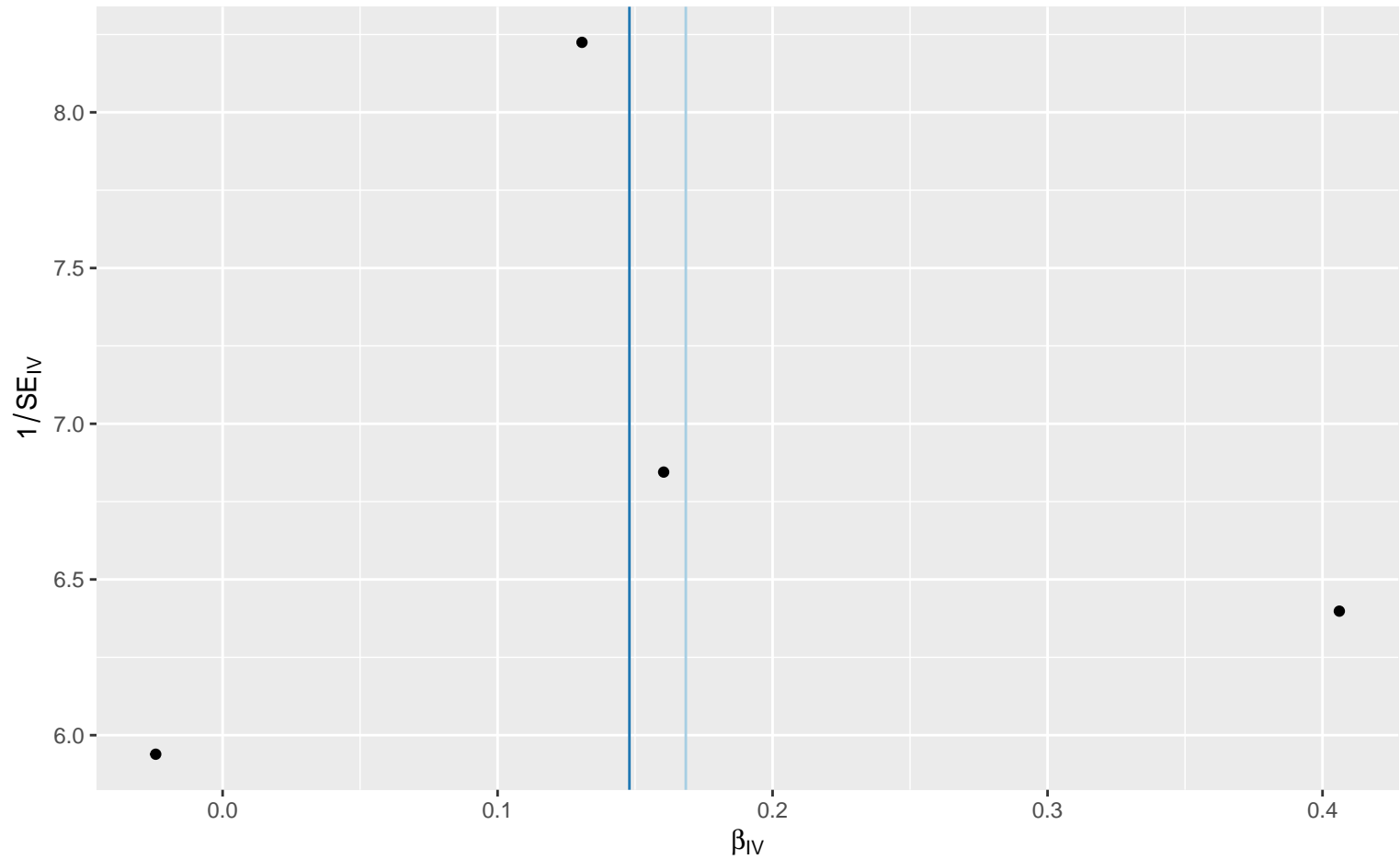

Supplement: Supplementary file 1 [file DataSheet1.ZIP › serum_metabolites/GCST90199876_funnel_plot.pdf]

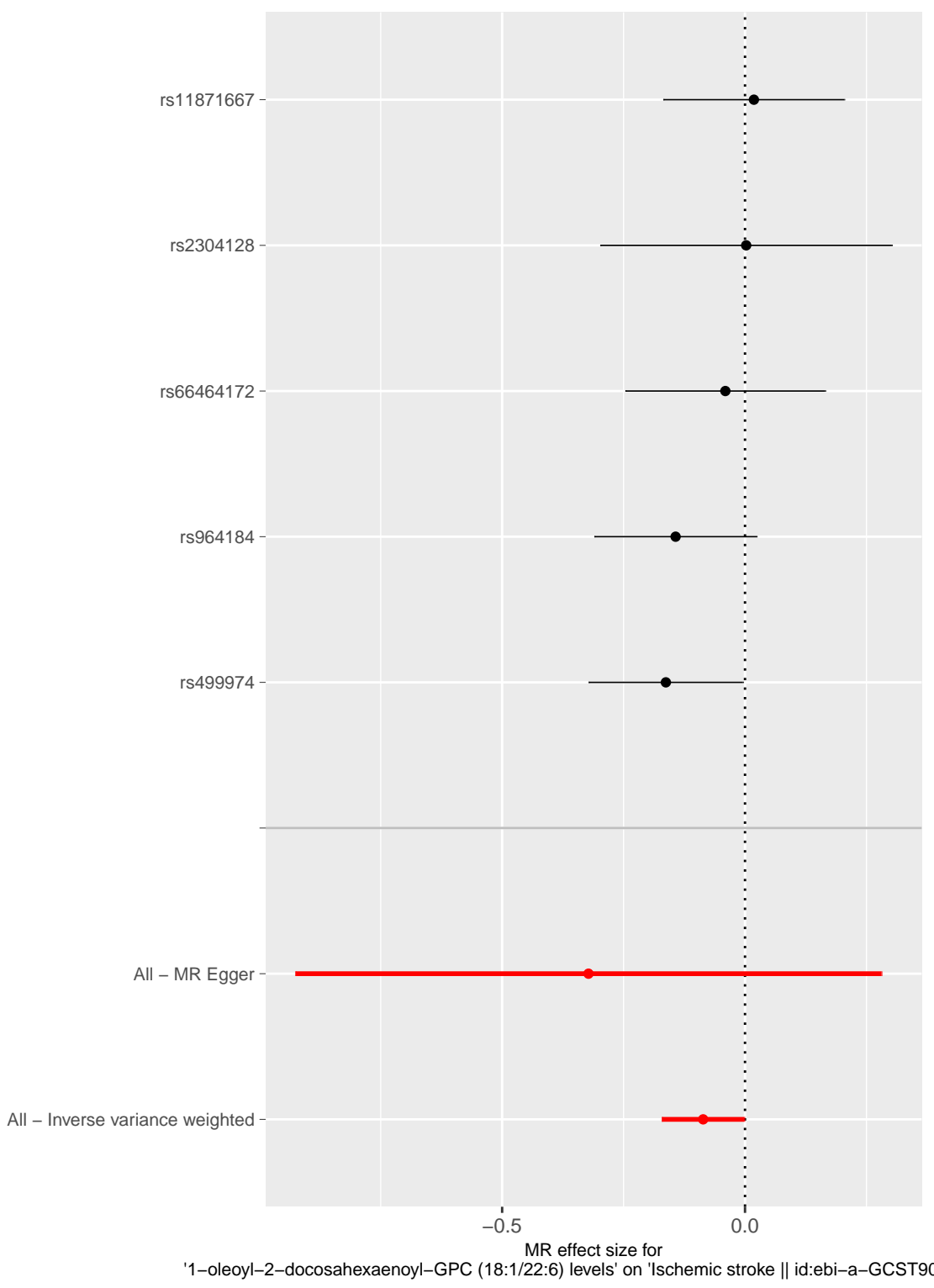

Supplement: Supplementary file 1 [file DataSheet1.ZIP › serum_metabolites/GCST90200081_forest_plot.pdf]

# MR Method

- Inverse variance weighted
- MR Egger

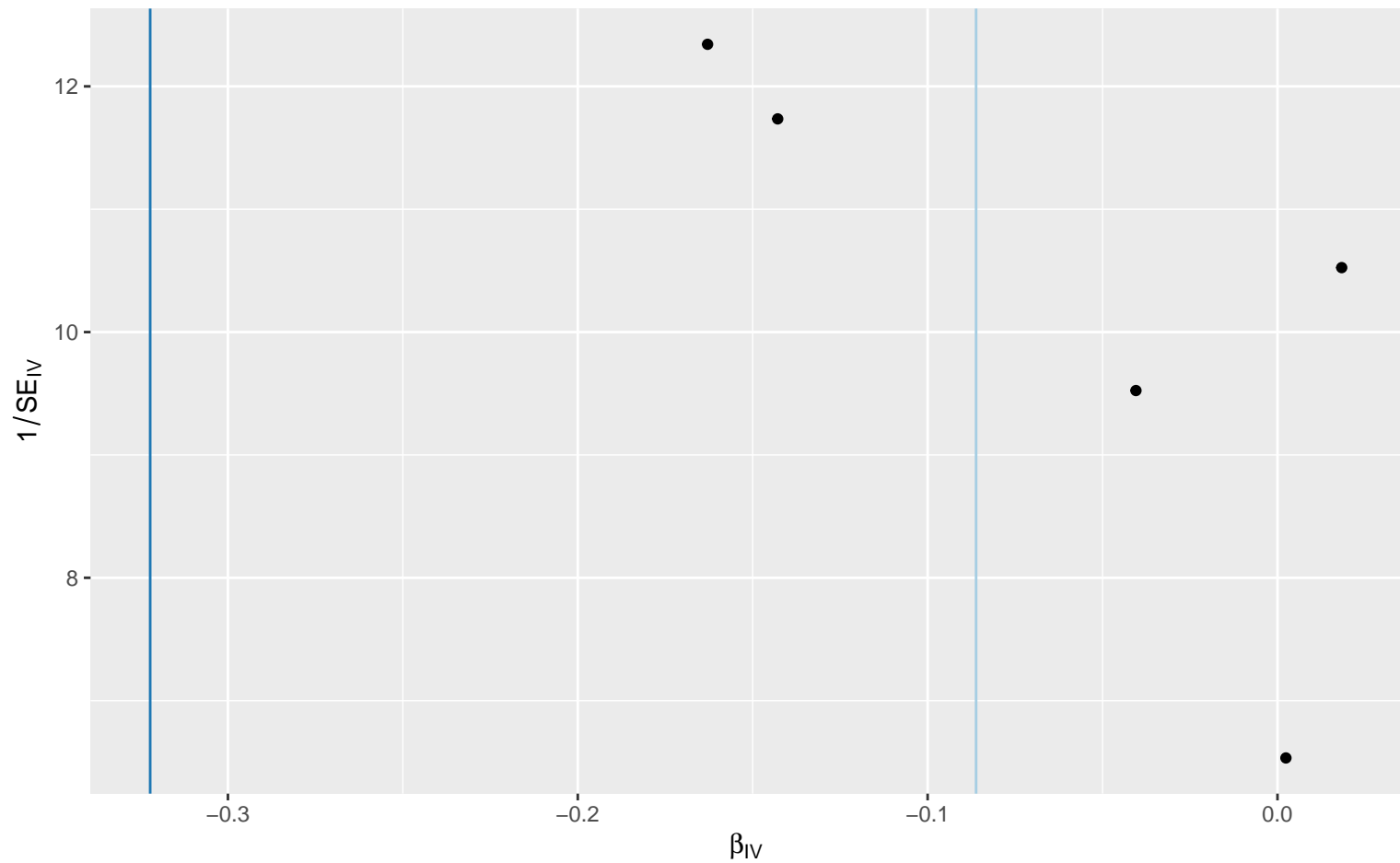

Supplement: Supplementary file 1 [file DataSheet1.ZIP › serum_metabolites/GCST90200081_funnel_plot.pdf]

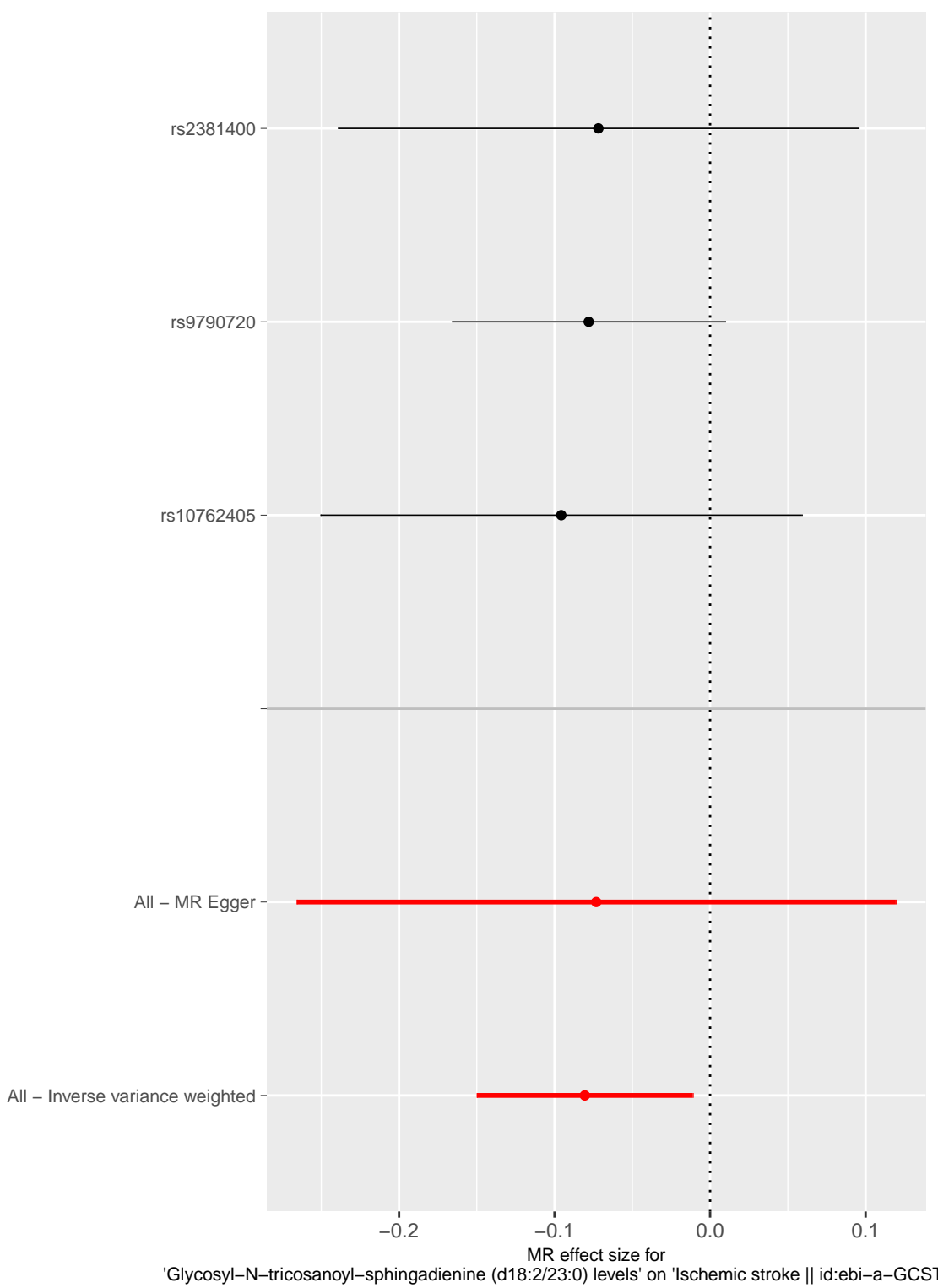

Supplement: Supplementary file 1 [file DataSheet1.ZIP › serum_metabolites/GCST90200114_forest_plot.pdf]

# MR Method

- Inverse variance weighted
- MR Egger

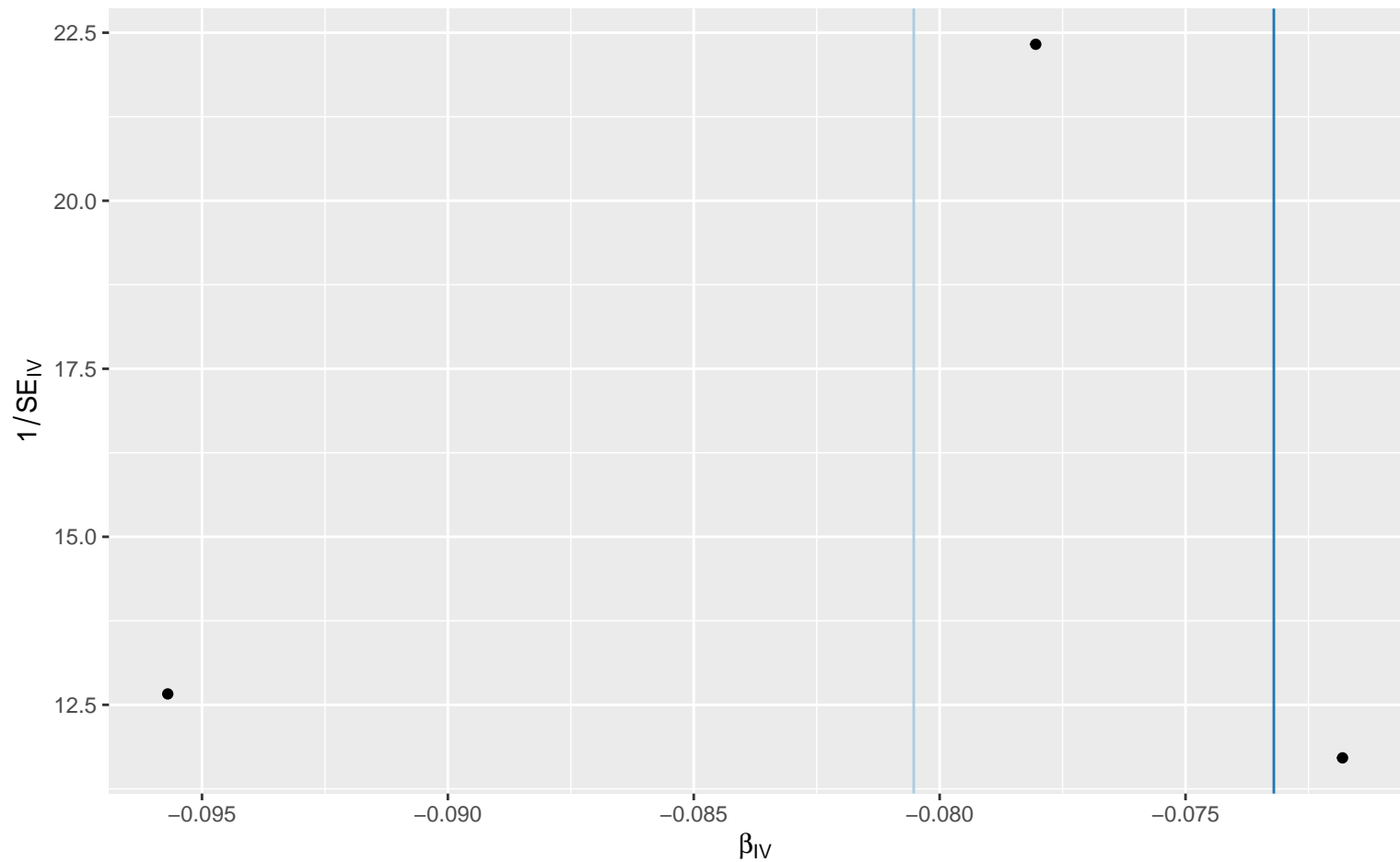

Supplement: Supplementary file 1 [file DataSheet1.ZIP › serum_metabolites/GCST90200114_funnel_plot.pdf]

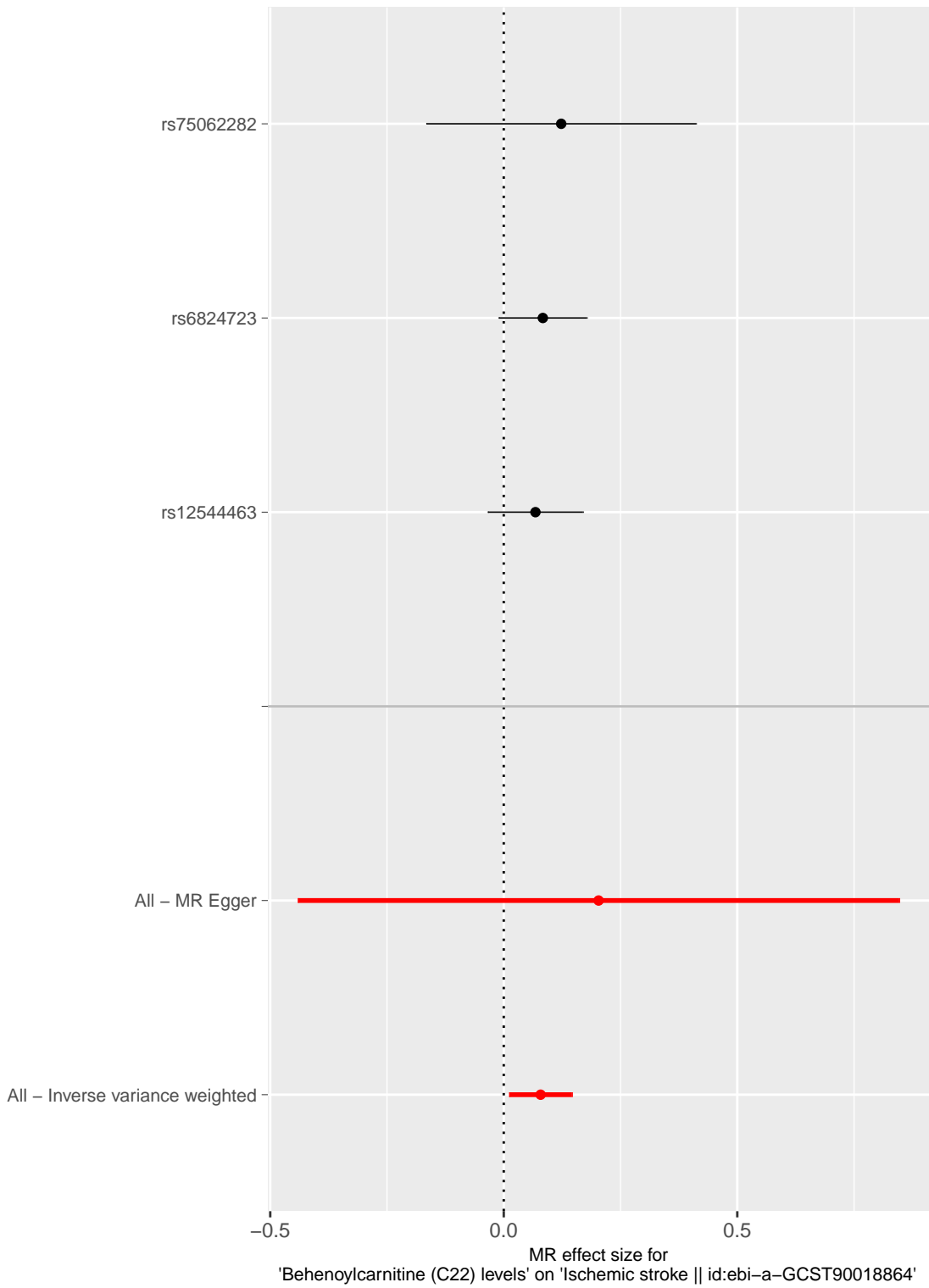

Supplement: Supplementary file 1 [file DataSheet1.ZIP › serum_metabolites/GCST90200127_forest_plot.pdf]

# MR Method

- Inverse variance weighted
- MR Egger

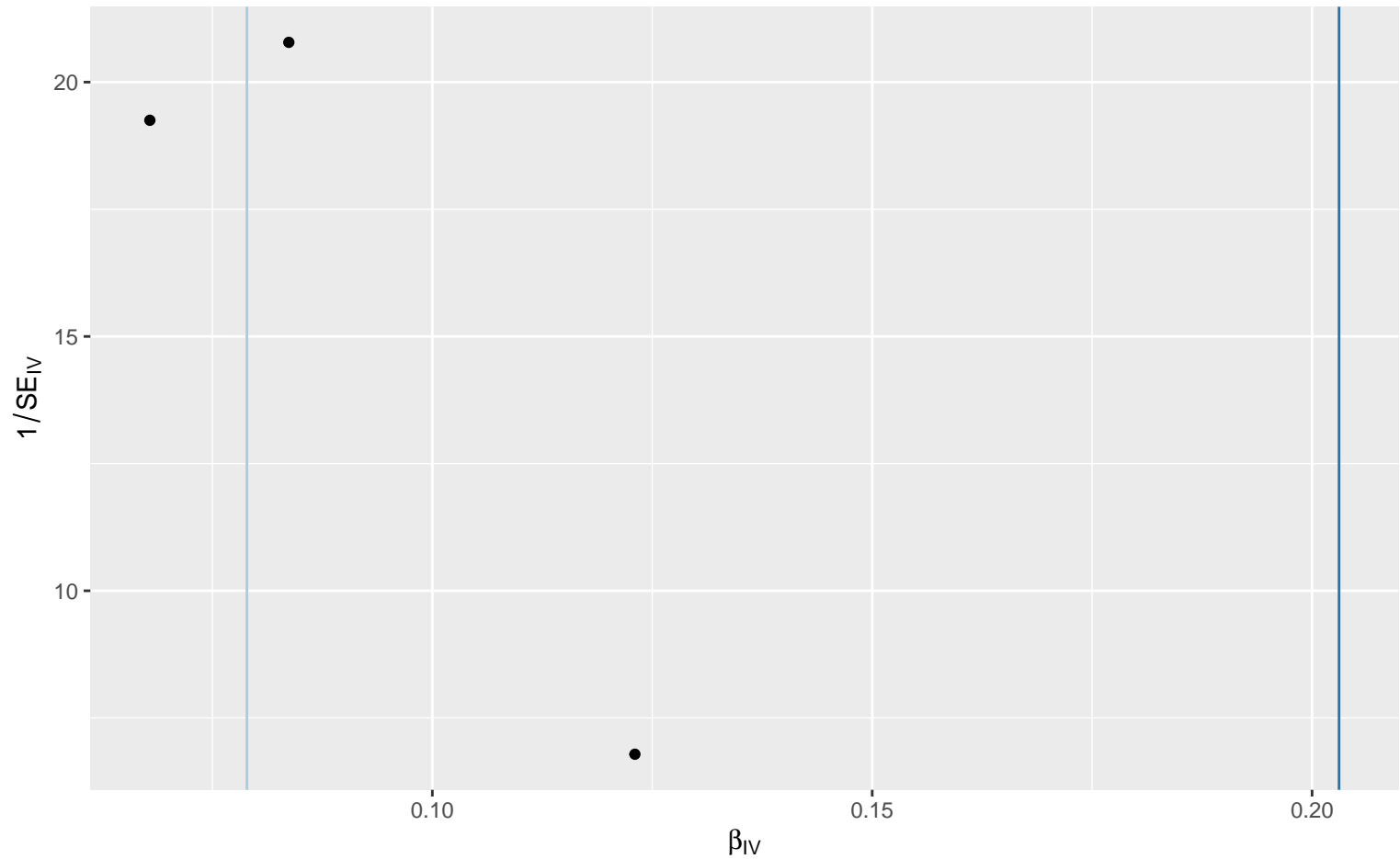

Supplement: Supplementary file 1 [file DataSheet1.ZIP › serum_metabolites/GCST90200127_funnel_plot.pdf]

# MR Test

- Inverse variance weighted
- MR Egger
- Simple mode
- Weighted mode

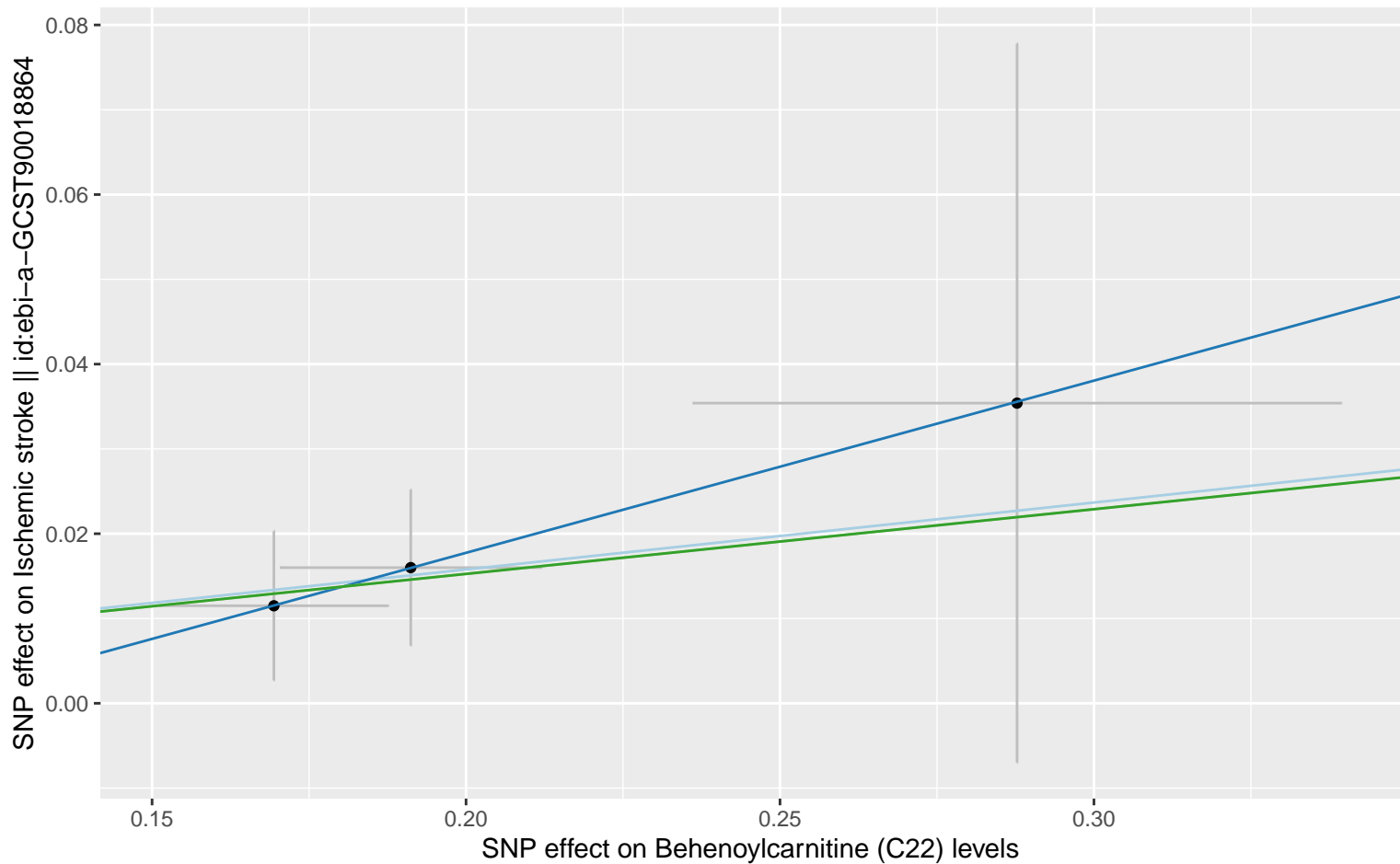

Supplement: Supplementary file 1 [file DataSheet1.ZIP › serum_metabolites/GCST90200127_scatter_plot.pdf]

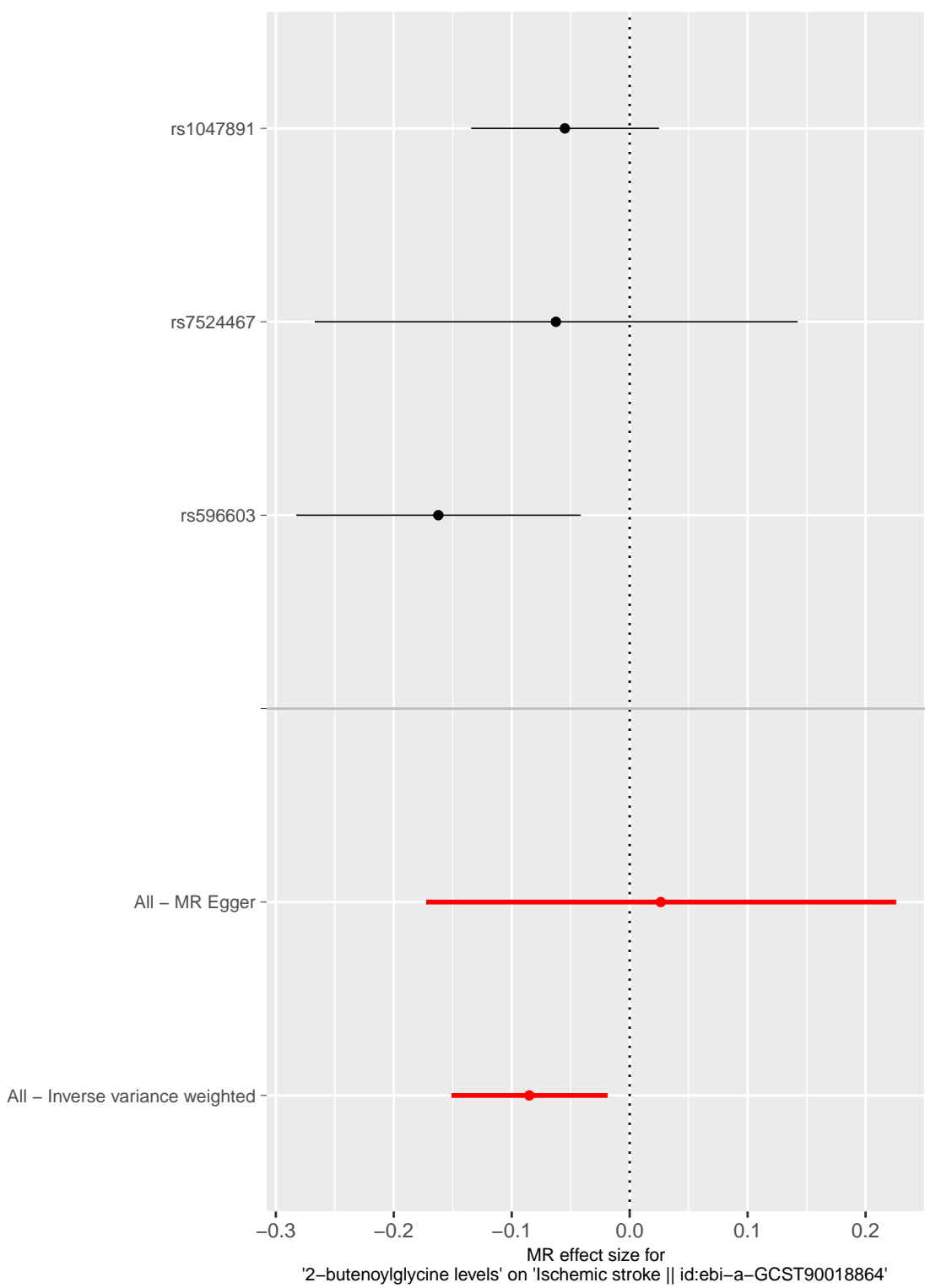

Supplement: Supplementary file 1 [file DataSheet1.ZIP › serum_metabolites/GCST90200152_forest_plot.pdf]

# MR Method

- Inverse variance weighted
- MR Egger

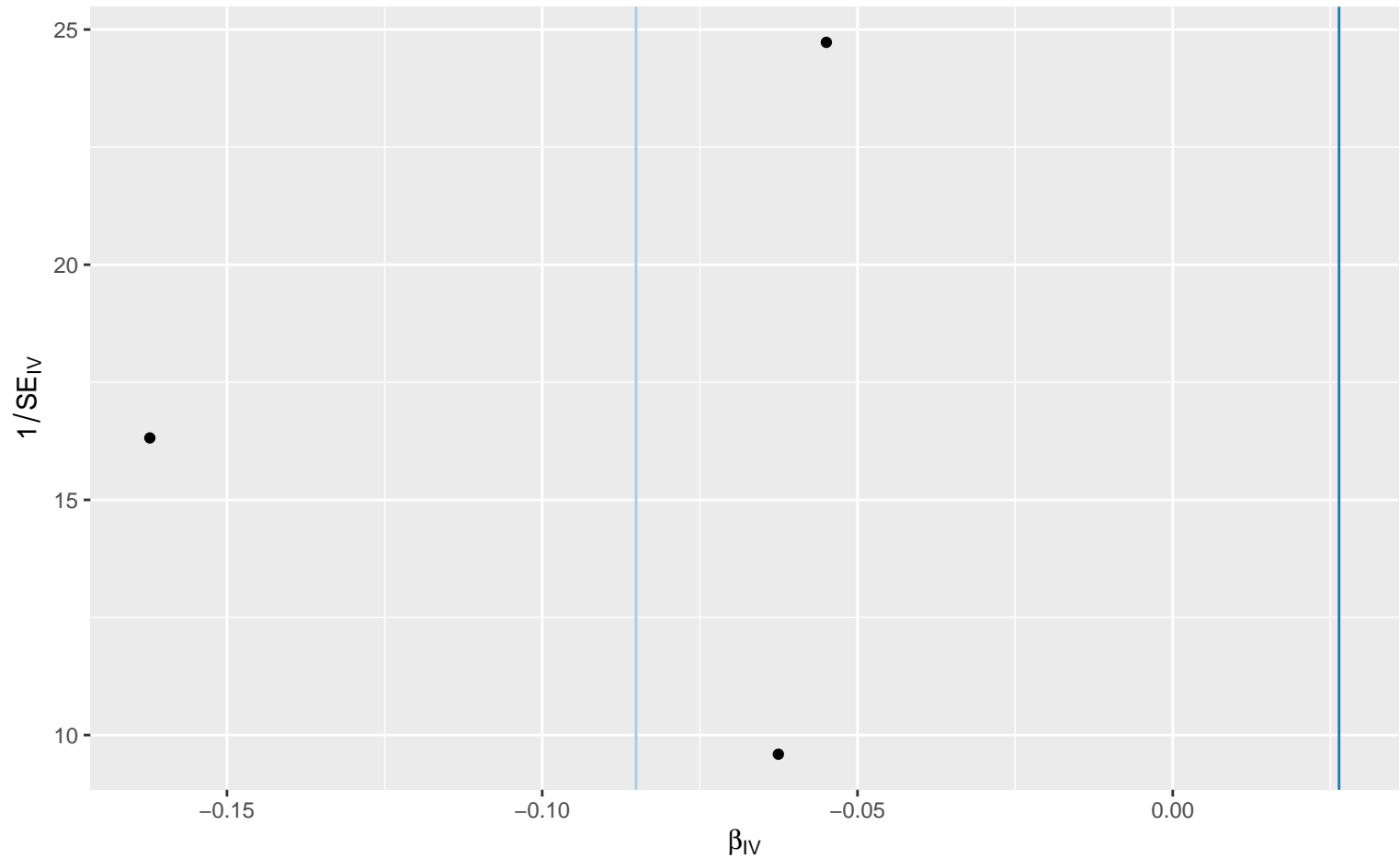

Supplement: Supplementary file 1 [file DataSheet1.ZIP › serum_metabolites/GCST90200152_funnel_plot.pdf]

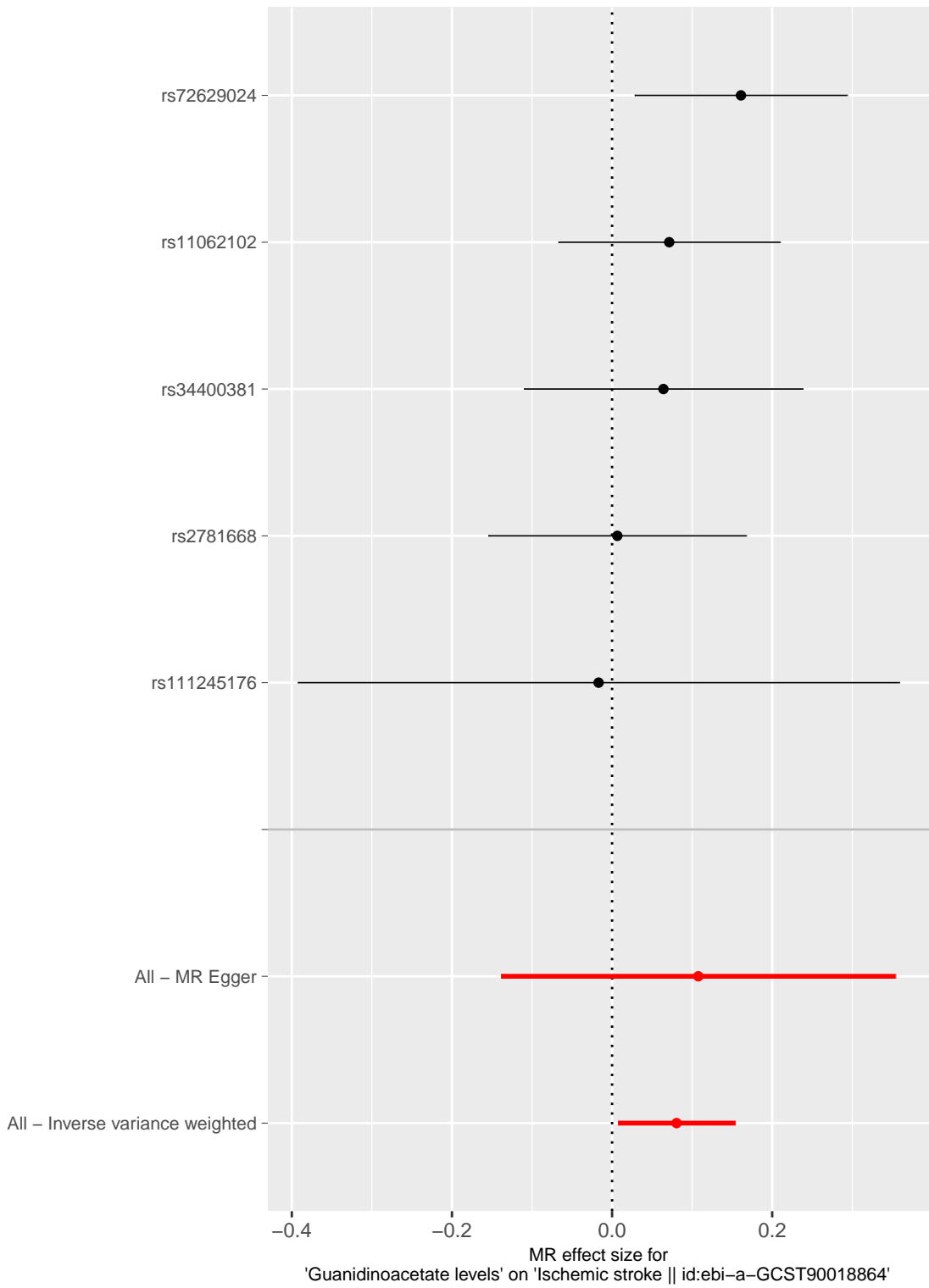

Supplement: Supplementary file 1 [file DataSheet1.ZIP › serum_metabolites/GCST90200384_forest_plot.pdf]

# MR Method

- Inverse variance weighted
- MR Egger

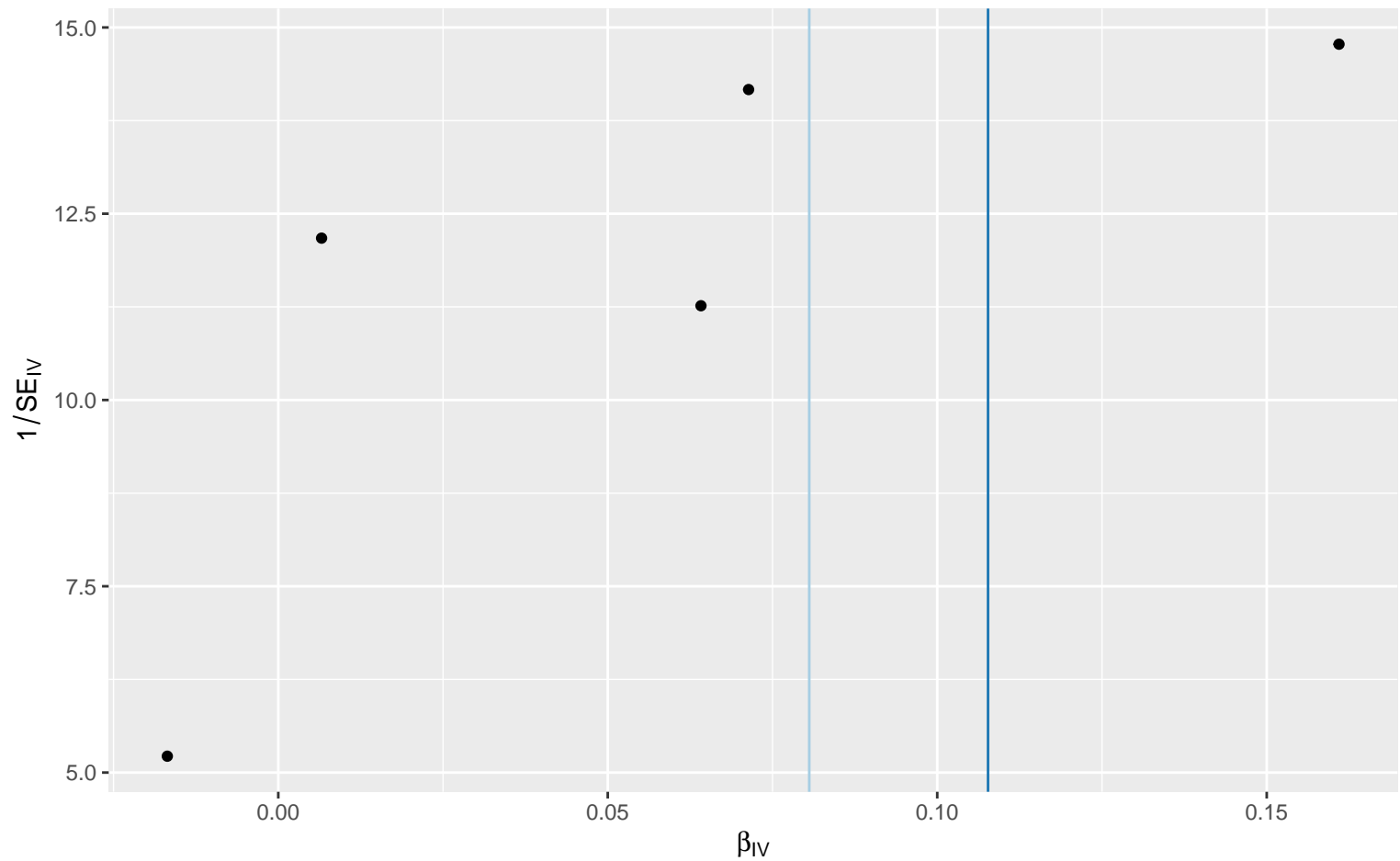

Supplement: Supplementary file 1 [file DataSheet1.ZIP › serum_metabolites/GCST90200384_funnel_plot.pdf]

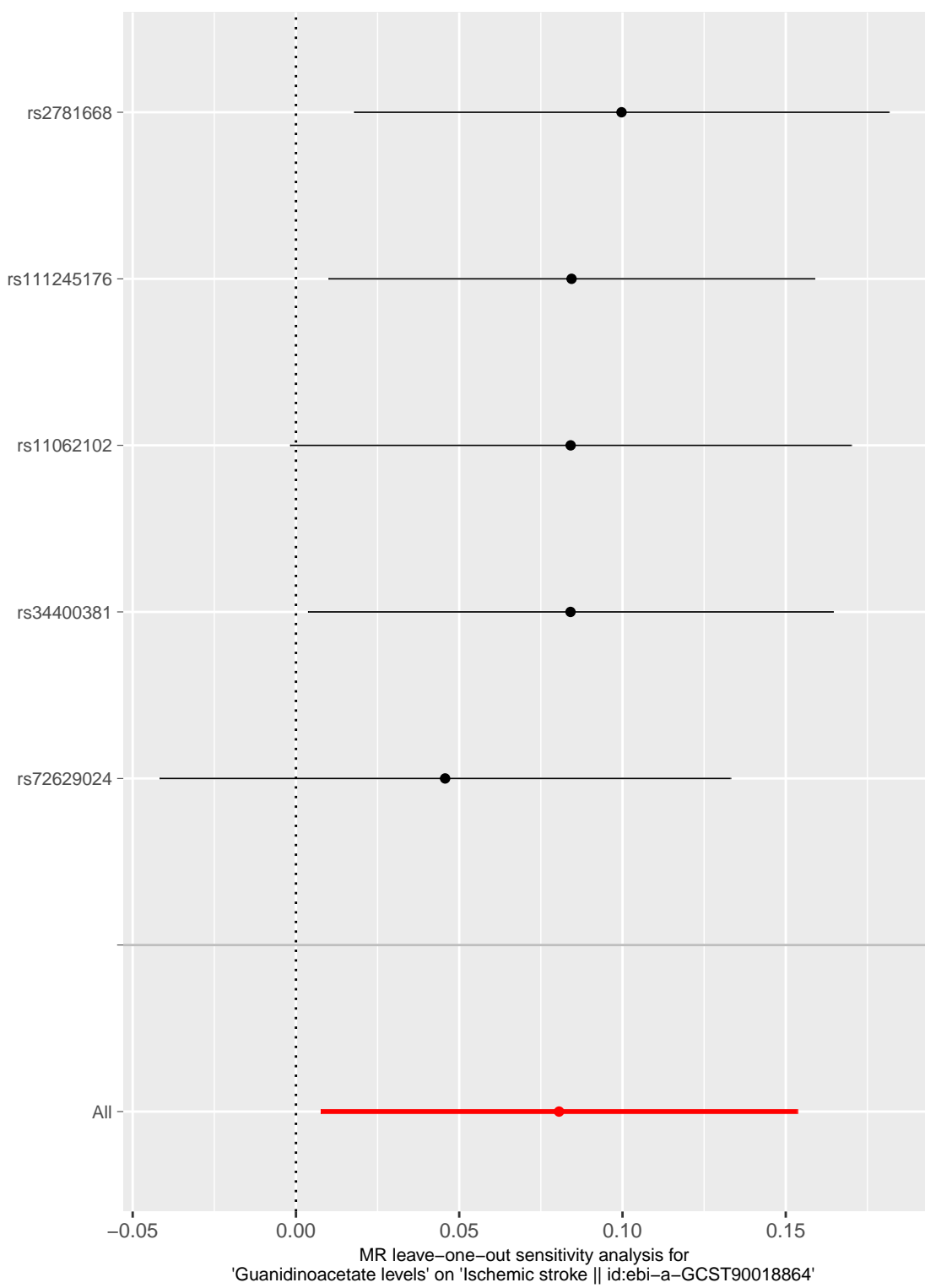

Supplement: Supplementary file 1 [file DataSheet1.ZIP › serum_metabolites/GCST90200384_leaveOneOut_plot.pdf]

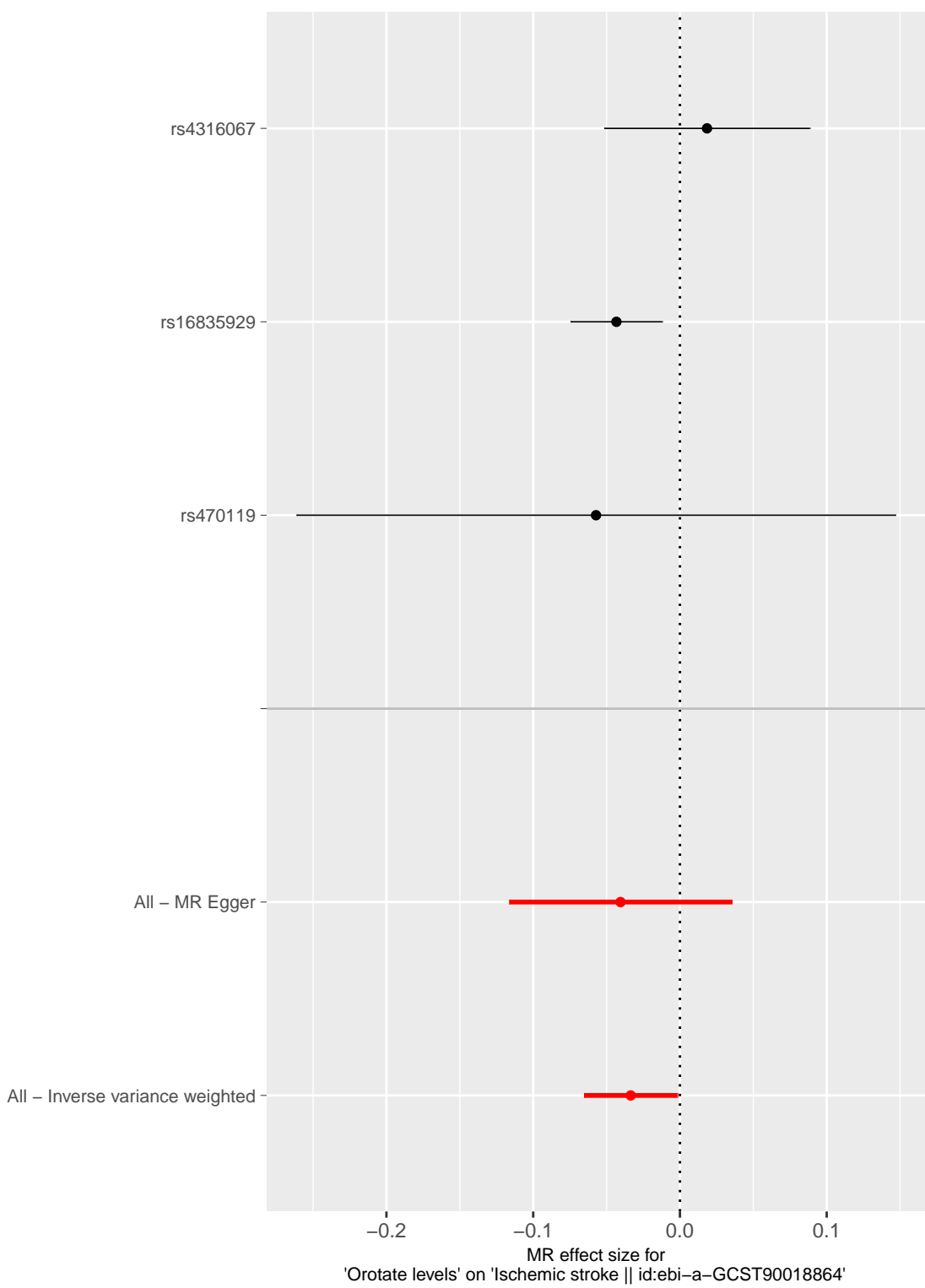

Supplement: Supplementary file 1 [file DataSheet1.ZIP › serum_metabolites/GCST90200392_forest_plot.pdf]

# MR Method

- Inverse variance weighted
- MR Egger

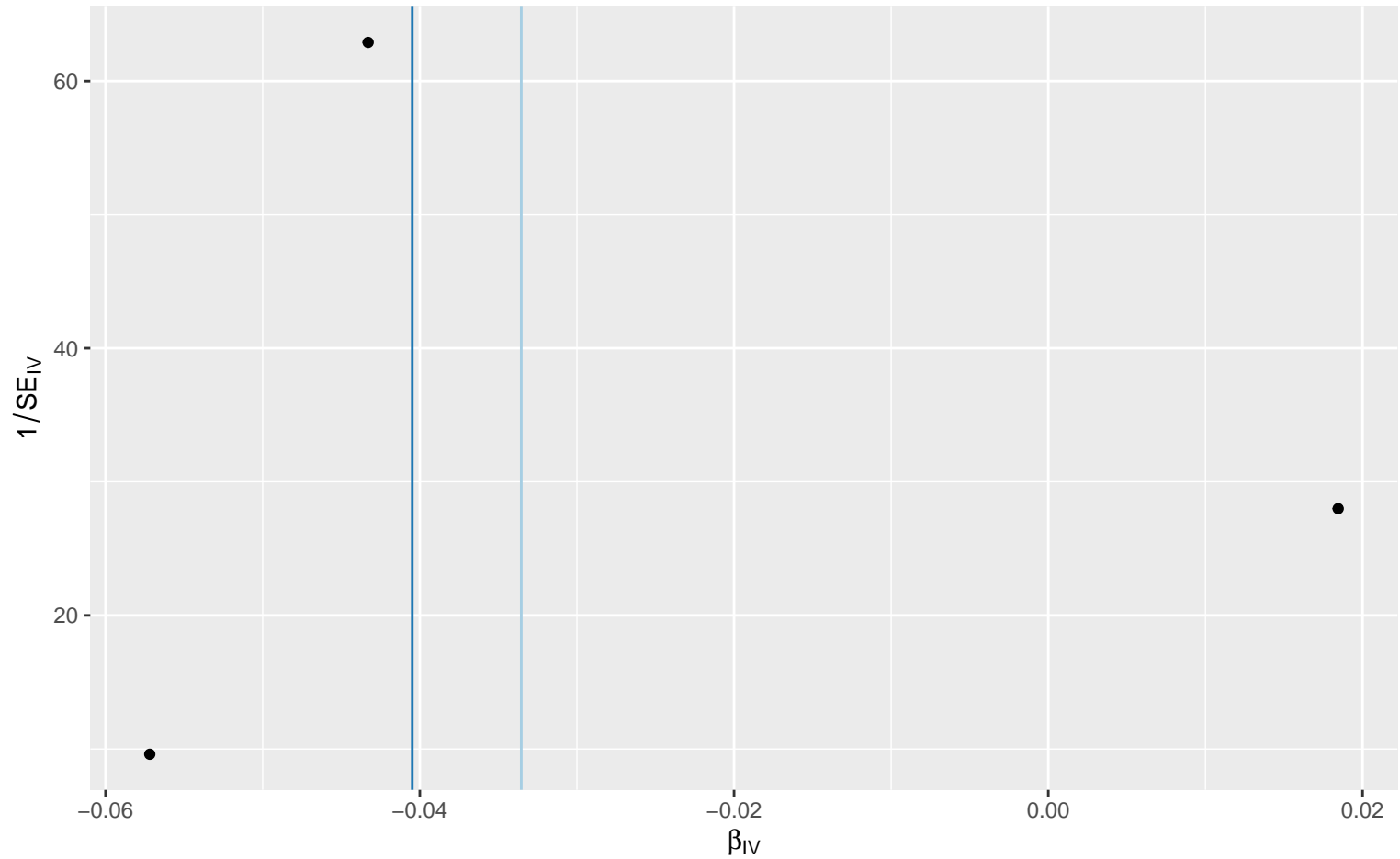

Supplement: Supplementary file 1 [file DataSheet1.ZIP › serum_metabolites/GCST90200392_funnel_plot.pdf]

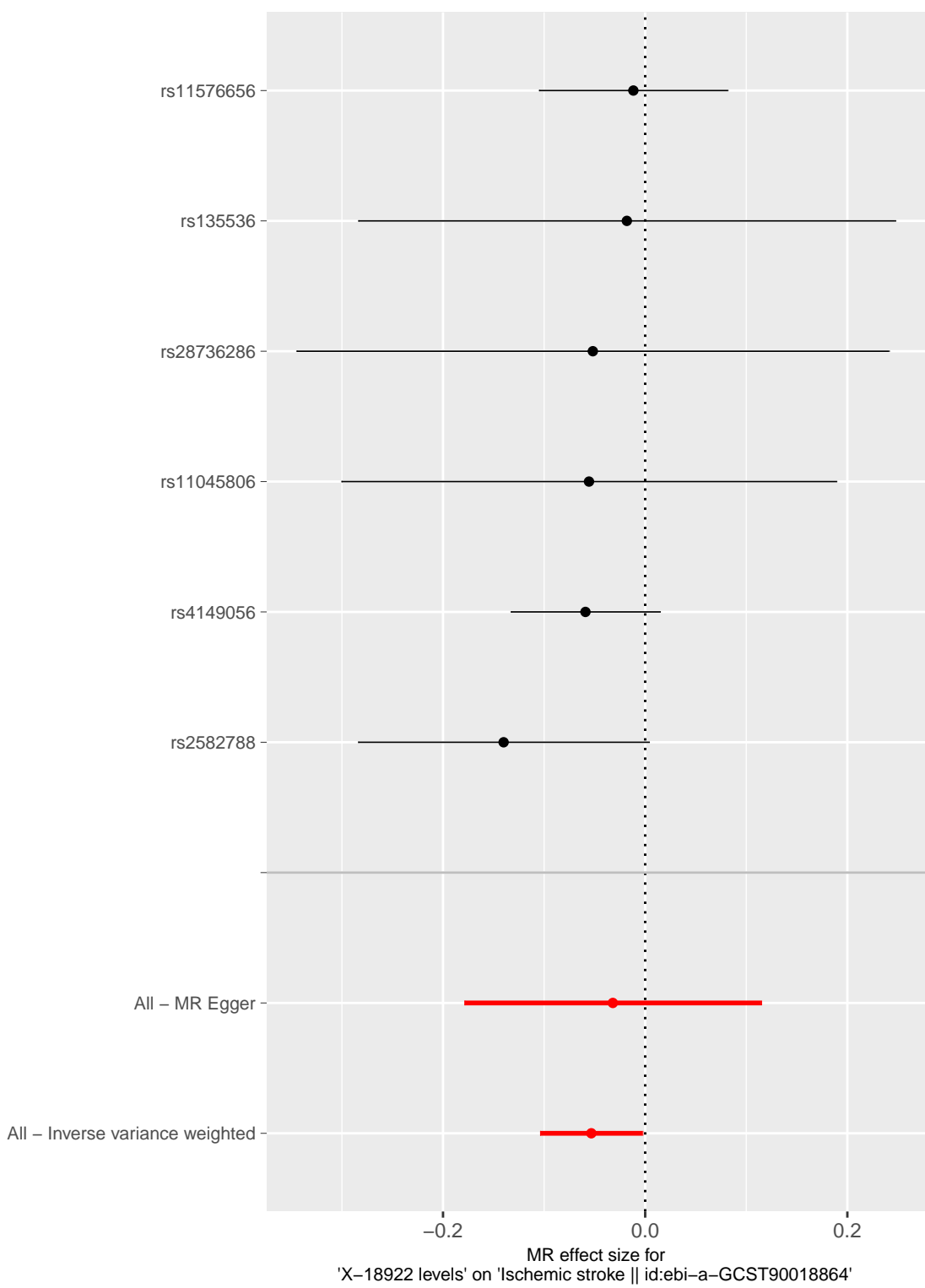

Supplement: Supplementary file 1 [file DataSheet1.ZIP › serum_metabolites/GCST90200578_forest_plot.pdf]

# MR Method

- Inverse variance weighted
- MR Egger

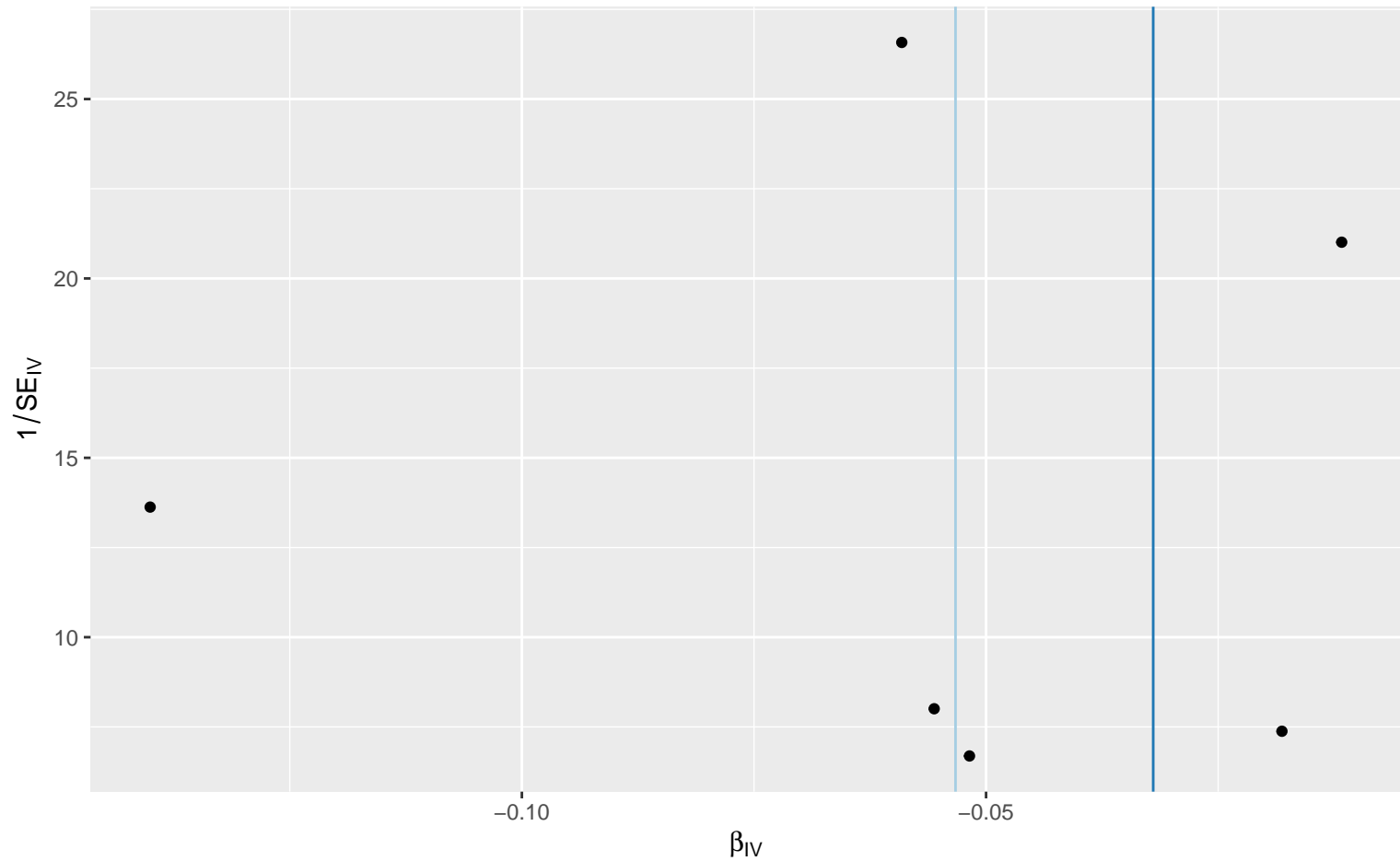

Supplement: Supplementary file 1 [file DataSheet1.ZIP › serum_metabolites/GCST90200578_funnel_plot.pdf]

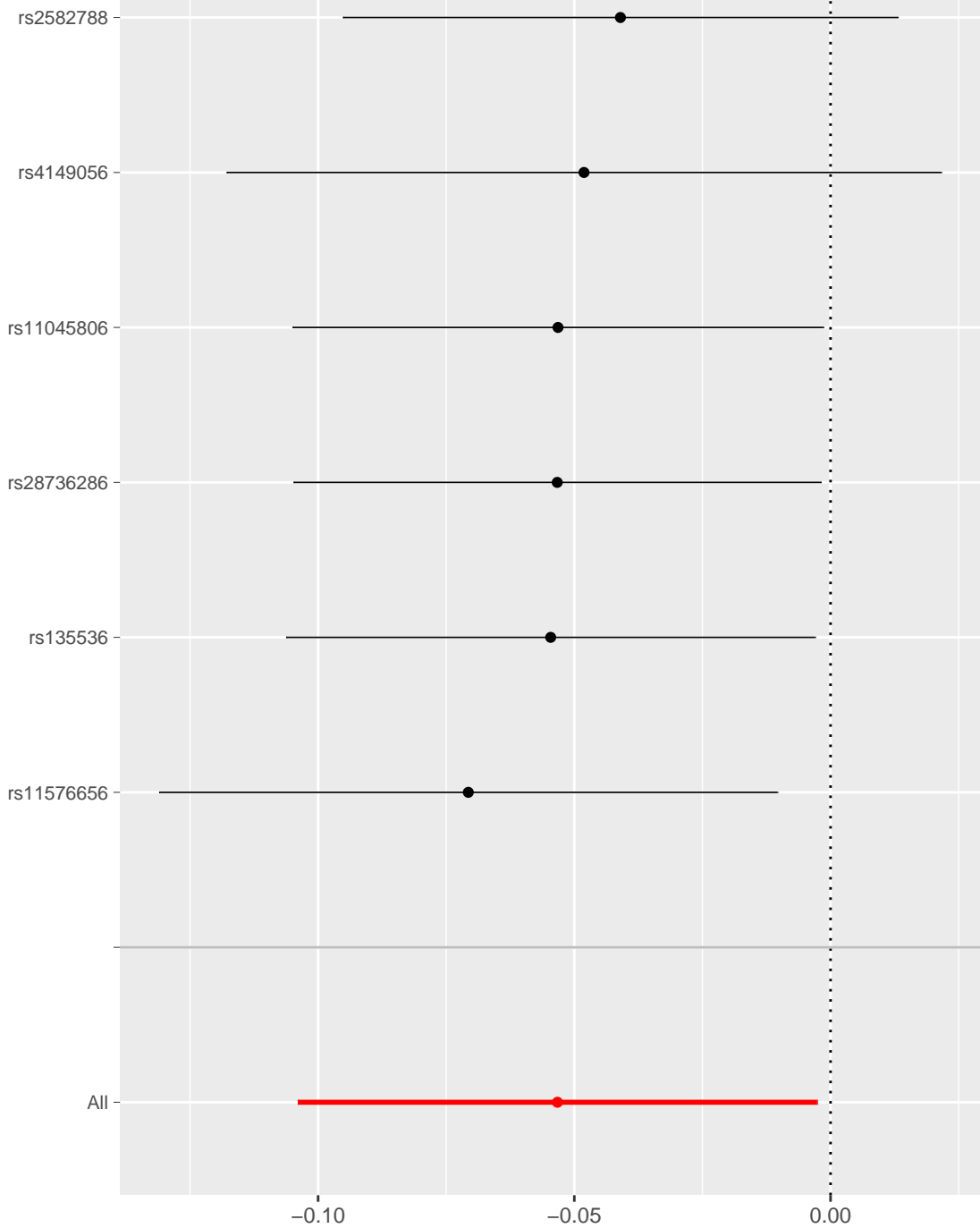

Supplement: Supplementary file 1 [file DataSheet1.ZIP › serum_metabolites/GCST90200578_leaveOneOut_plot.pdf]

SNP effect on Ischemic stroke || id:ebi-a-GCST90018864

### MR Test

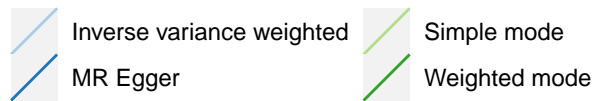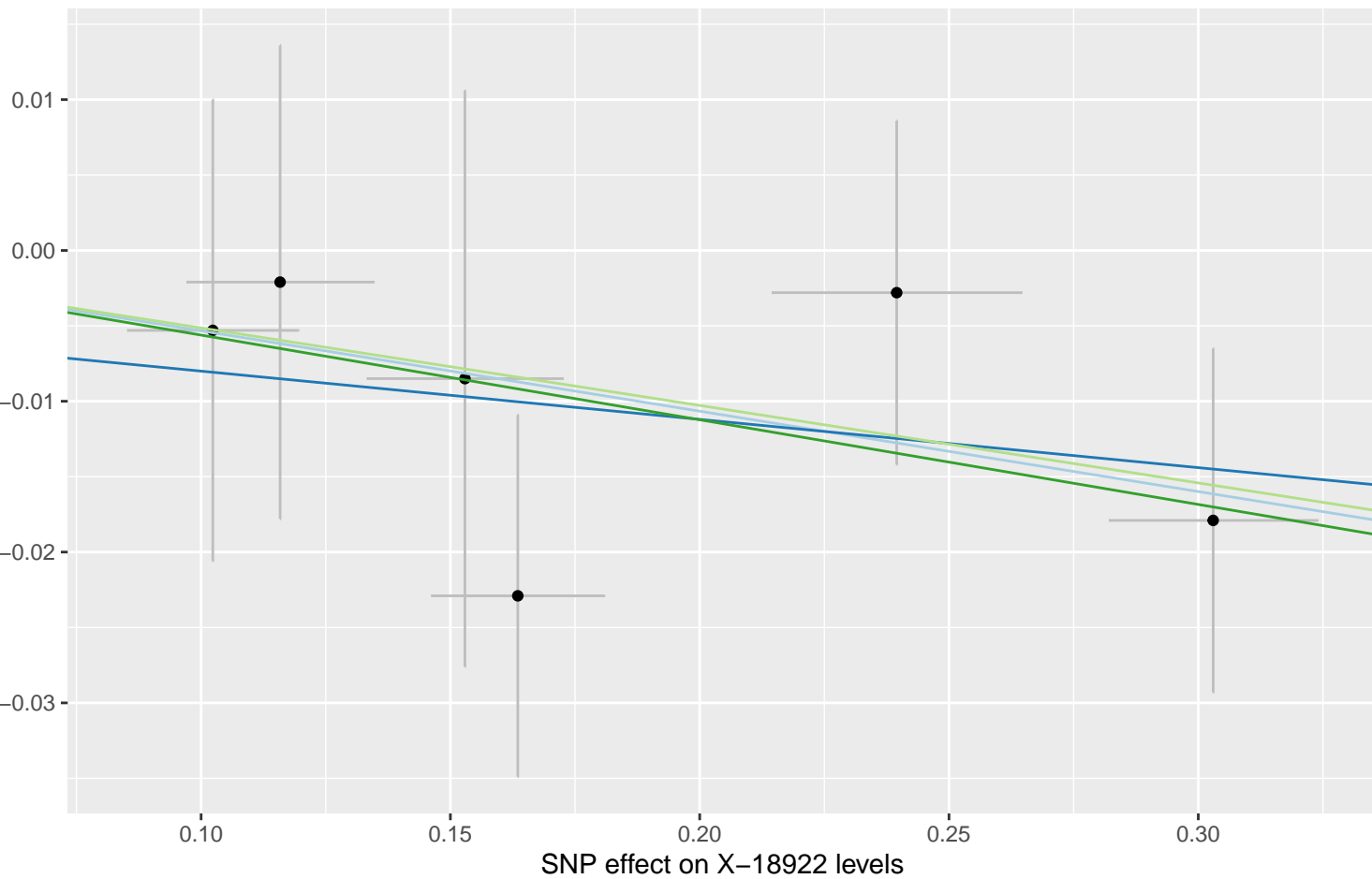

Supplement: Supplementary file 1 [file DataSheet1.ZIP › serum_metabolites/GCST90200578_scatter_plot.pdf]

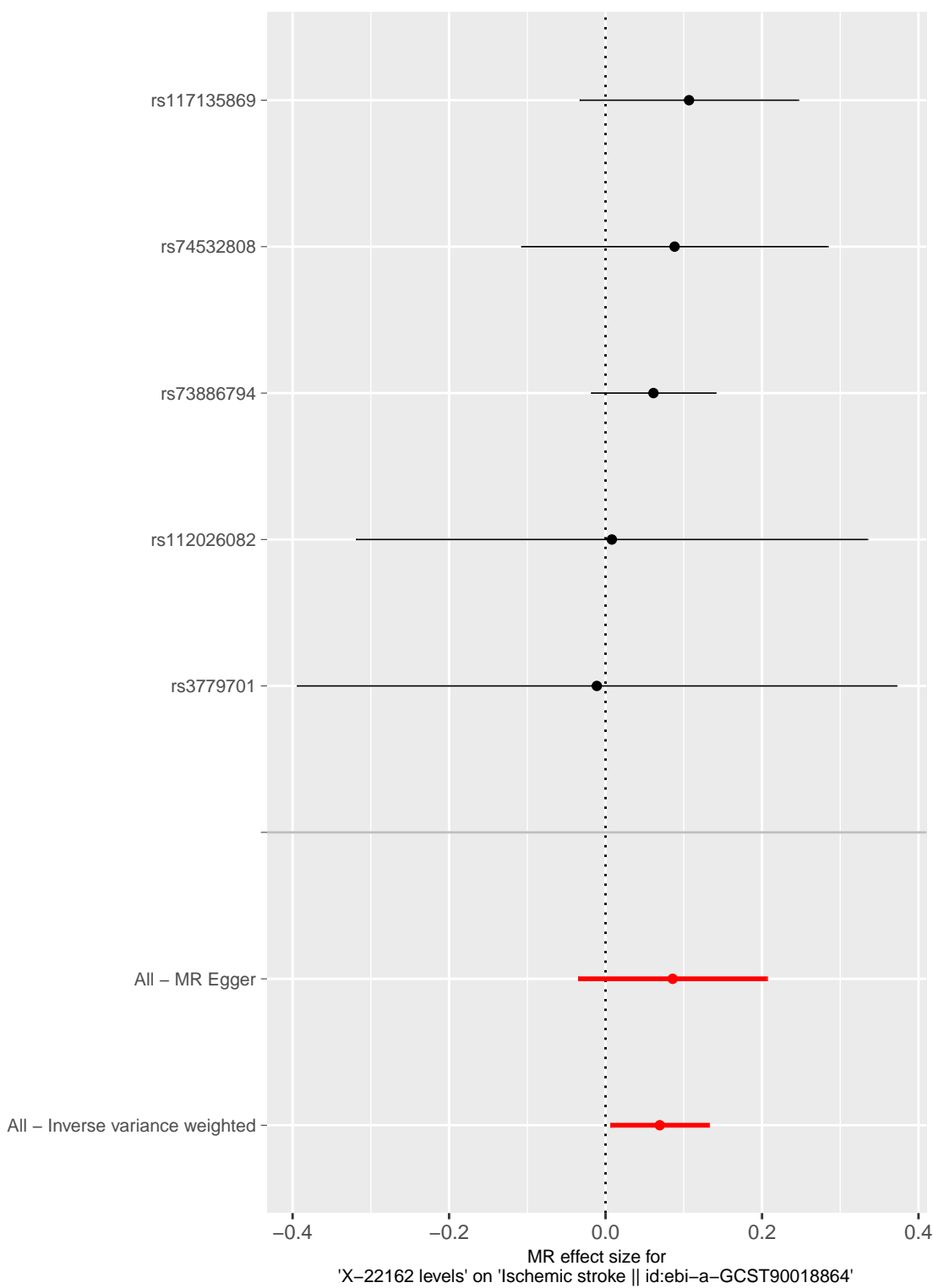

Supplement: Supplementary file 1 [file DataSheet1.ZIP › serum_metabolites/GCST90200608_forest_plot.pdf]

# MR Method

- Inverse variance weighted
- MR Egger

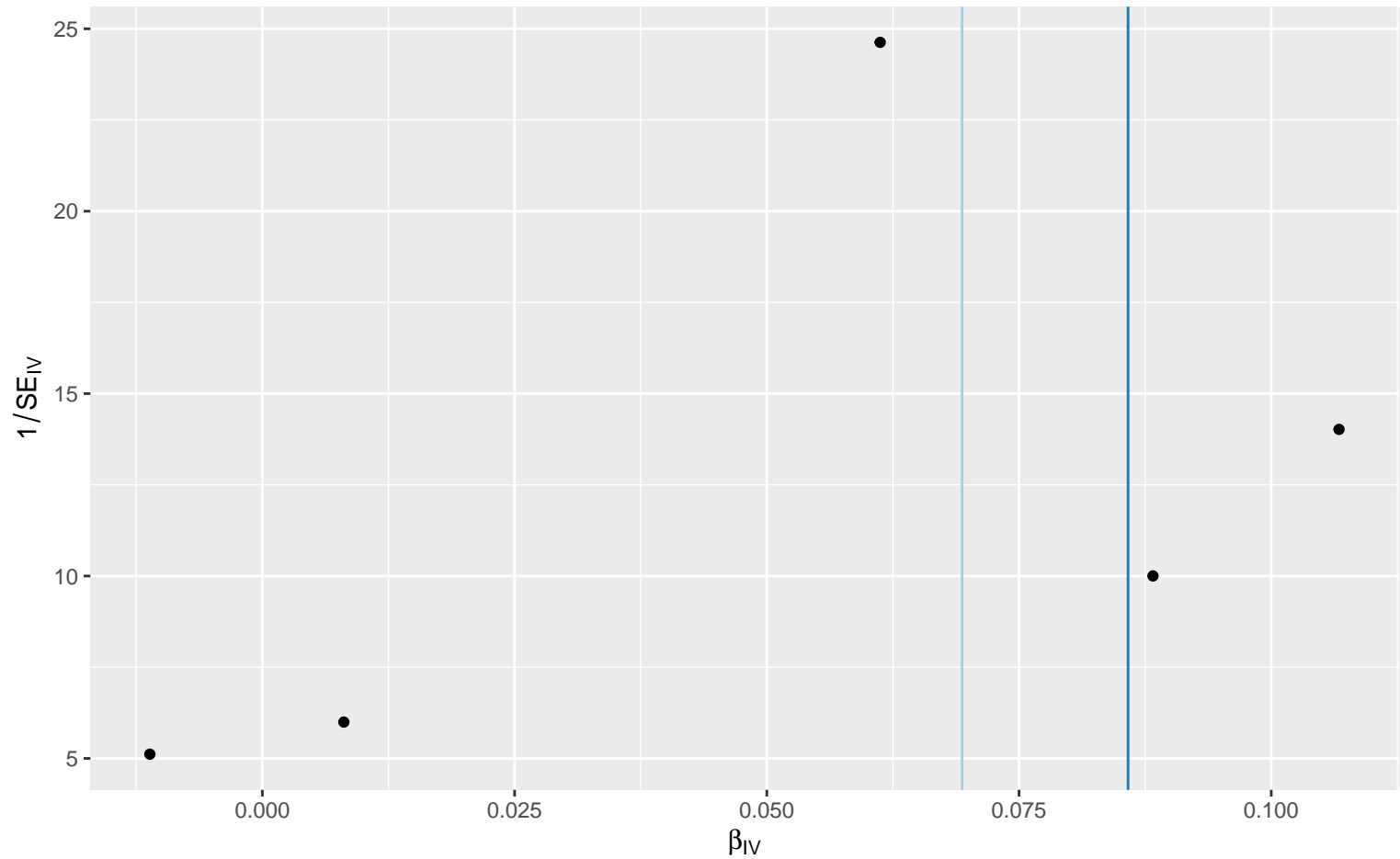

Supplement: Supplementary file 1 [file DataSheet1.ZIP › serum_metabolites/GCST90200608_funnel_plot.pdf]

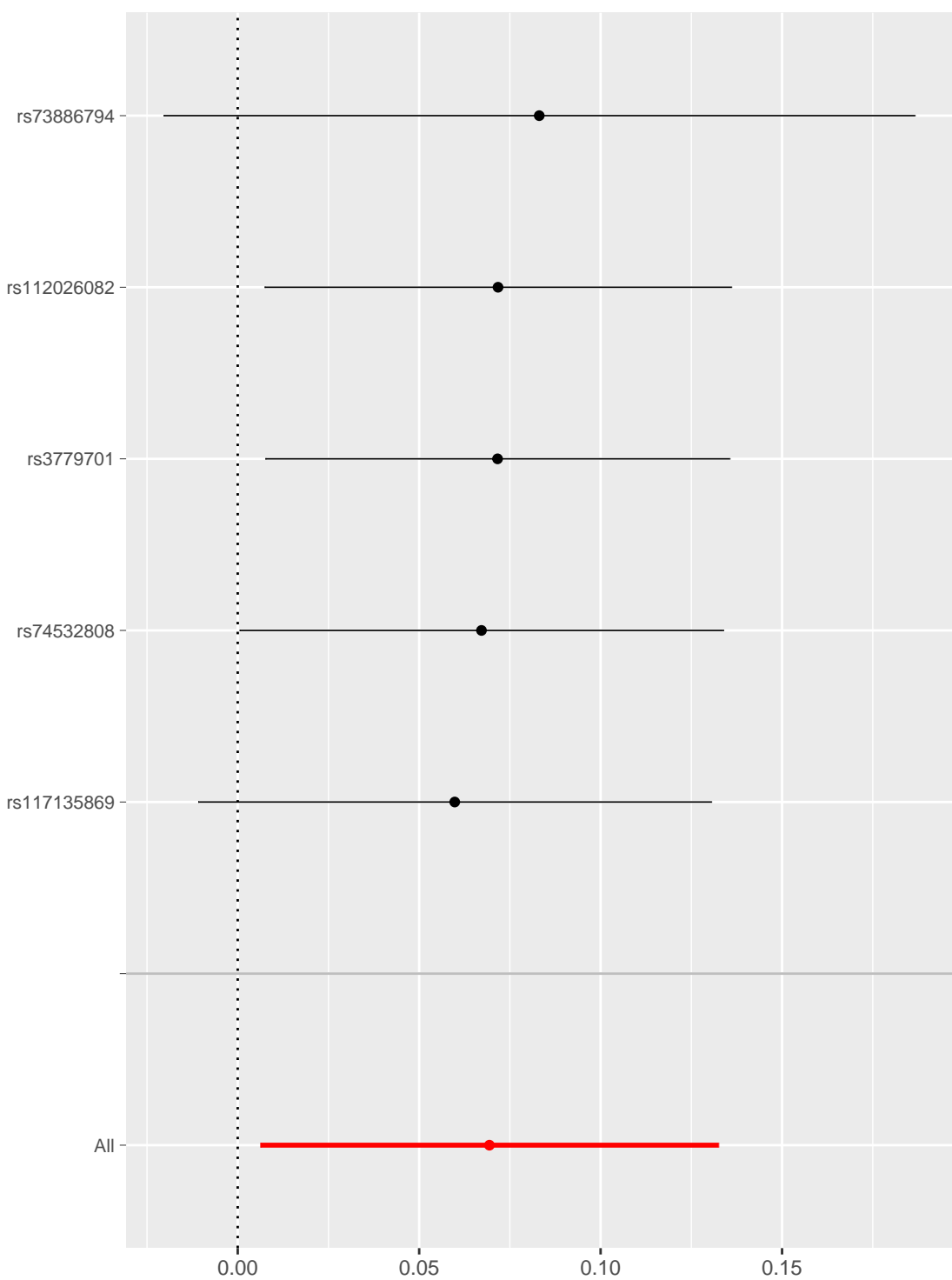

MR leave-one-out sensitivity analysis for  
'X-22162 levels' on 'Ischemic stroke || id:ebi-a-GCST90018864'

Supplement: Supplementary file 1 [file DataSheet1.ZIP › serum_metabolites/GCST90200608_leaveOneOut_plot.pdf]

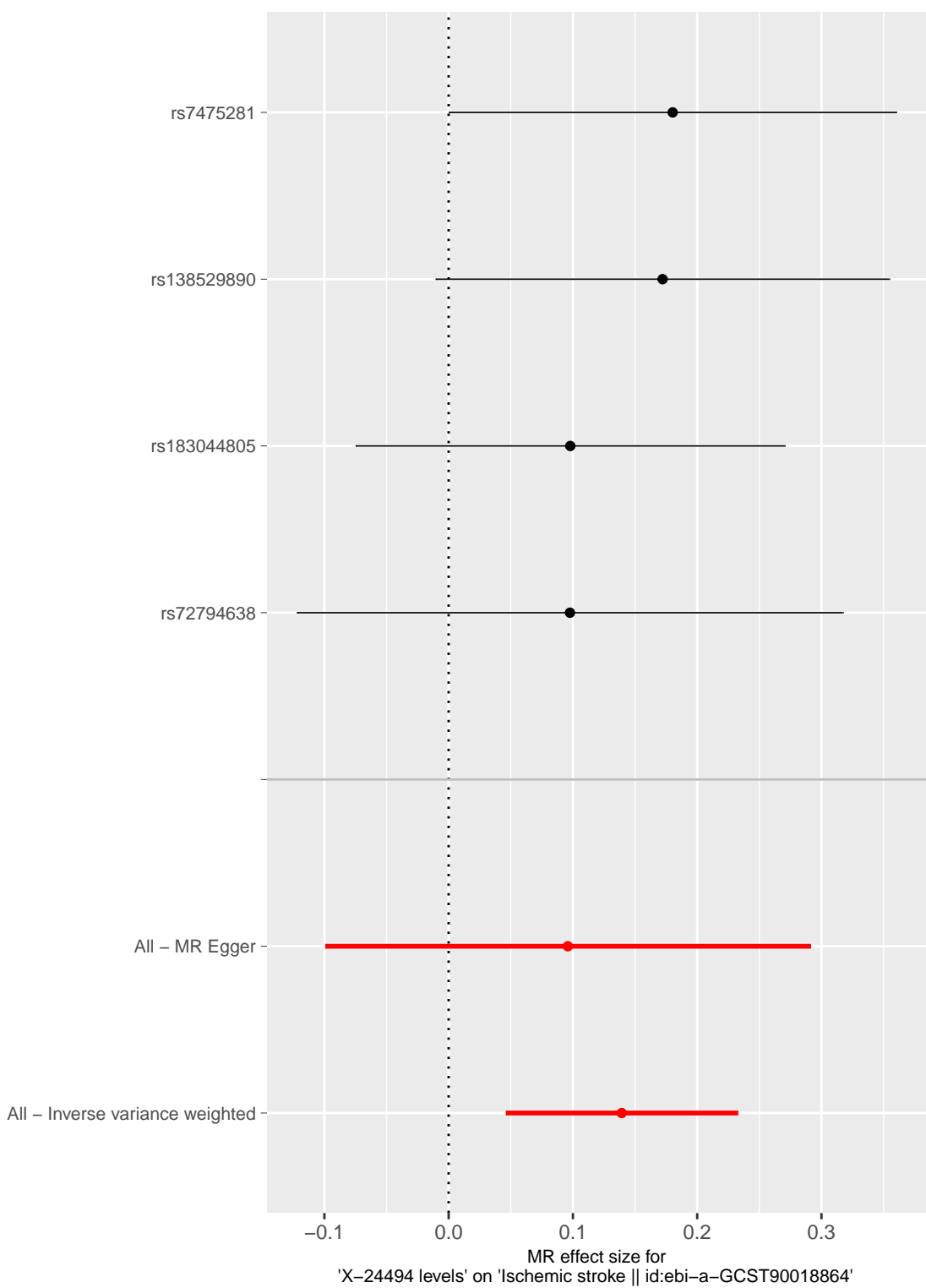

Supplement: Supplementary file 1 [file DataSheet1.ZIP › serum_metabolites/GCST90200635_forest_plot.pdf]

# MR Method

- Inverse variance weighted
- MR Egger

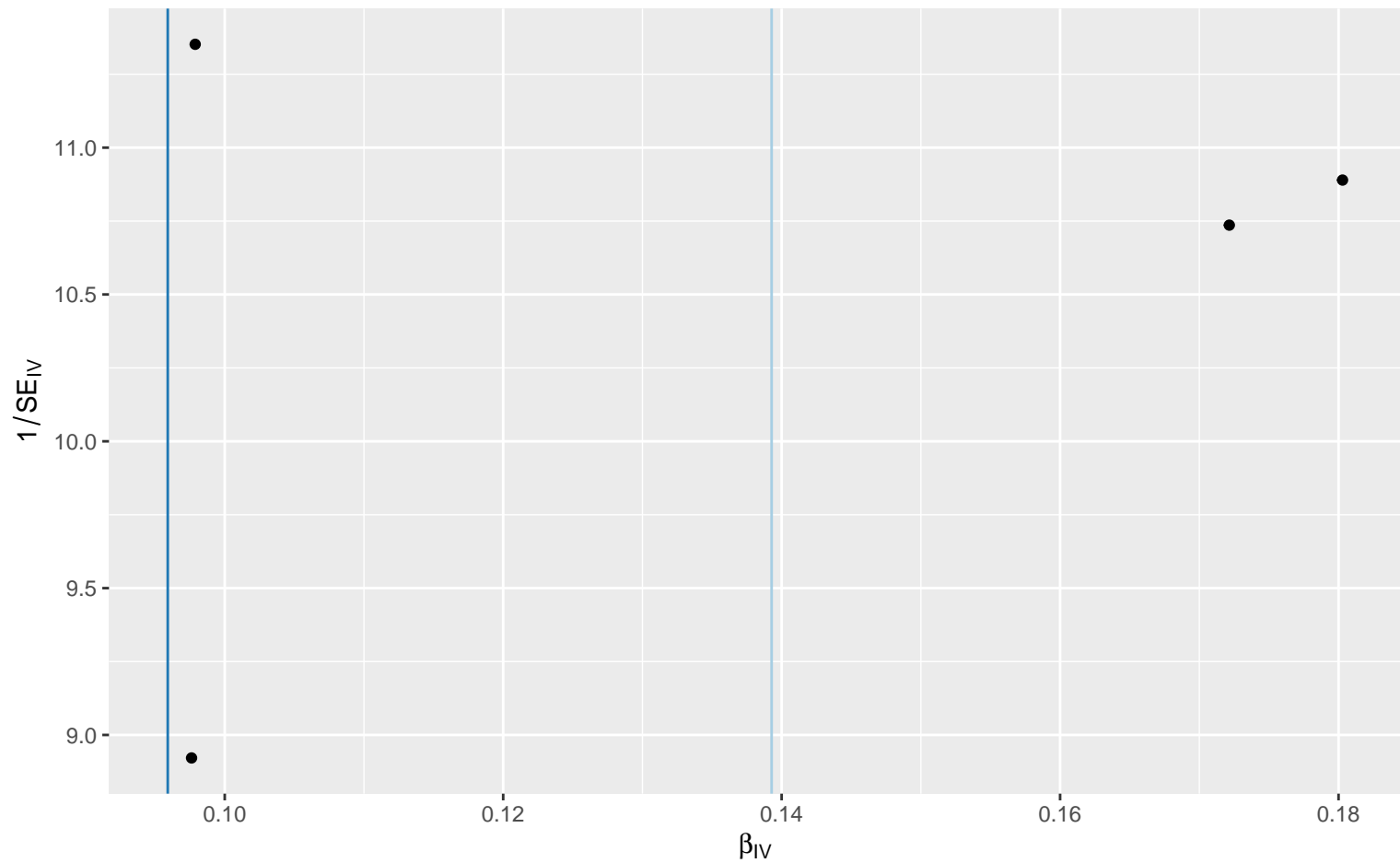

Supplement: Supplementary file 1 [file DataSheet1.ZIP › serum_metabolites/GCST90200635_funnel_plot.pdf]

rs183044805

rs72794638

rs138529890

rs7475281

All

0.0

0.1

0.2

MR leave-one-out sensitivity analysis for  
'X-24494 levels' on 'Ischemic stroke || id:ebi-a-GCST90018864'

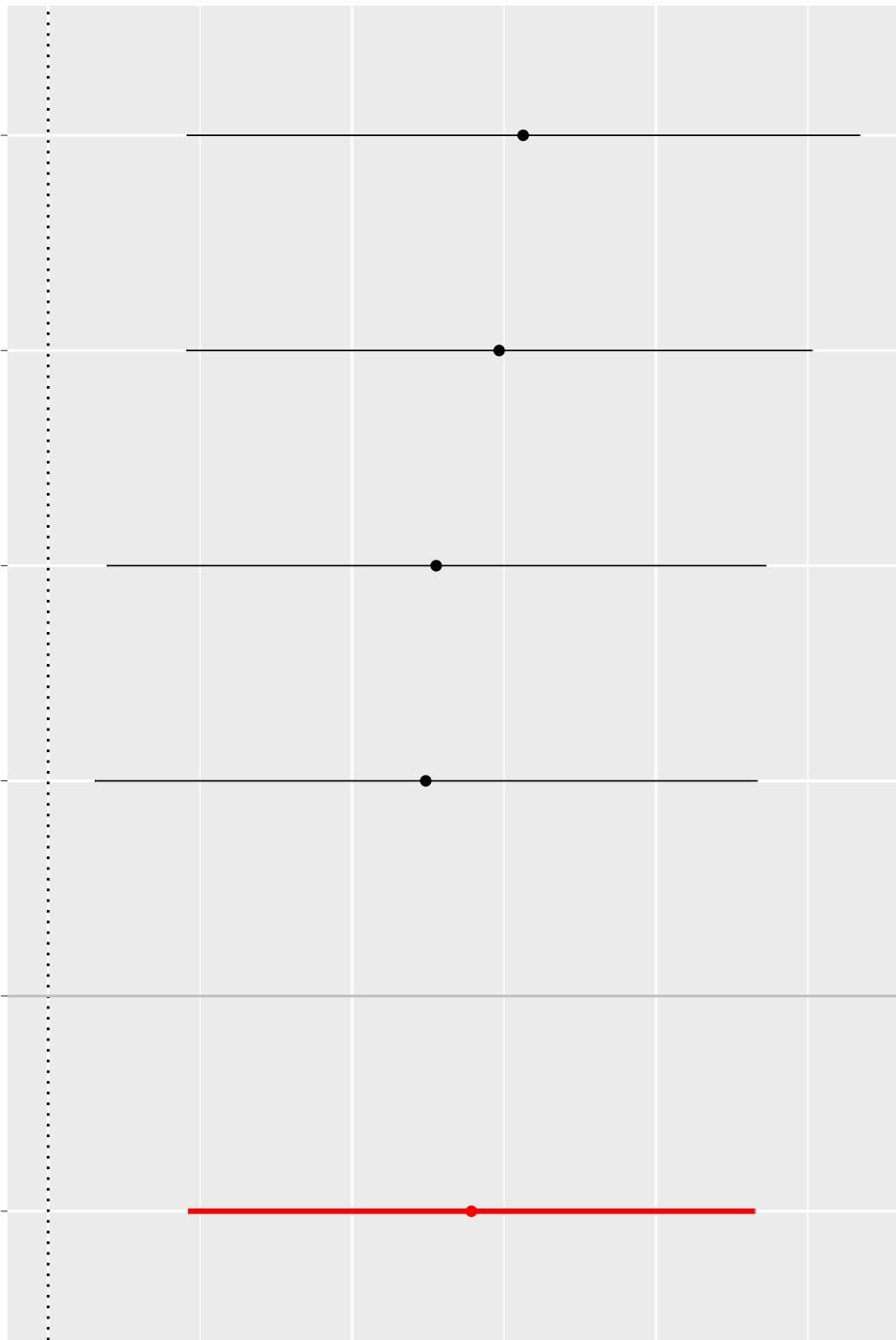

Supplement: Supplementary file 1 [file DataSheet1.ZIP › serum_metabolites/GCST90200635_leaveOneOut_plot.pdf]

# MR Test

- Inverse variance weighted
- MR Egger
- Simple mode
- Weighted mode

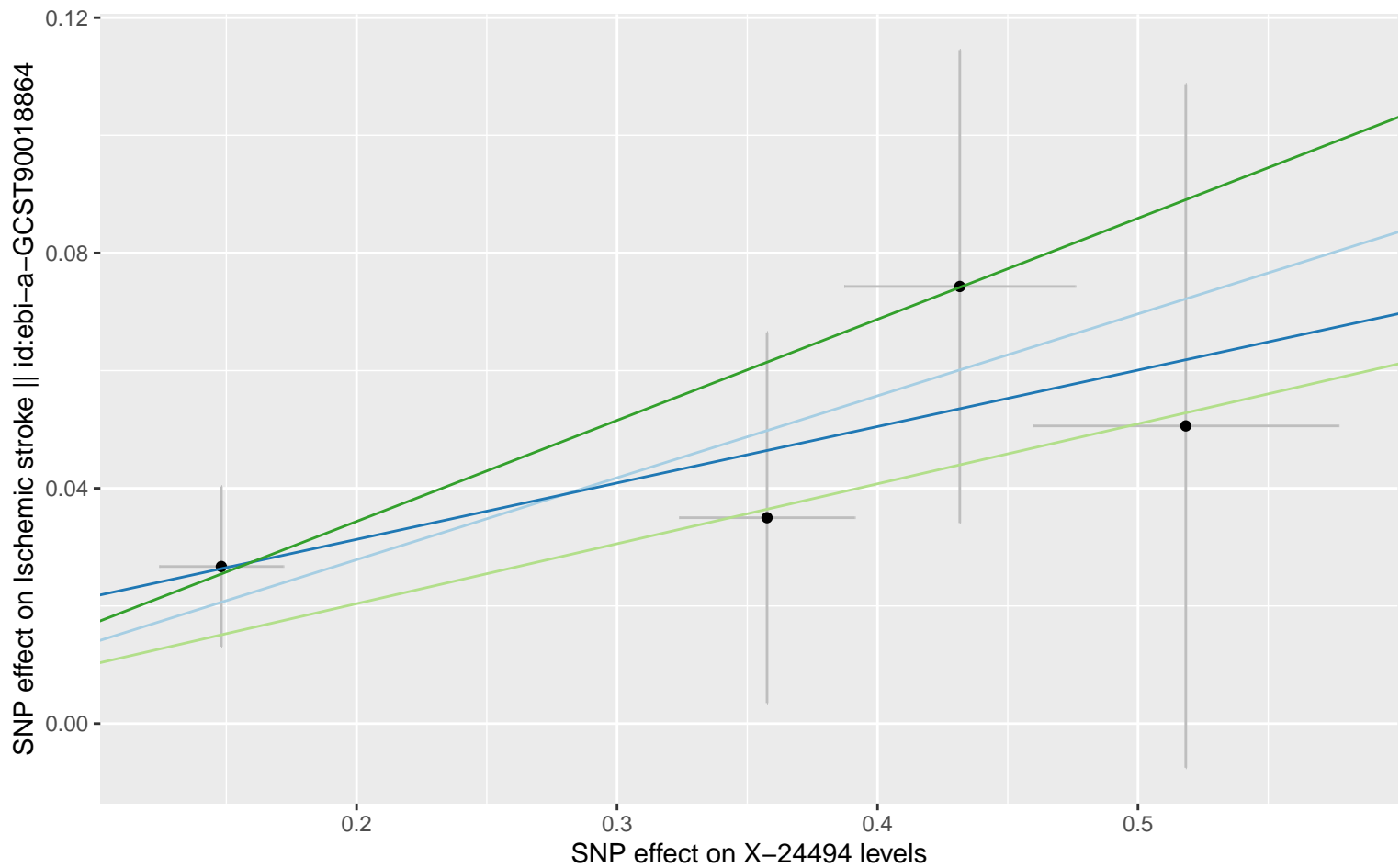

Supplement: Supplementary file 1 [file DataSheet1.ZIP › serum_metabolites/GCST90200635_scatter_plot.pdf]

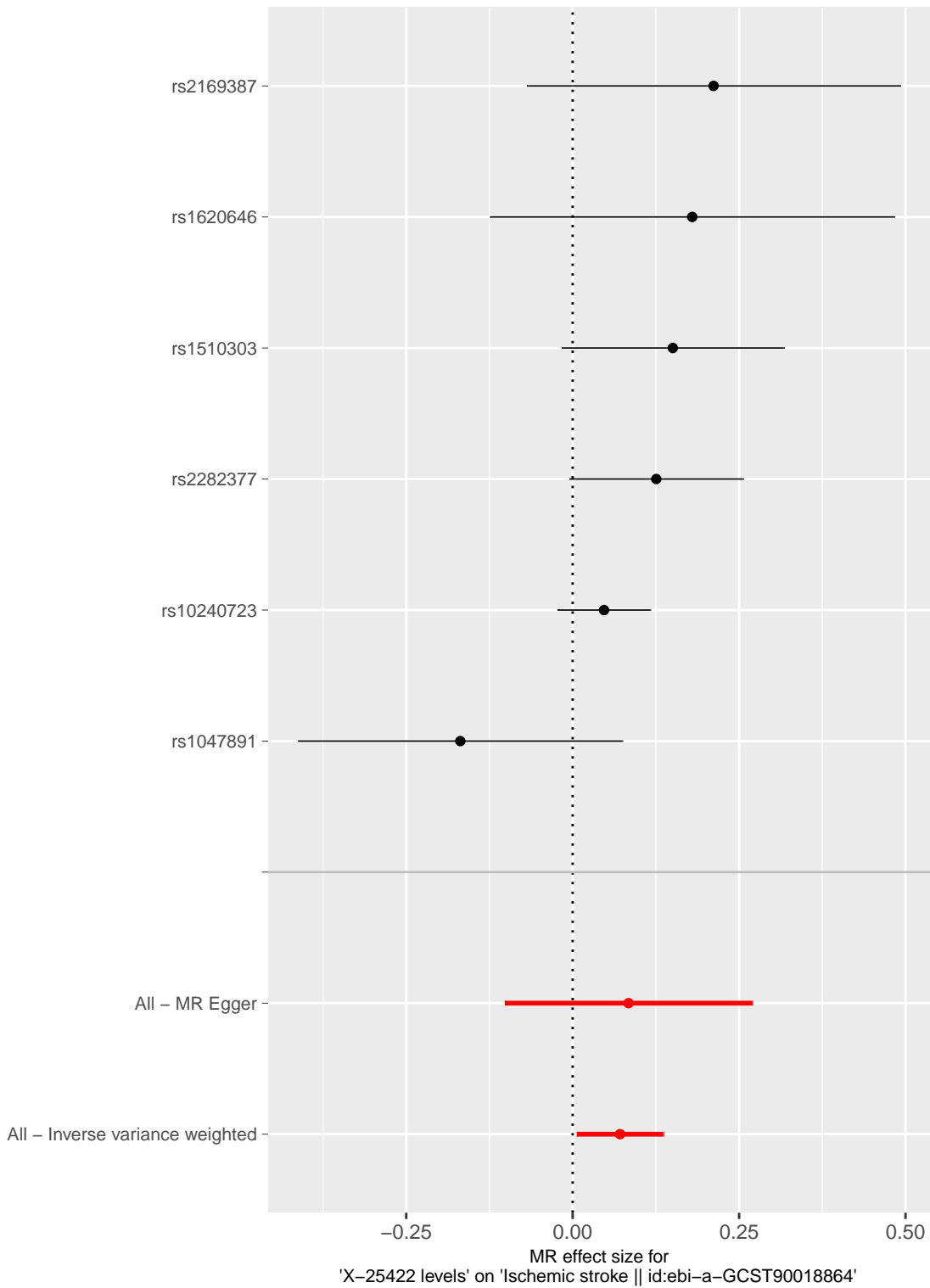

Supplement: Supplementary file 1 [file DataSheet1.ZIP › serum_metabolites/GCST90200661_forest_plot.pdf]

# MR Method

- Inverse variance weighted
- MR Egger

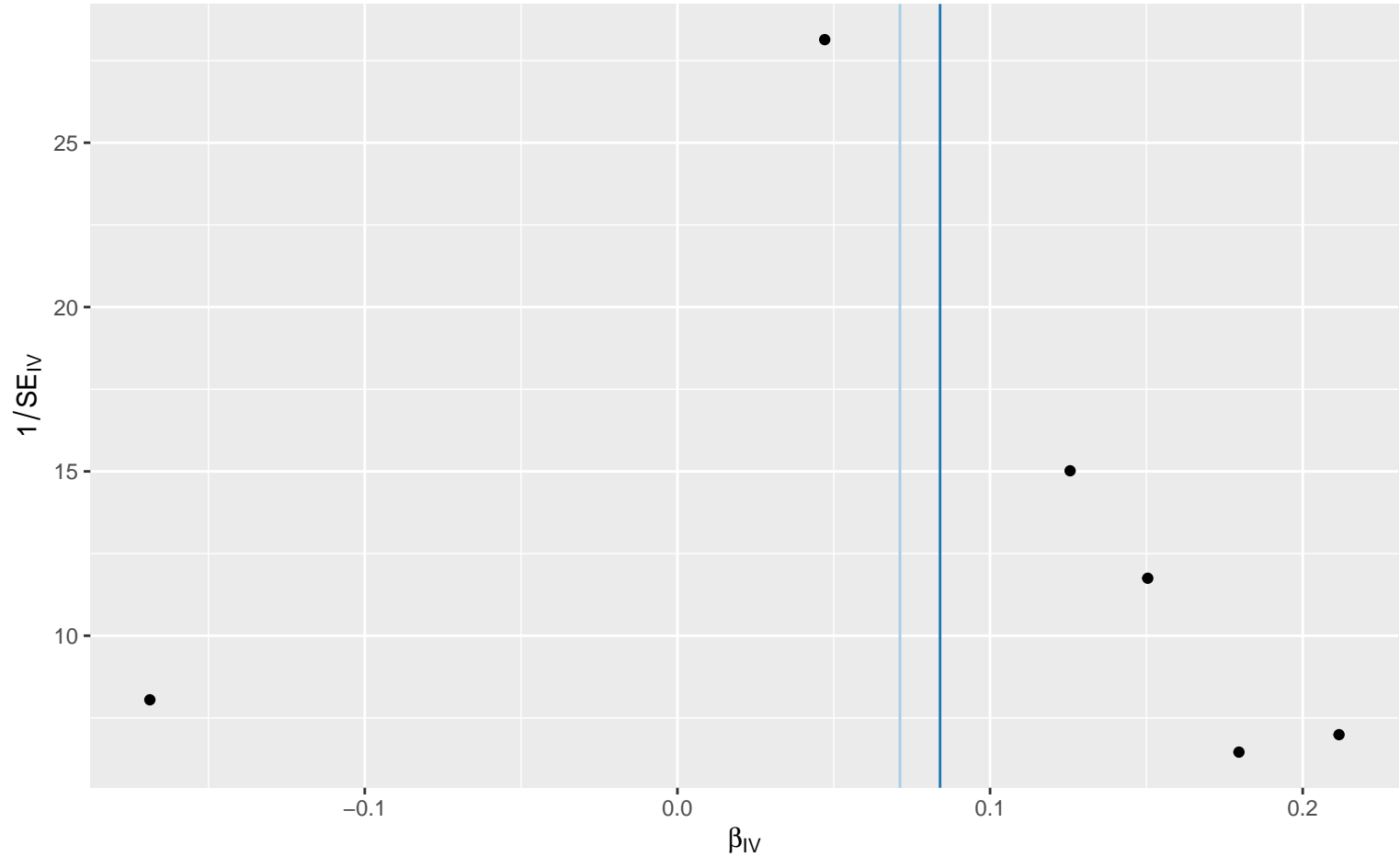

Supplement: Supplementary file 1 [file DataSheet1.ZIP › serum_metabolites/GCST90200661_funnel_plot.pdf]

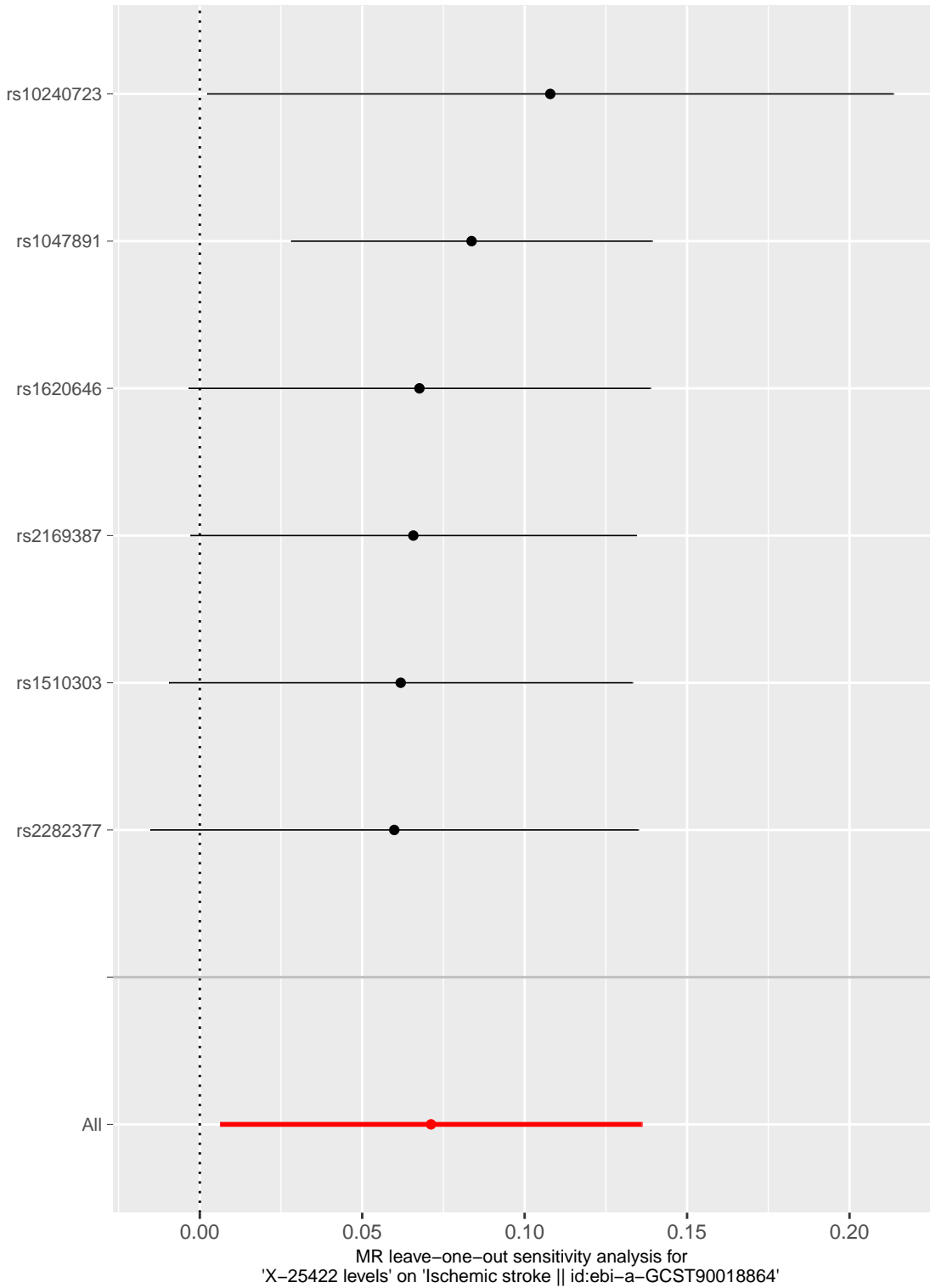

Supplement: Supplementary file 1 [file DataSheet1.ZIP › serum_metabolites/GCST90200661_leaveOneOut_plot.pdf]

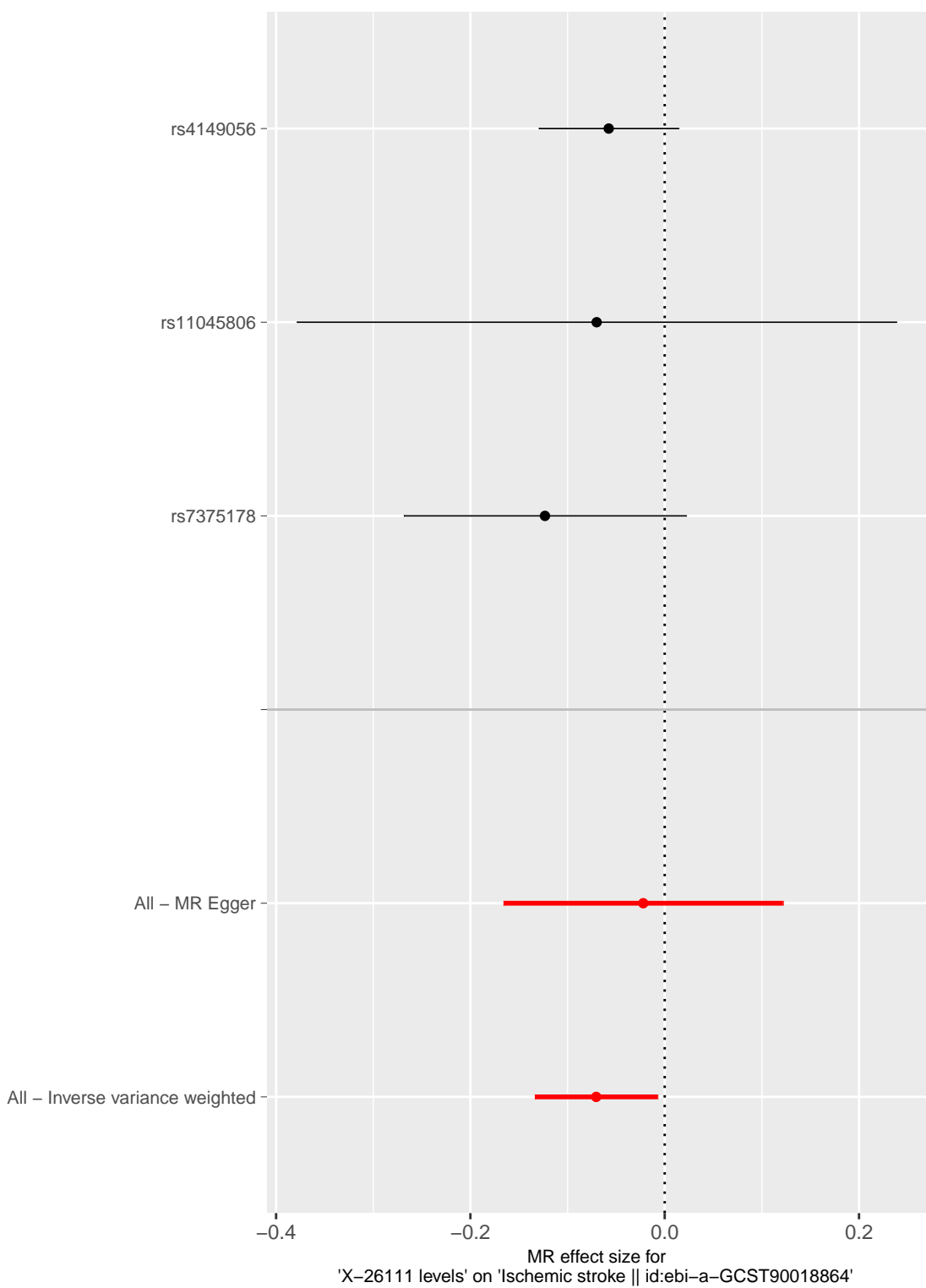

Supplement: Supplementary file 1 [file DataSheet1.ZIP › serum_metabolites/GCST90200670_forest_plot.pdf]

# MR Method

- Inverse variance weighted
- MR Egger

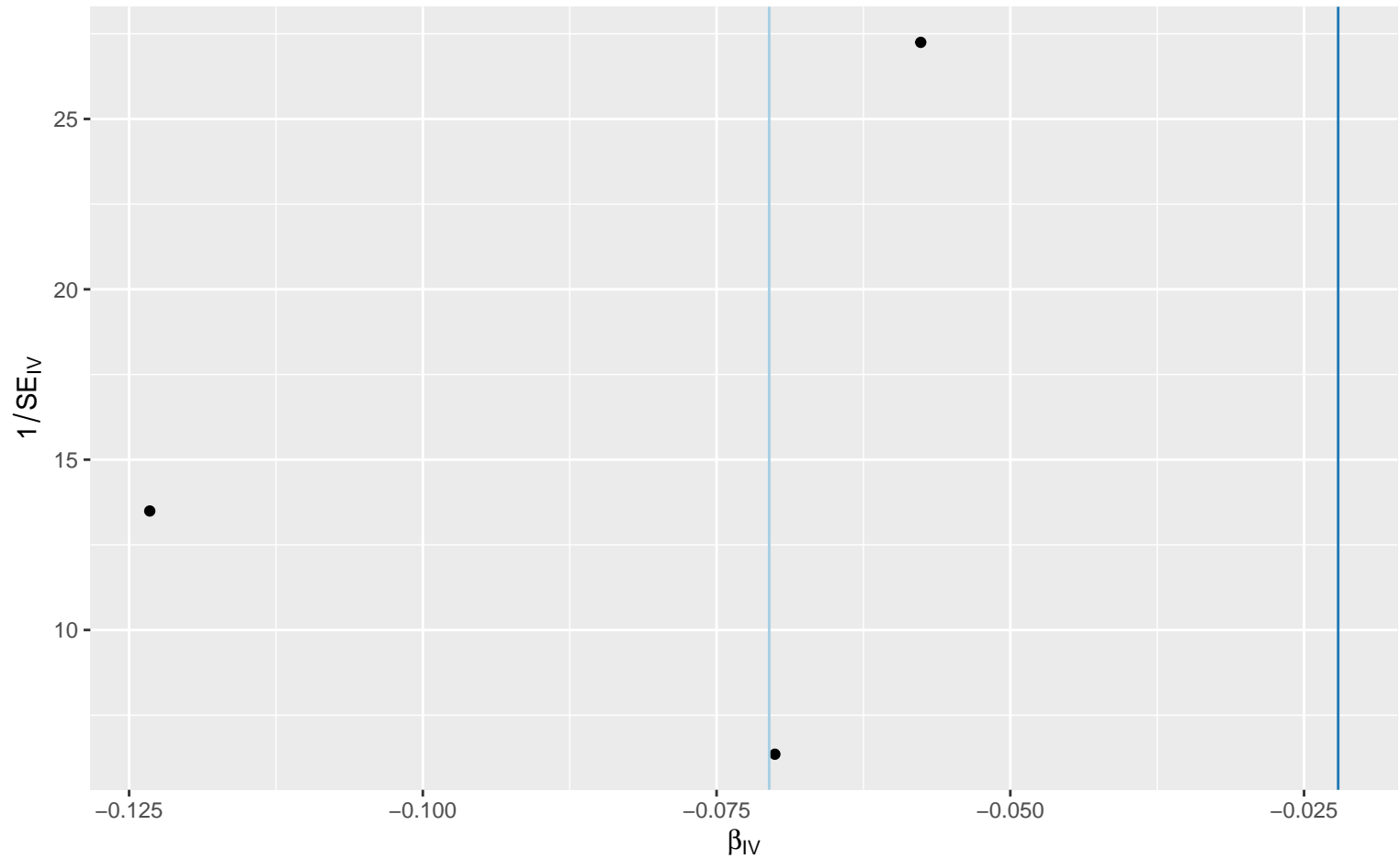

Supplement: Supplementary file 1 [file DataSheet1.ZIP › serum_metabolites/GCST90200670_funnel_plot.pdf]

rs7375178

rs11045806

rs4149056

All

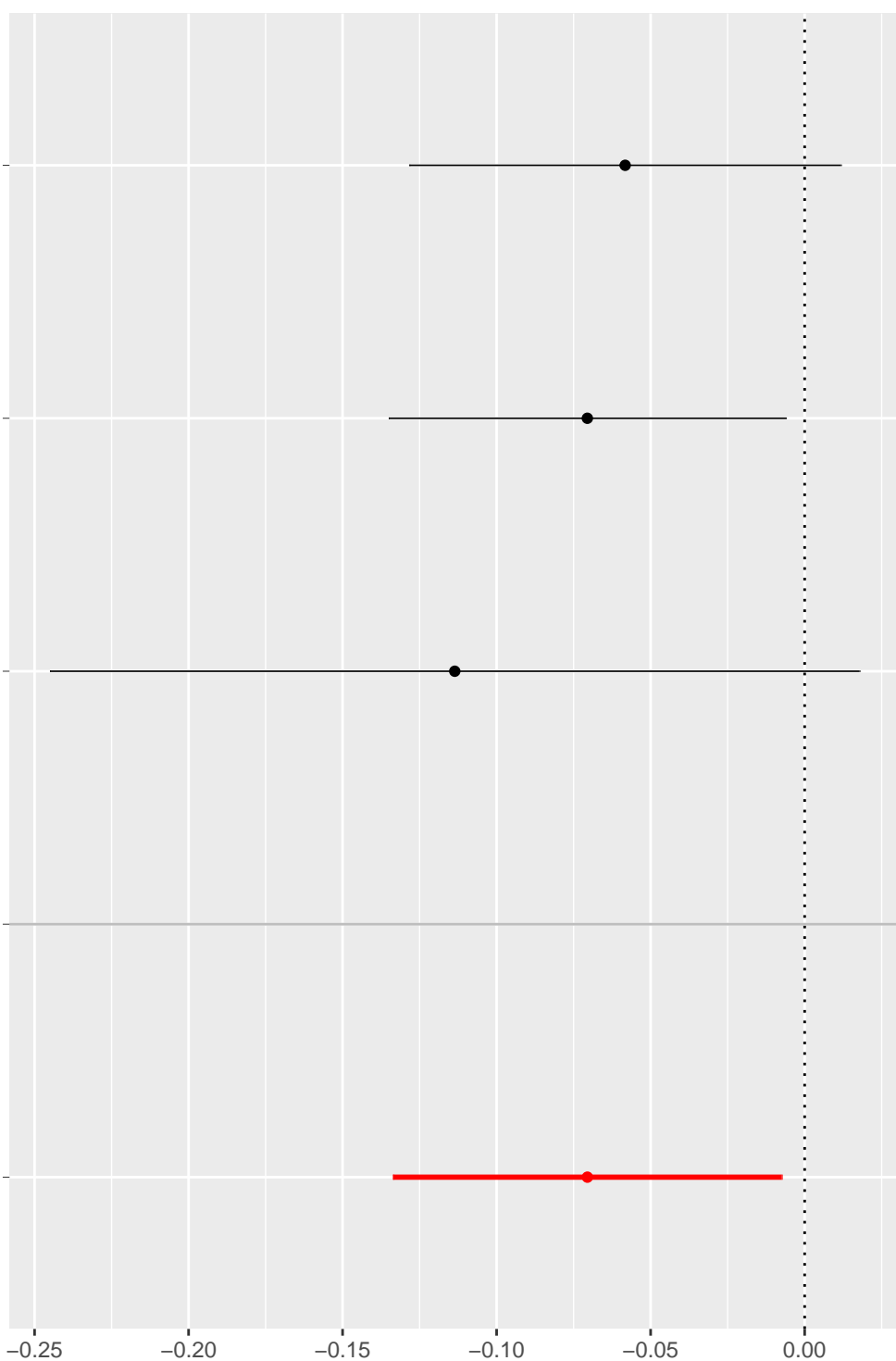

MR leave-one-out sensitivity analysis for  
'X-26111 levels' on 'Ischemic stroke || id:ebi-a-GCST90018864'

Supplement: Supplementary file 1 [file DataSheet1.ZIP › serum_metabolites/GCST90200670_leaveOneOut_plot.pdf]

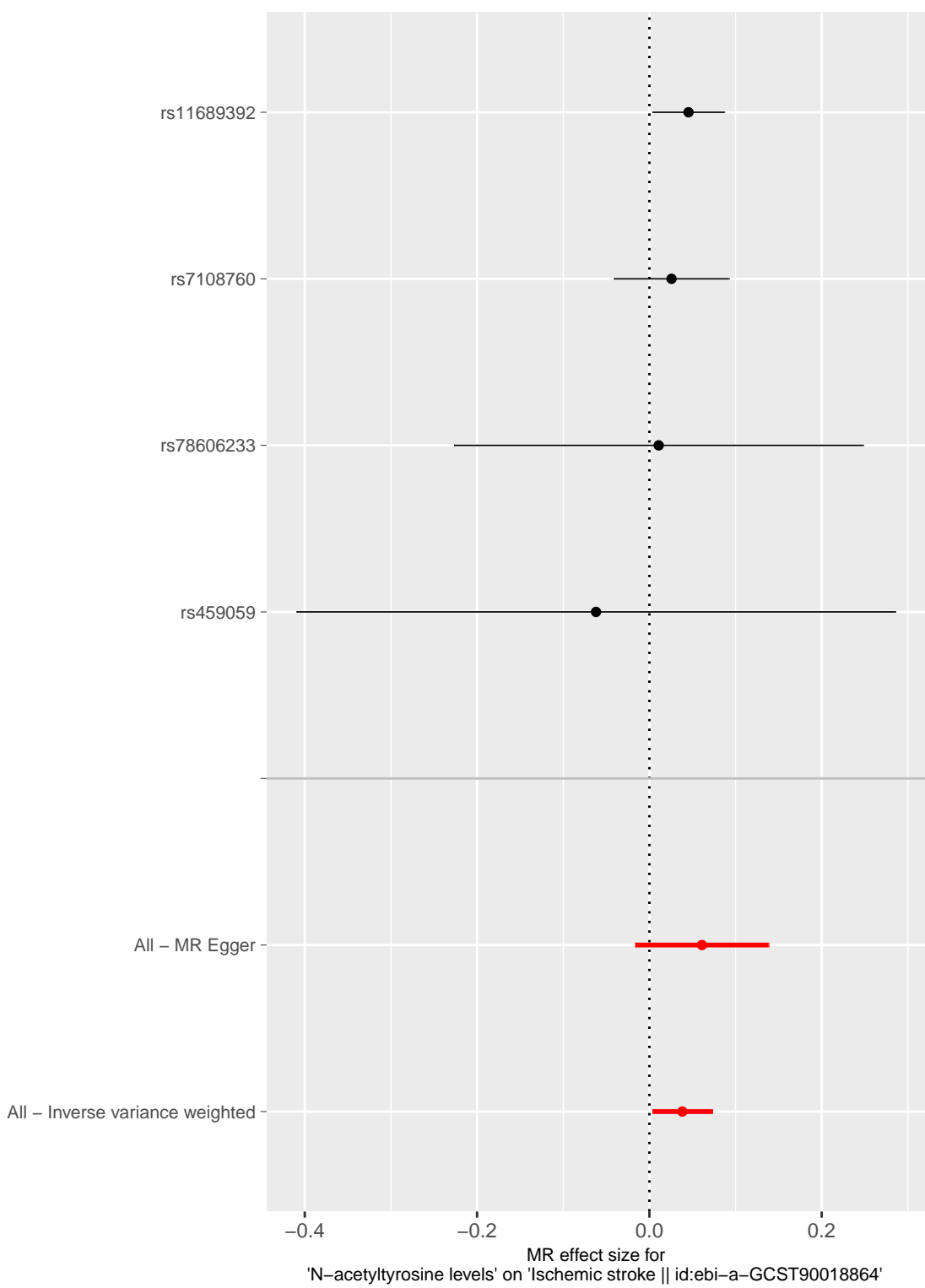

Supplement: Supplementary file 1 [file DataSheet1.ZIP › serum_metabolites/GCST90200675_forest_plot.pdf]

# MR Method

- Inverse variance weighted
- MR Egger

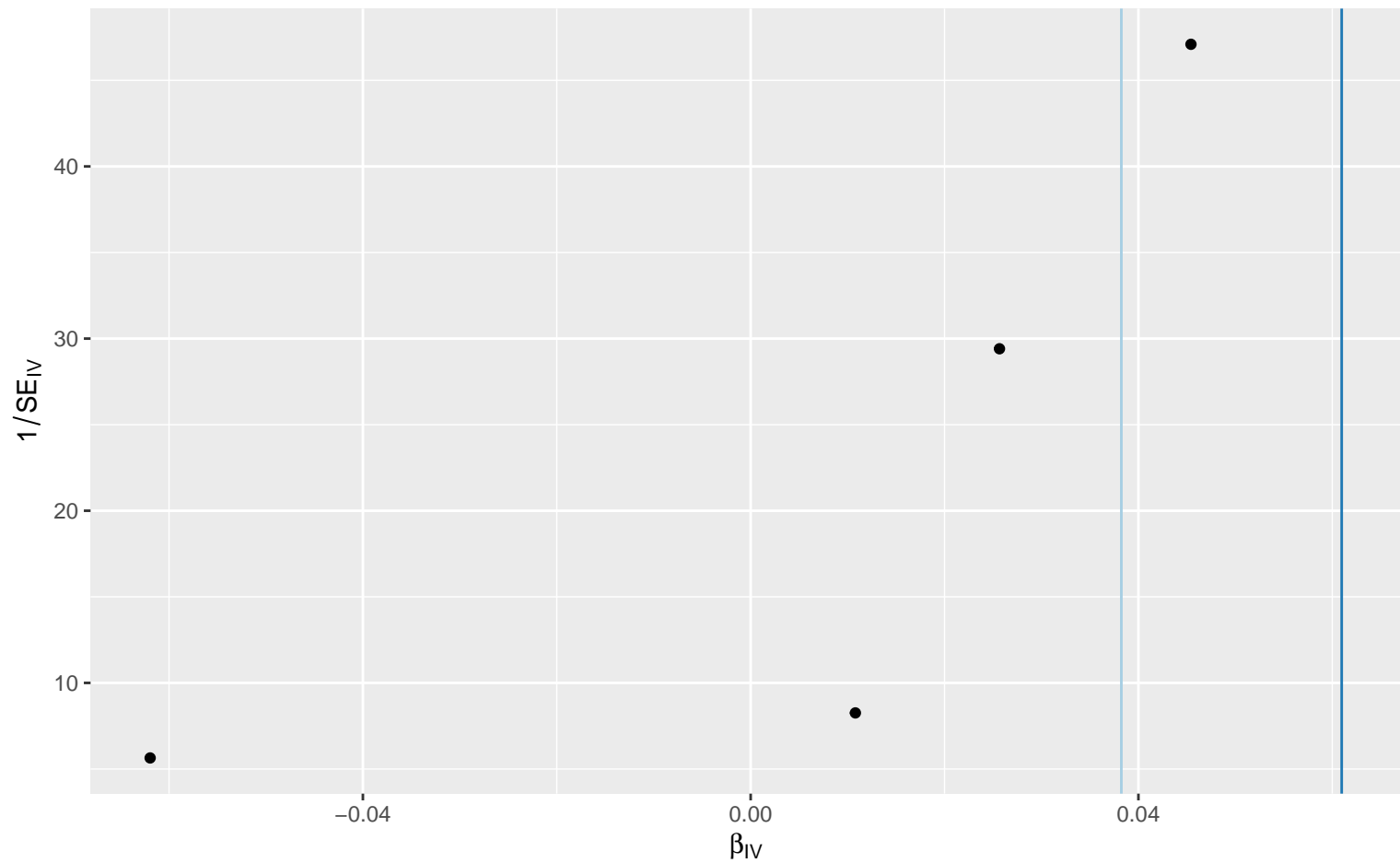

Supplement: Supplementary file 1 [file DataSheet1.ZIP › serum_metabolites/GCST90200675_funnel_plot.pdf]

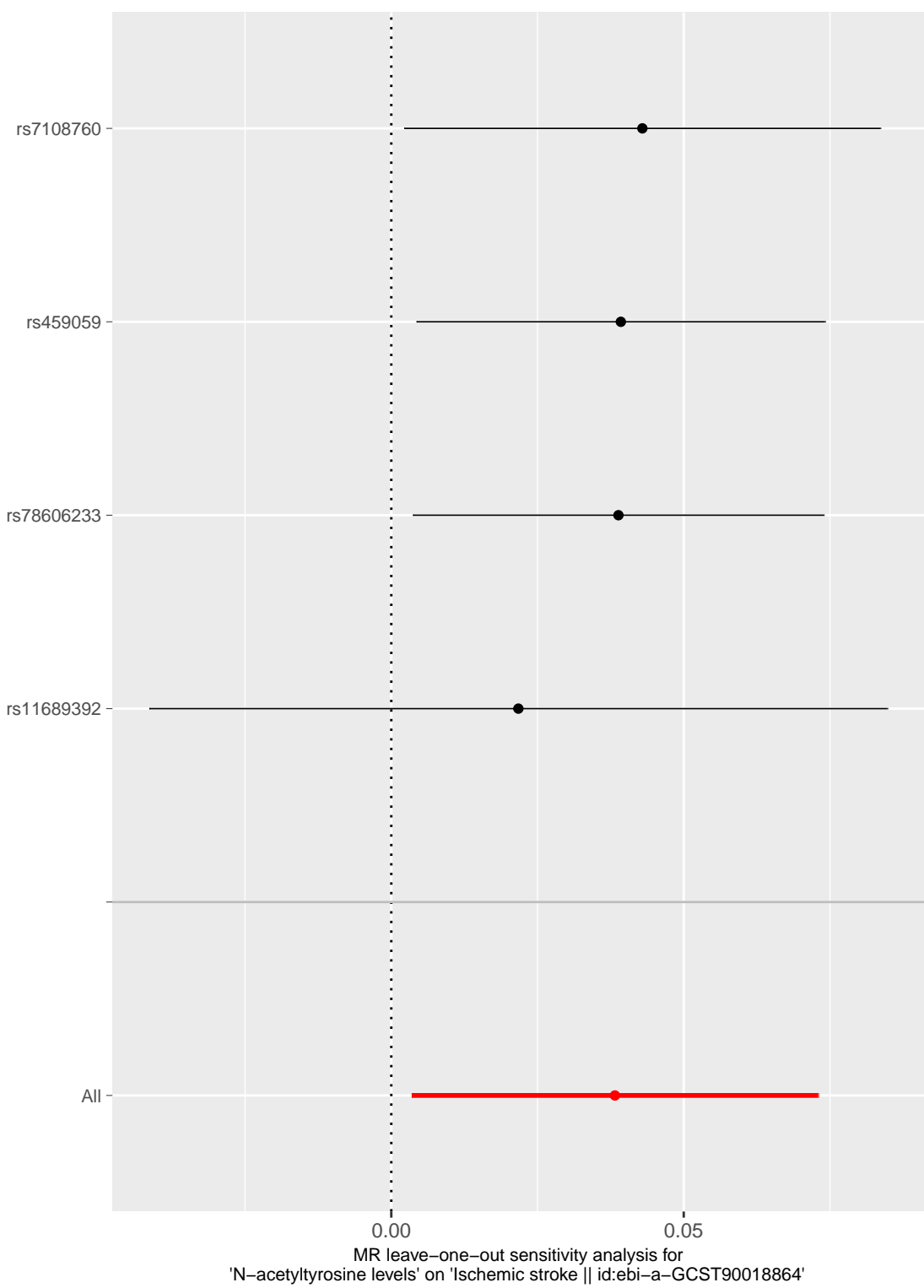

Supplement: Supplementary file 1 [file DataSheet1.ZIP › serum_metabolites/GCST90200675_leaveOneOut_plot.pdf]

# MR Method

- Inverse variance weighted
- MR Egger

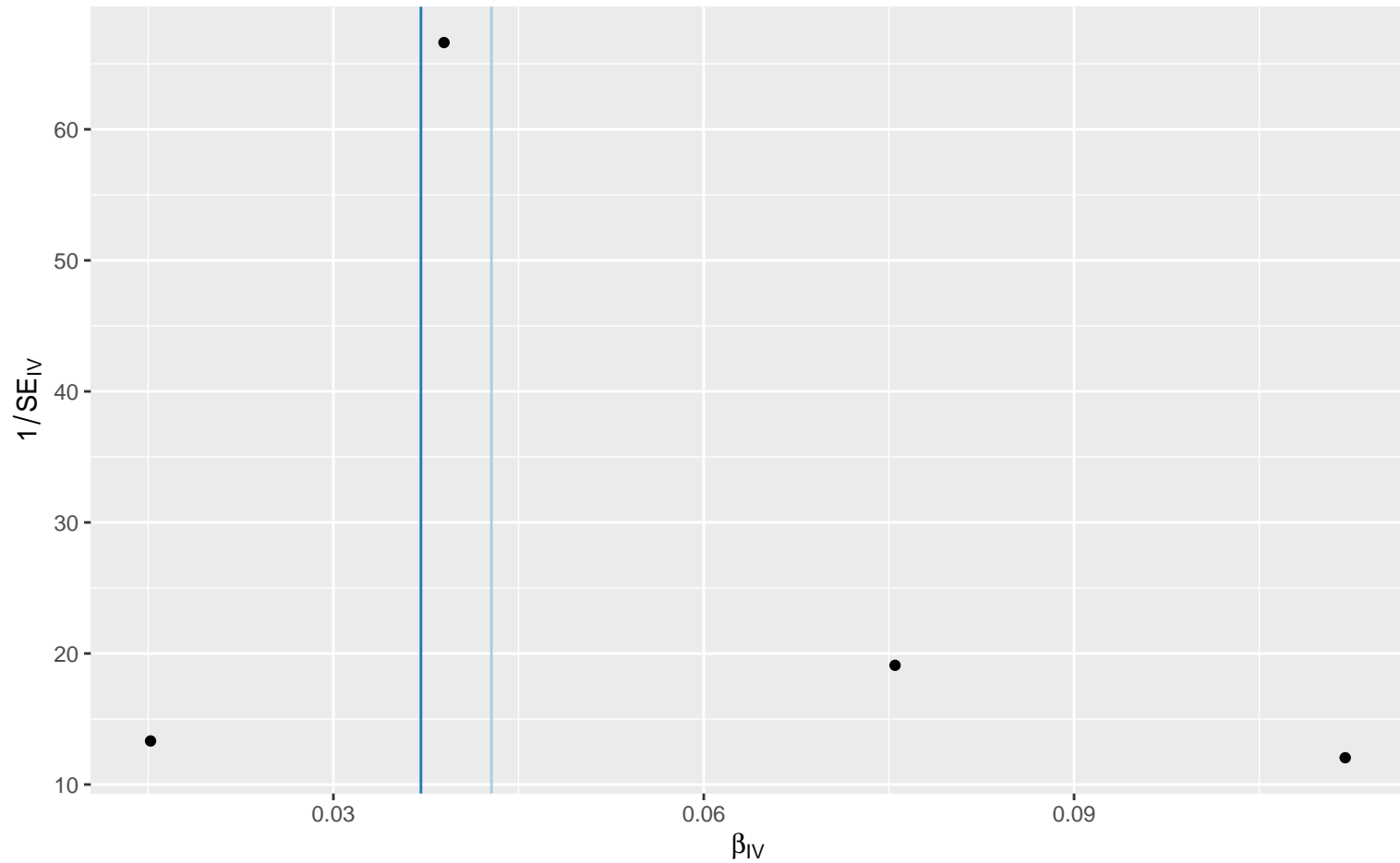

Supplement: Supplementary file 1 [file DataSheet1.ZIP › serum_metabolites/GCST90200680_funnel_plot.pdf]

# MR Test

- Inverse variance weighted
- MR Egger
- Simple mode
- Weighted mode

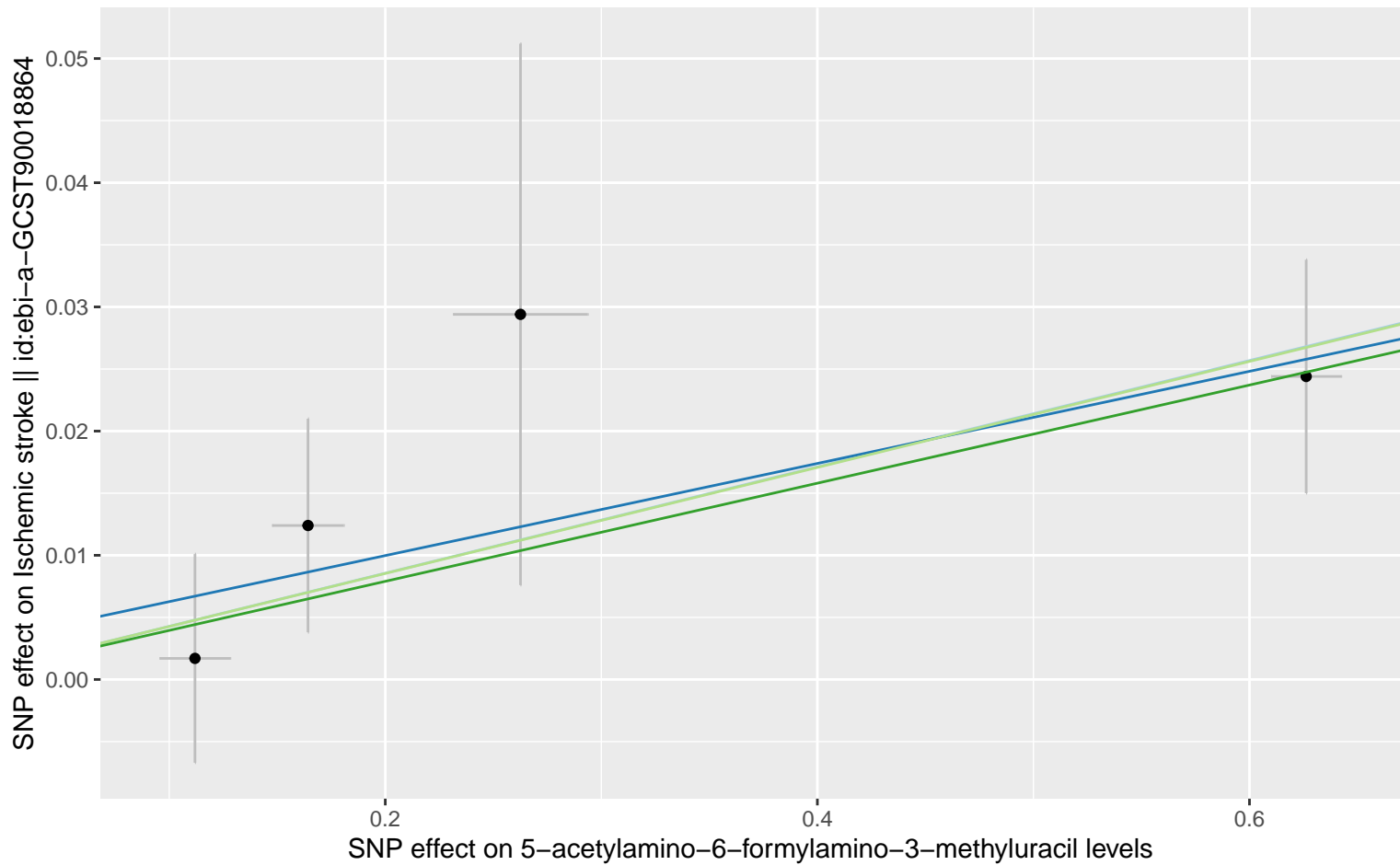

Supplement: Supplementary file 1 [file DataSheet1.ZIP › serum_metabolites/GCST90200680_scatter_plot.pdf]

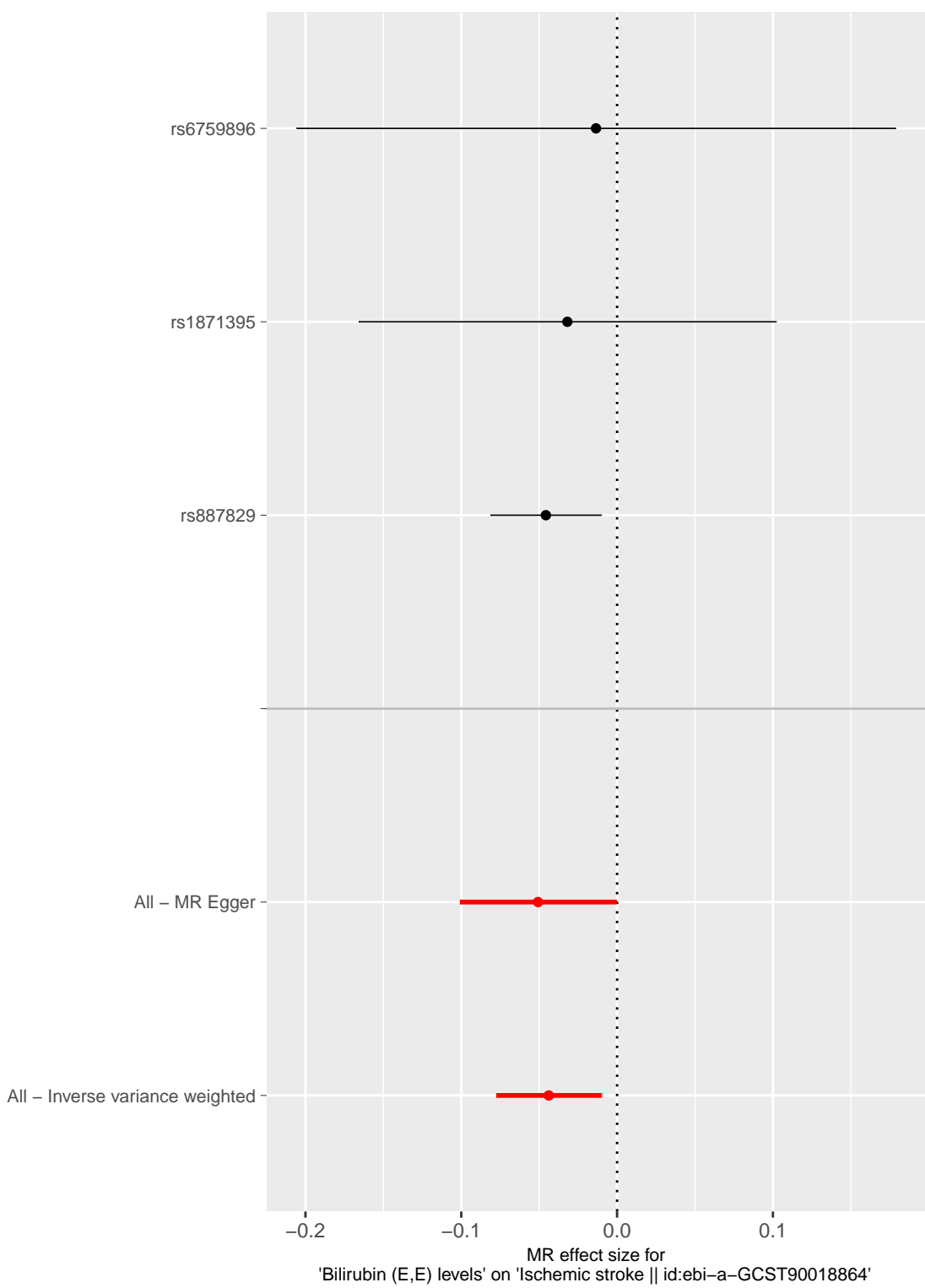

Supplement: Supplementary file 1 [file DataSheet1.ZIP › serum_metabolites/GCST90200686_forest_plot.pdf]

# MR Method

- Inverse variance weighted
- MR Egger

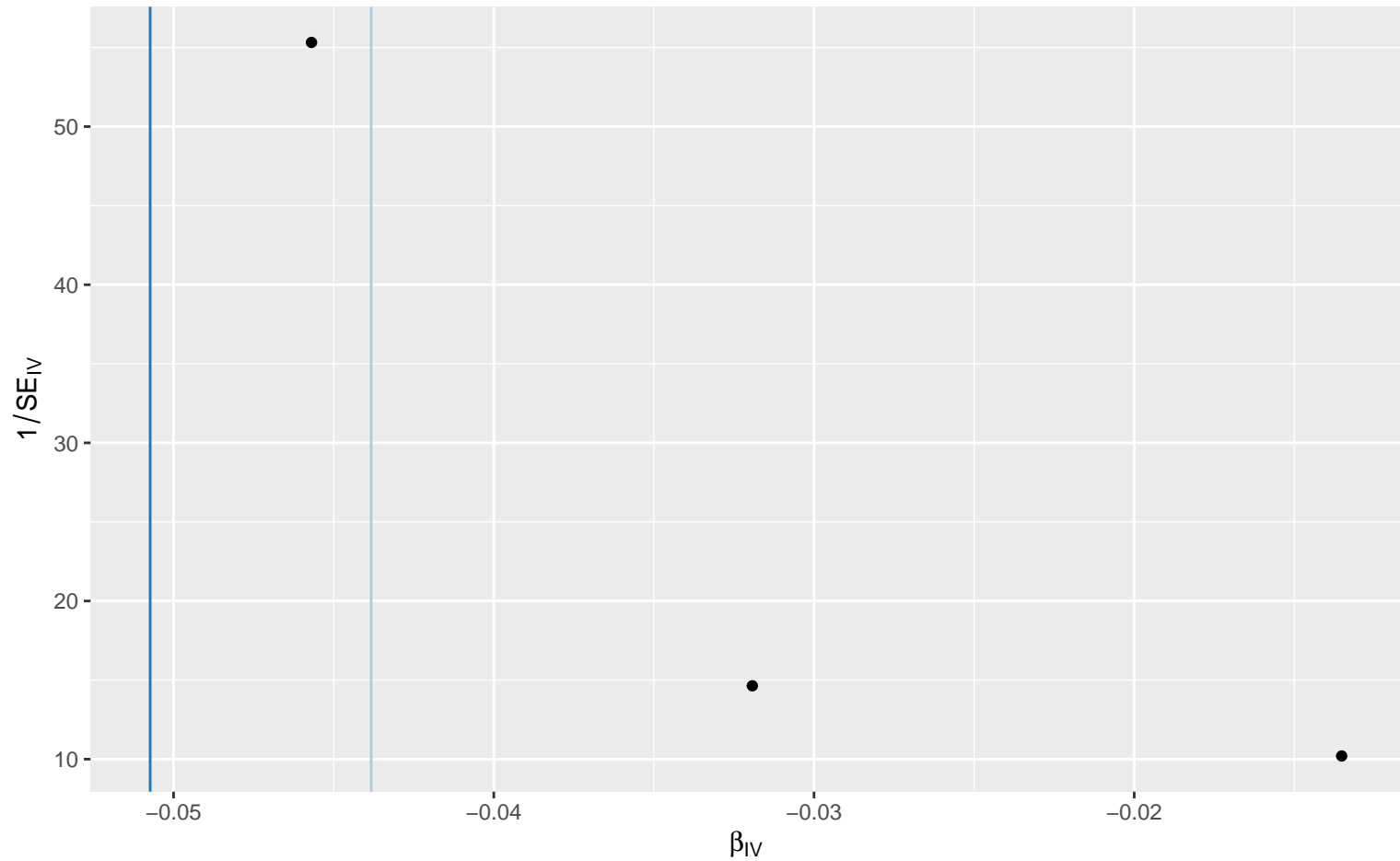

Supplement: Supplementary file 1 [file DataSheet1.ZIP › serum_metabolites/GCST90200686_funnel_plot.pdf]
