# Supplementary figures and images for: Causal relationship between human blood metabolites and risk of ischemic stroke: a Mendelian randomization study (part 1 of 2)
Source: Front Genet. 2024 Jan 19;15:1333454. doi: 10.3389/fgene.2024.1333454 (PMC10834680; doi:10.3389/fgene.2024.1333454)

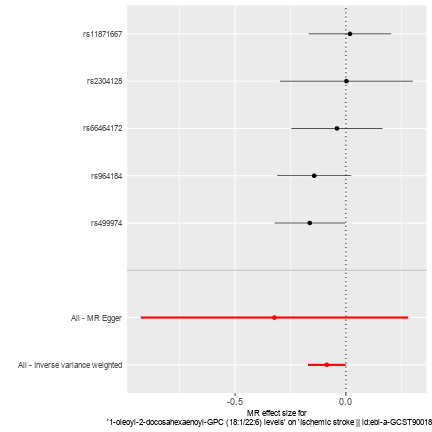

Supplement: Supplementary file 1 [file DataSheet1.ZIP › serum_metabolites/figure/1oleoyl2docosahexaenoylGPC_181226_levels_against_Ischemic_stroke__idebiaGCST90018864chunk5-1.png]

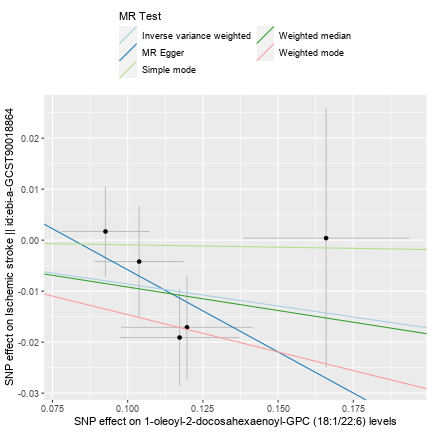

Supplement: Supplementary file 1 [file DataSheet1.ZIP › serum_metabolites/figure/1oleoyl2docosahexaenoylGPC_181226_levels_against_Ischemic_stroke__idebiaGCST90018864chunk6-1.png]

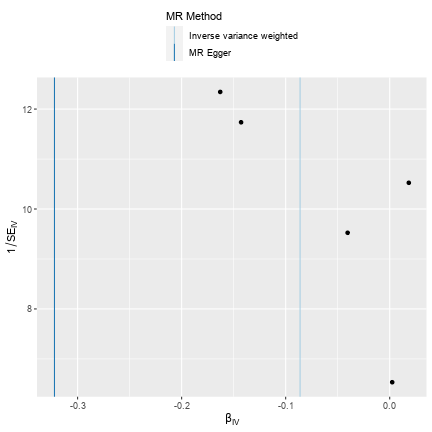

Supplement: Supplementary file 1 [file DataSheet1.ZIP › serum_metabolites/figure/1oleoyl2docosahexaenoylGPC_181226_levels_against_Ischemic_stroke__idebiaGCST90018864chunk7-1.png]

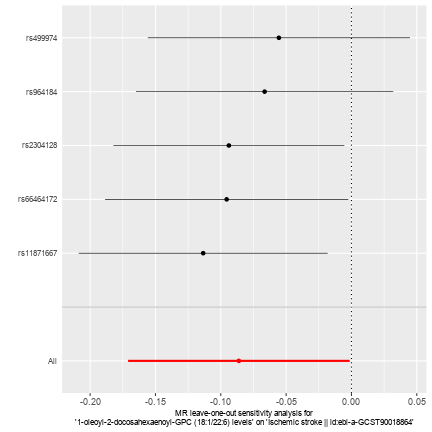

Supplement: Supplementary file 1 [file DataSheet1.ZIP › serum_metabolites/figure/1oleoyl2docosahexaenoylGPC_181226_levels_against_Ischemic_stroke__idebiaGCST90018864chunk8-1.png]

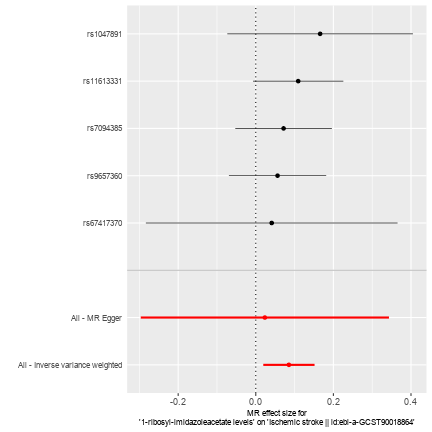

Supplement: Supplementary file 1 [file DataSheet1.ZIP › serum_metabolites/figure/1ribosylimidazoleacetate_levels_against_Ischemic_stroke__idebiaGCST90018864chunk5-1.png]

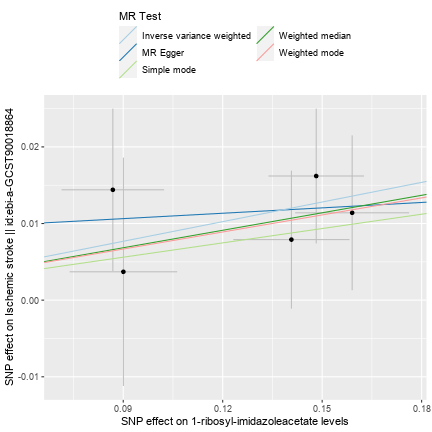

Supplement: Supplementary file 1 [file DataSheet1.ZIP › serum_metabolites/figure/1ribosylimidazoleacetate_levels_against_Ischemic_stroke__idebiaGCST90018864chunk6-1.png]

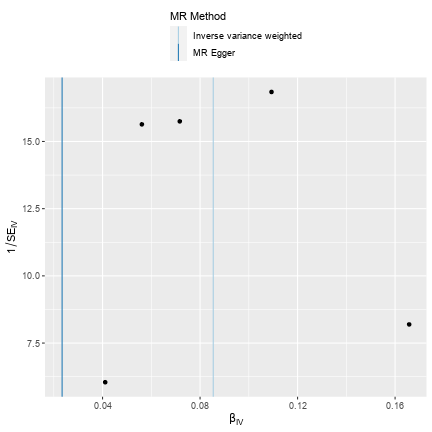

Supplement: Supplementary file 1 [file DataSheet1.ZIP › serum_metabolites/figure/1ribosylimidazoleacetate_levels_against_Ischemic_stroke__idebiaGCST90018864chunk7-1.png]

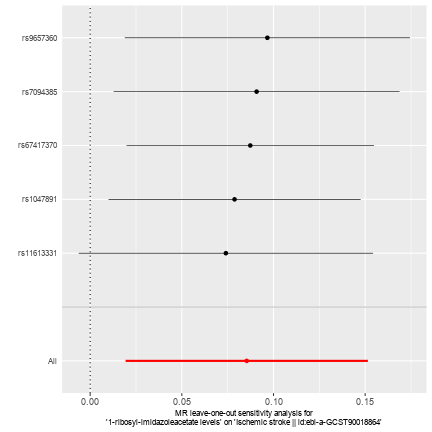

Supplement: Supplementary file 1 [file DataSheet1.ZIP › serum_metabolites/figure/1ribosylimidazoleacetate_levels_against_Ischemic_stroke__idebiaGCST90018864chunk8-1.png]

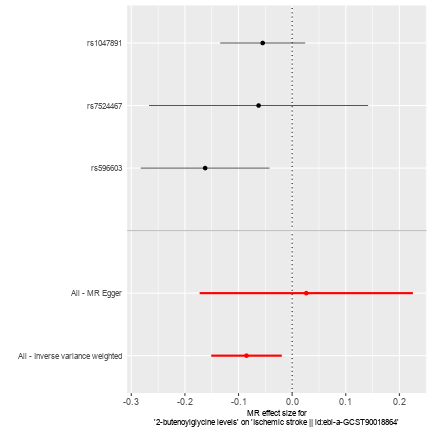

Supplement: Supplementary file 1 [file DataSheet1.ZIP › serum_metabolites/figure/2butenoylglycine_levels_against_Ischemic_stroke__idebiaGCST90018864chunk5-1.png]

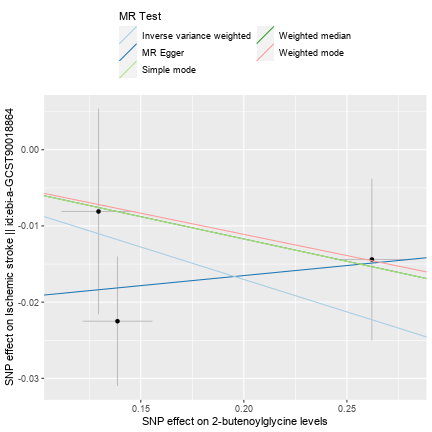

Supplement: Supplementary file 1 [file DataSheet1.ZIP › serum_metabolites/figure/2butenoylglycine_levels_against_Ischemic_stroke__idebiaGCST90018864chunk6-1.png]

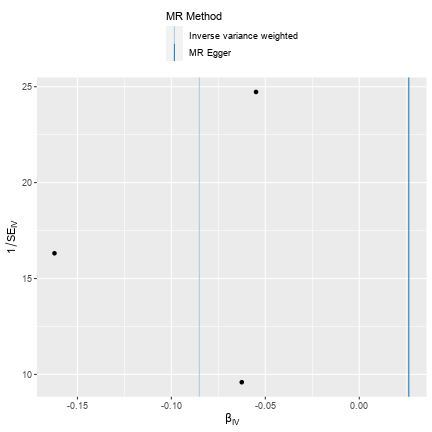

Supplement: Supplementary file 1 [file DataSheet1.ZIP › serum_metabolites/figure/2butenoylglycine_levels_against_Ischemic_stroke__idebiaGCST90018864chunk7-1.png]

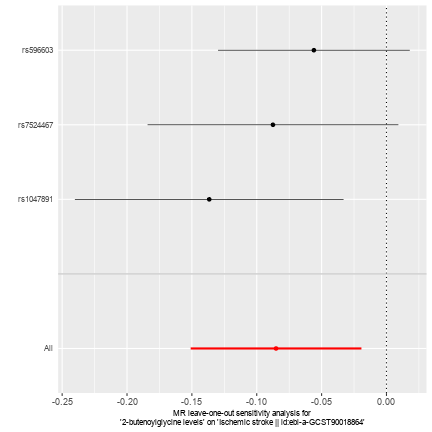

Supplement: Supplementary file 1 [file DataSheet1.ZIP › serum_metabolites/figure/2butenoylglycine_levels_against_Ischemic_stroke__idebiaGCST90018864chunk8-1.png]

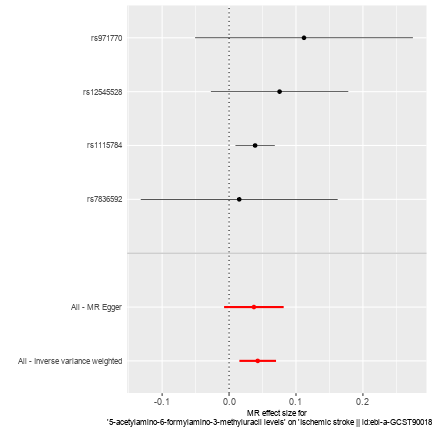

Supplement: Supplementary file 1 [file DataSheet1.ZIP › serum_metabolites/figure/5acetylamino6formylamino3methyluracil_levels_against_Ischemic_stroke__idebiaGCST90018864chunk5-1.png]

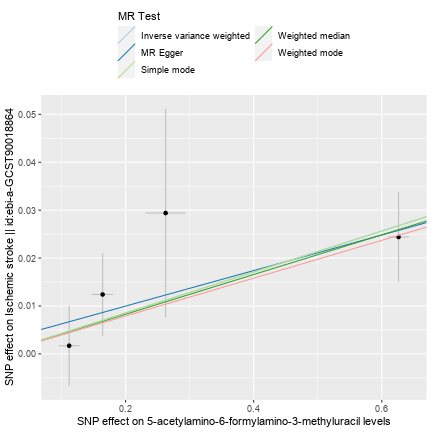

Supplement: Supplementary file 1 [file DataSheet1.ZIP › serum_metabolites/figure/5acetylamino6formylamino3methyluracil_levels_against_Ischemic_stroke__idebiaGCST90018864chunk6-1.png]

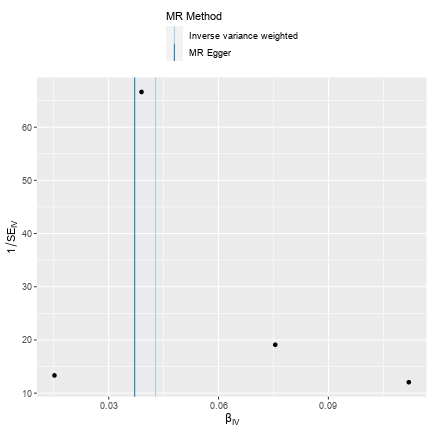

Supplement: Supplementary file 1 [file DataSheet1.ZIP › serum_metabolites/figure/5acetylamino6formylamino3methyluracil_levels_against_Ischemic_stroke__idebiaGCST90018864chunk7-1.png]

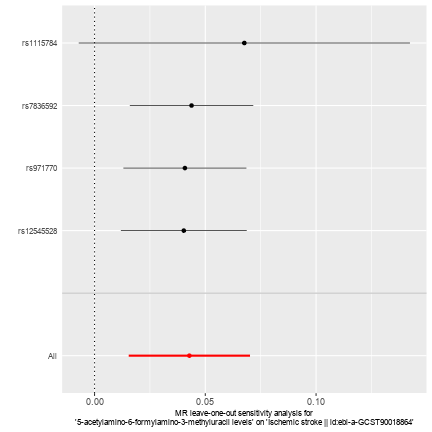

Supplement: Supplementary file 1 [file DataSheet1.ZIP › serum_metabolites/figure/5acetylamino6formylamino3methyluracil_levels_against_Ischemic_stroke__idebiaGCST90018864chunk8-1.png]

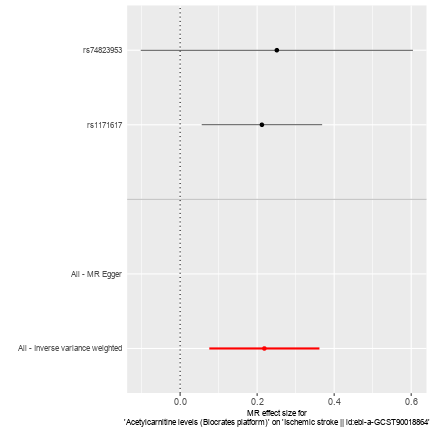

Supplement: Supplementary file 1 [file DataSheet1.ZIP › serum_metabolites/figure/Acetylcarnitine_levels_Biocrates_platform_against_Ischemic_stroke__idebiaGCST90018864chunk5-1.png]

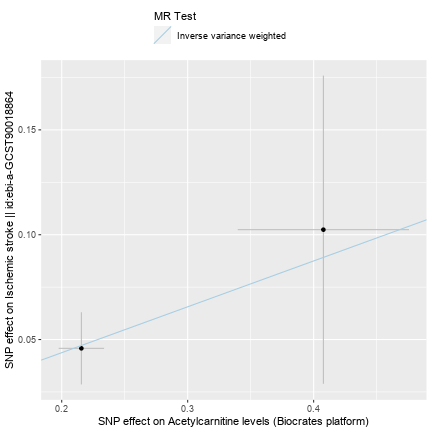

Supplement: Supplementary file 1 [file DataSheet1.ZIP › serum_metabolites/figure/Acetylcarnitine_levels_Biocrates_platform_against_Ischemic_stroke__idebiaGCST90018864chunk6-1.png]

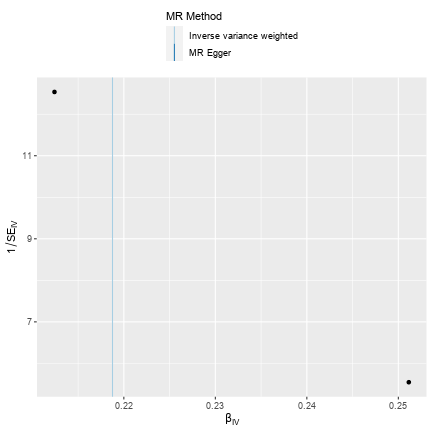

Supplement: Supplementary file 1 [file DataSheet1.ZIP › serum_metabolites/figure/Acetylcarnitine_levels_Biocrates_platform_against_Ischemic_stroke__idebiaGCST90018864chunk7-1.png]

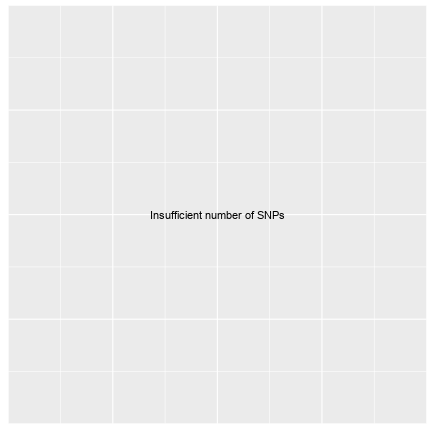

Supplement: Supplementary file 1 [file DataSheet1.ZIP › serum_metabolites/figure/Acetylcarnitine_levels_Biocrates_platform_against_Ischemic_stroke__idebiaGCST90018864chunk8-1.png]

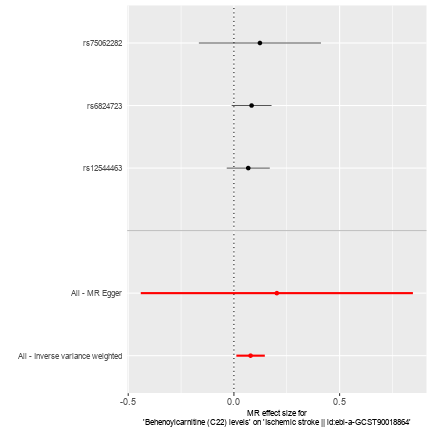

Supplement: Supplementary file 1 [file DataSheet1.ZIP › serum_metabolites/figure/Behenoylcarnitine_C22_levels_against_Ischemic_stroke__idebiaGCST90018864chunk5-1.png]

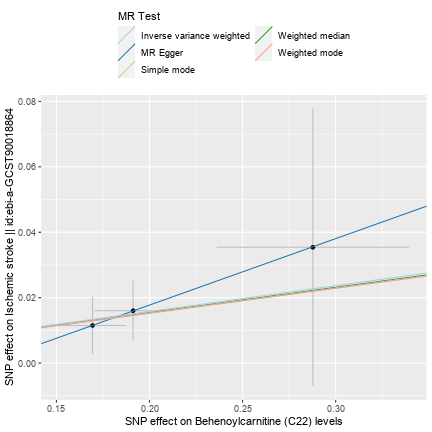

Supplement: Supplementary file 1 [file DataSheet1.ZIP › serum_metabolites/figure/Behenoylcarnitine_C22_levels_against_Ischemic_stroke__idebiaGCST90018864chunk6-1.png]

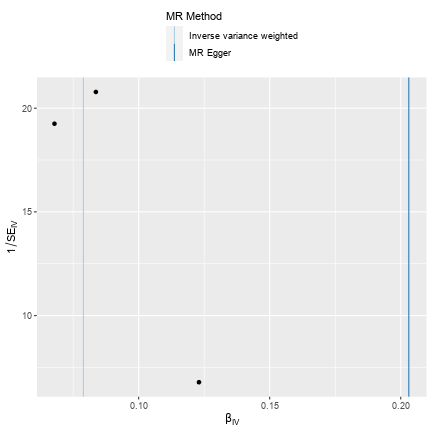

Supplement: Supplementary file 1 [file DataSheet1.ZIP › serum_metabolites/figure/Behenoylcarnitine_C22_levels_against_Ischemic_stroke__idebiaGCST90018864chunk7-1.png]

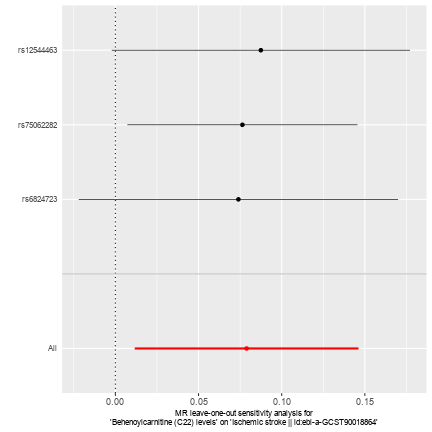

Supplement: Supplementary file 1 [file DataSheet1.ZIP › serum_metabolites/figure/Behenoylcarnitine_C22_levels_against_Ischemic_stroke__idebiaGCST90018864chunk8-1.png]

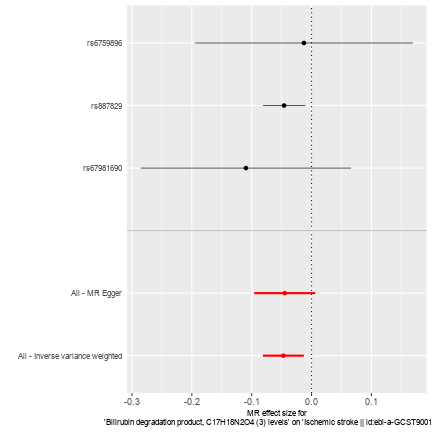

Supplement: Supplementary file 1 [file DataSheet1.ZIP › serum_metabolites/figure/Bilirubin_degradation_product_C17H18N2O4_3_levels_against_Ischemic_stroke__idebiaGCST90018864chunk5-1.png]

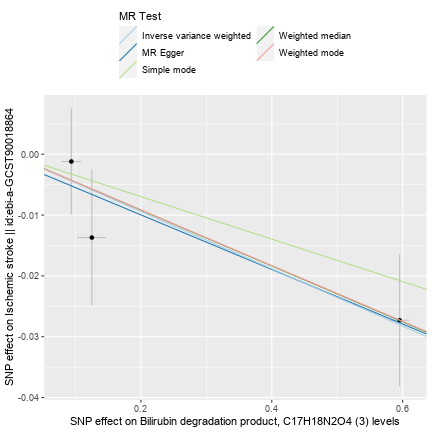

Supplement: Supplementary file 1 [file DataSheet1.ZIP › serum_metabolites/figure/Bilirubin_degradation_product_C17H18N2O4_3_levels_against_Ischemic_stroke__idebiaGCST90018864chunk6-1.png]

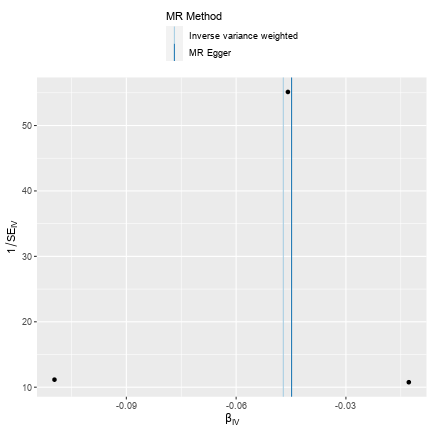

Supplement: Supplementary file 1 [file DataSheet1.ZIP › serum_metabolites/figure/Bilirubin_degradation_product_C17H18N2O4_3_levels_against_Ischemic_stroke__idebiaGCST90018864chunk7-1.png]

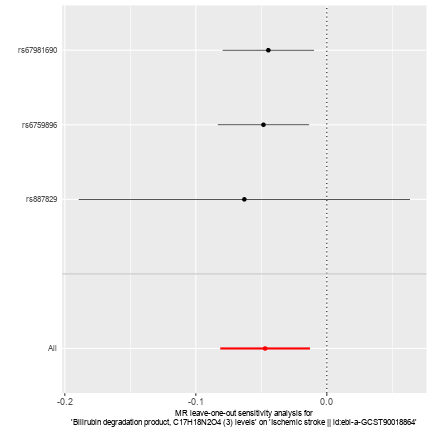

Supplement: Supplementary file 1 [file DataSheet1.ZIP › serum_metabolites/figure/Bilirubin_degradation_product_C17H18N2O4_3_levels_against_Ischemic_stroke__idebiaGCST90018864chunk8-1.png]

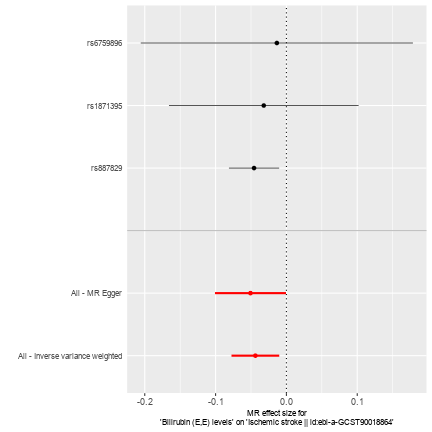

Supplement: Supplementary file 1 [file DataSheet1.ZIP › serum_metabolites/figure/Bilirubin_EE_levels_against_Ischemic_stroke__idebiaGCST90018864chunk5-1.png]

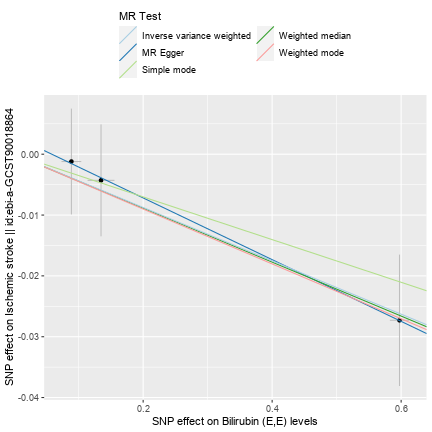

Supplement: Supplementary file 1 [file DataSheet1.ZIP › serum_metabolites/figure/Bilirubin_EE_levels_against_Ischemic_stroke__idebiaGCST90018864chunk6-1.png]

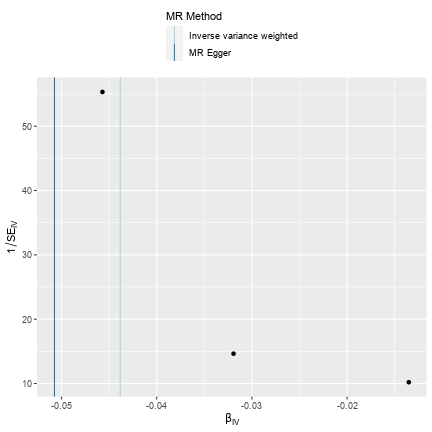

Supplement: Supplementary file 1 [file DataSheet1.ZIP › serum_metabolites/figure/Bilirubin_EE_levels_against_Ischemic_stroke__idebiaGCST90018864chunk7-1.png]

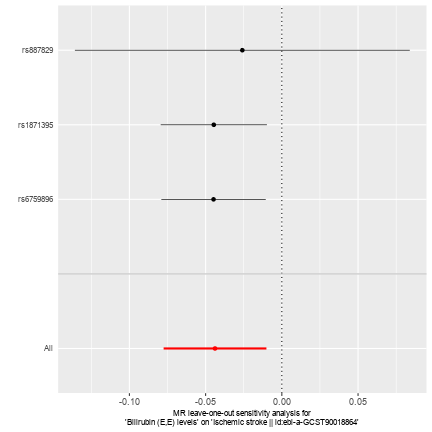

Supplement: Supplementary file 1 [file DataSheet1.ZIP › serum_metabolites/figure/Bilirubin_EE_levels_against_Ischemic_stroke__idebiaGCST90018864chunk8-1.png]

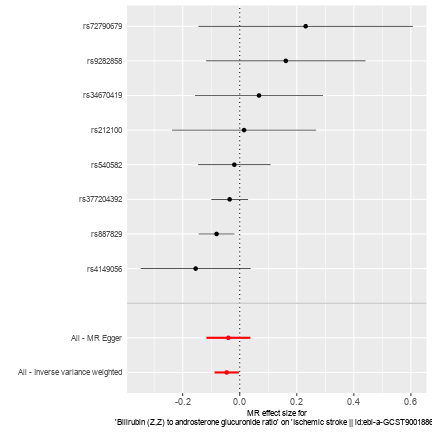

Supplement: Supplementary file 1 [file DataSheet1.ZIP › serum_metabolites/figure/Bilirubin_ZZ_to_androsterone_glucuronide_ratio_against_Ischemic_stroke__idebiaGCST90018864chunk5-1.png]

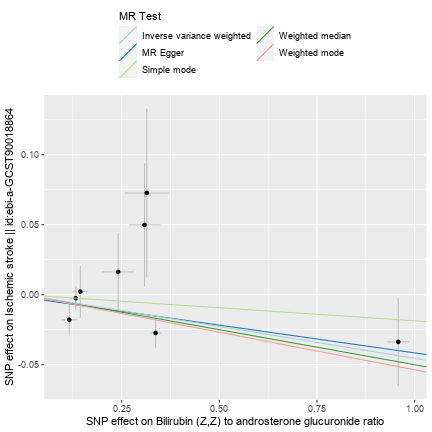

Supplement: Supplementary file 1 [file DataSheet1.ZIP › serum_metabolites/figure/Bilirubin_ZZ_to_androsterone_glucuronide_ratio_against_Ischemic_stroke__idebiaGCST90018864chunk6-1.png]

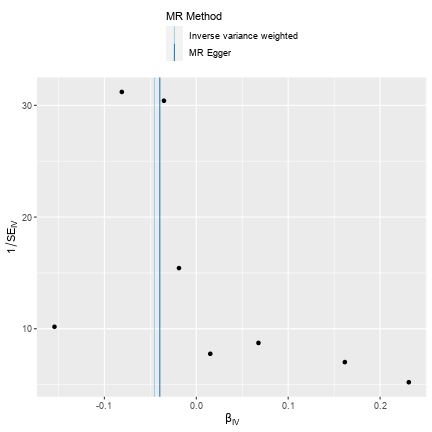

Supplement: Supplementary file 1 [file DataSheet1.ZIP › serum_metabolites/figure/Bilirubin_ZZ_to_androsterone_glucuronide_ratio_against_Ischemic_stroke__idebiaGCST90018864chunk7-1.png]

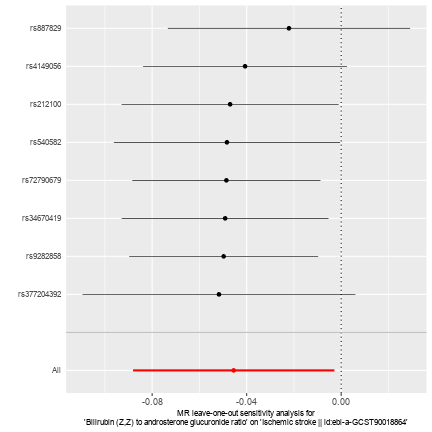

Supplement: Supplementary file 1 [file DataSheet1.ZIP › serum_metabolites/figure/Bilirubin_ZZ_to_androsterone_glucuronide_ratio_against_Ischemic_stroke__idebiaGCST90018864chunk8-1.png]

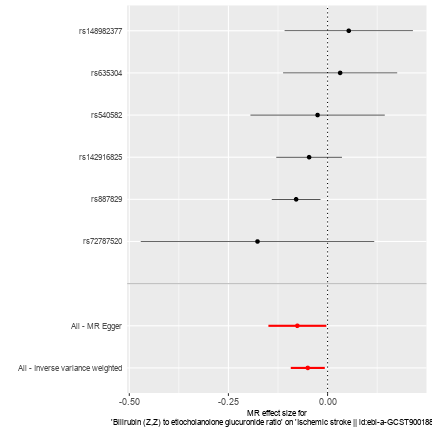

Supplement: Supplementary file 1 [file DataSheet1.ZIP › serum_metabolites/figure/Bilirubin_ZZ_to_etiocholanolone_glucuronide_ratio_against_Ischemic_stroke__idebiaGCST90018864chunk5-1.png]

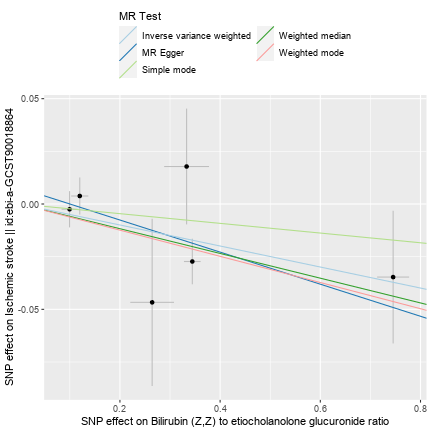

Supplement: Supplementary file 1 [file DataSheet1.ZIP › serum_metabolites/figure/Bilirubin_ZZ_to_etiocholanolone_glucuronide_ratio_against_Ischemic_stroke__idebiaGCST90018864chunk6-1.png]

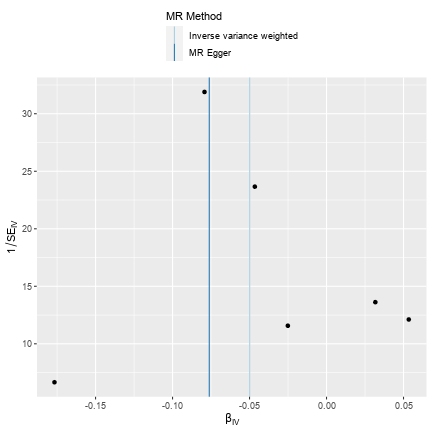

Supplement: Supplementary file 1 [file DataSheet1.ZIP › serum_metabolites/figure/Bilirubin_ZZ_to_etiocholanolone_glucuronide_ratio_against_Ischemic_stroke__idebiaGCST90018864chunk7-1.png]

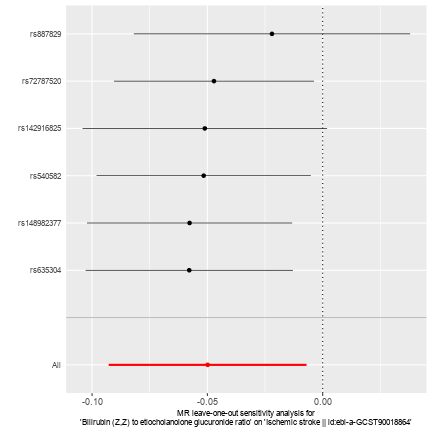

Supplement: Supplementary file 1 [file DataSheet1.ZIP › serum_metabolites/figure/Bilirubin_ZZ_to_etiocholanolone_glucuronide_ratio_against_Ischemic_stroke__idebiaGCST90018864chunk8-1.png]

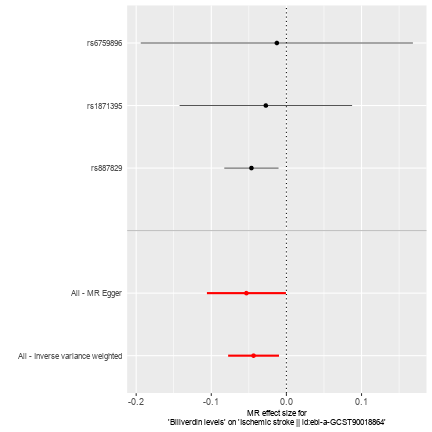

Supplement: Supplementary file 1 [file DataSheet1.ZIP › serum_metabolites/figure/Biliverdin_levels_against_Ischemic_stroke__idebiaGCST90018864chunk5-1.png]

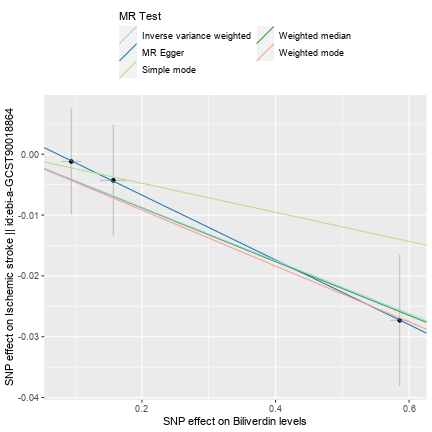

Supplement: Supplementary file 1 [file DataSheet1.ZIP › serum_metabolites/figure/Biliverdin_levels_against_Ischemic_stroke__idebiaGCST90018864chunk6-1.png]

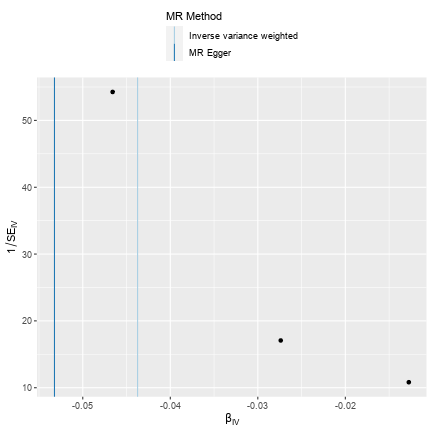

Supplement: Supplementary file 1 [file DataSheet1.ZIP › serum_metabolites/figure/Biliverdin_levels_against_Ischemic_stroke__idebiaGCST90018864chunk7-1.png]

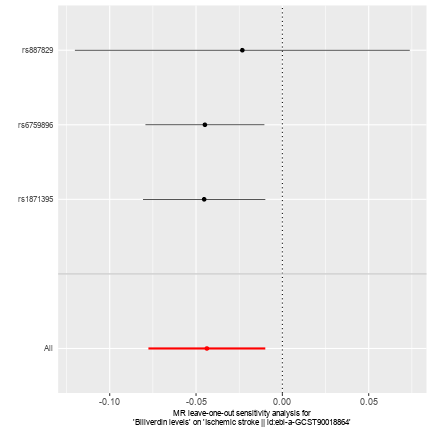

Supplement: Supplementary file 1 [file DataSheet1.ZIP › serum_metabolites/figure/Biliverdin_levels_against_Ischemic_stroke__idebiaGCST90018864chunk8-1.png]

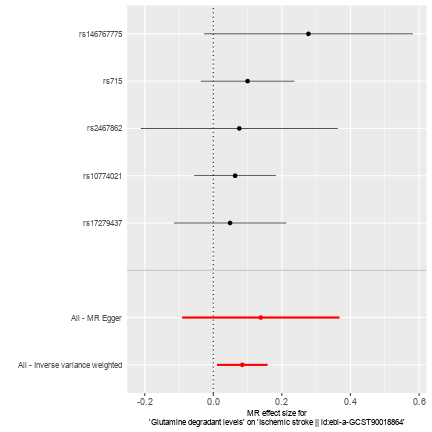

Supplement: Supplementary file 1 [file DataSheet1.ZIP › serum_metabolites/figure/Glutamine_degradant_levels_against_Ischemic_stroke__idebiaGCST90018864chunk5-1.png]

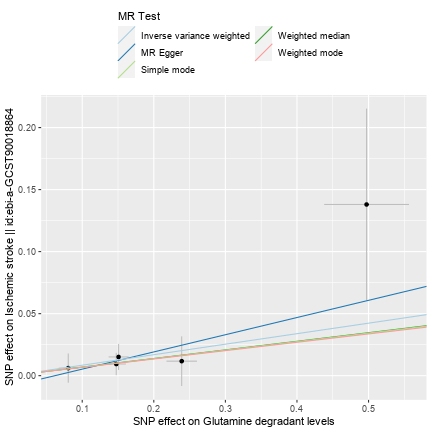

Supplement: Supplementary file 1 [file DataSheet1.ZIP › serum_metabolites/figure/Glutamine_degradant_levels_against_Ischemic_stroke__idebiaGCST90018864chunk6-1.png]

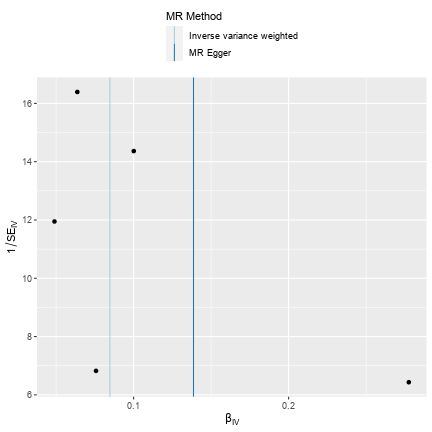

Supplement: Supplementary file 1 [file DataSheet1.ZIP › serum_metabolites/figure/Glutamine_degradant_levels_against_Ischemic_stroke__idebiaGCST90018864chunk7-1.png]

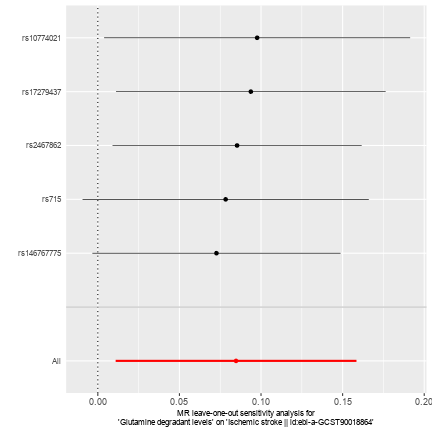

Supplement: Supplementary file 1 [file DataSheet1.ZIP › serum_metabolites/figure/Glutamine_degradant_levels_against_Ischemic_stroke__idebiaGCST90018864chunk8-1.png]

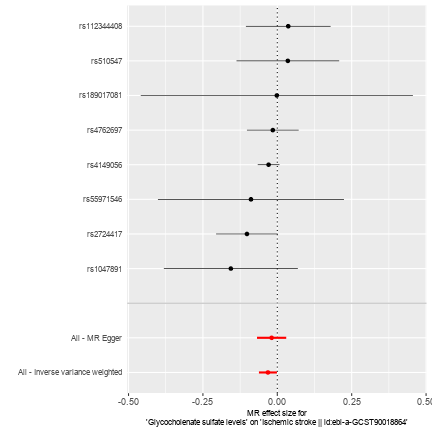

Supplement: Supplementary file 1 [file DataSheet1.ZIP › serum_metabolites/figure/Glycocholenate_sulfate_levels_against_Ischemic_stroke__idebiaGCST90018864chunk5-1.png]

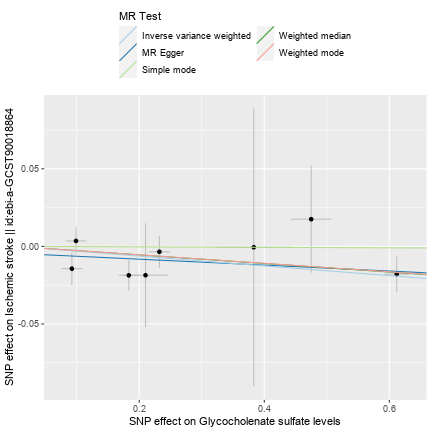

Supplement: Supplementary file 1 [file DataSheet1.ZIP › serum_metabolites/figure/Glycocholenate_sulfate_levels_against_Ischemic_stroke__idebiaGCST90018864chunk6-1.png]

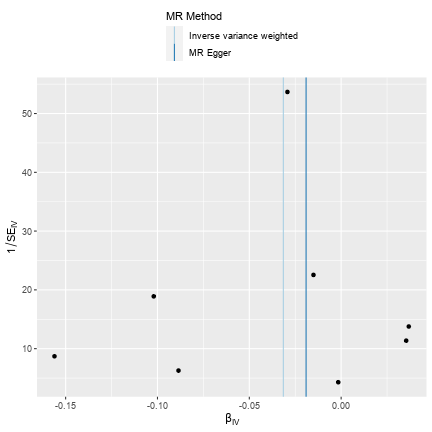

Supplement: Supplementary file 1 [file DataSheet1.ZIP › serum_metabolites/figure/Glycocholenate_sulfate_levels_against_Ischemic_stroke__idebiaGCST90018864chunk7-1.png]

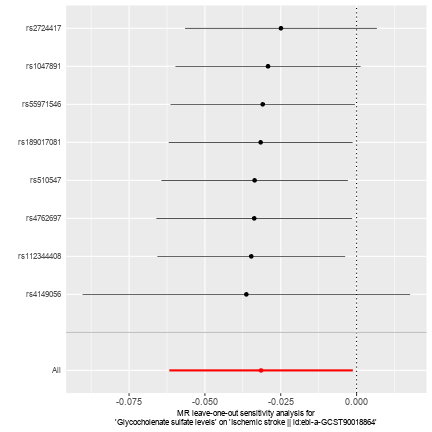

Supplement: Supplementary file 1 [file DataSheet1.ZIP › serum_metabolites/figure/Glycocholenate_sulfate_levels_against_Ischemic_stroke__idebiaGCST90018864chunk8-1.png]

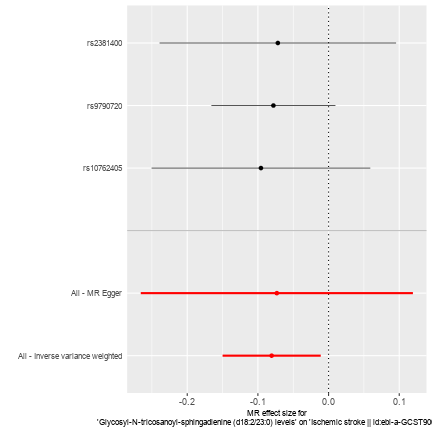

Supplement: Supplementary file 1 [file DataSheet1.ZIP › serum_metabolites/figure/GlycosylNtricosanoylsphingadienine_d182230_levels_against_Ischemic_stroke__idebiaGCST90018864chunk5-1.png]

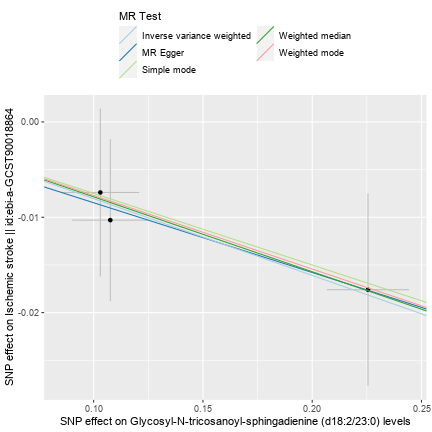

Supplement: Supplementary file 1 [file DataSheet1.ZIP › serum_metabolites/figure/GlycosylNtricosanoylsphingadienine_d182230_levels_against_Ischemic_stroke__idebiaGCST90018864chunk6-1.png]

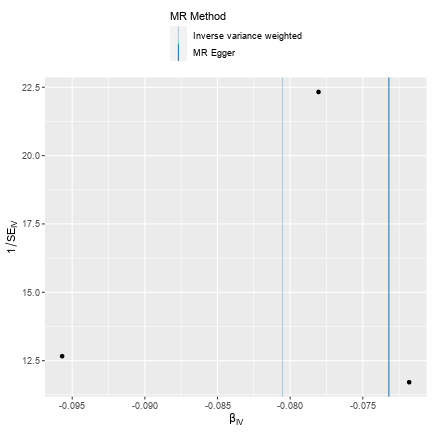

Supplement: Supplementary file 1 [file DataSheet1.ZIP › serum_metabolites/figure/GlycosylNtricosanoylsphingadienine_d182230_levels_against_Ischemic_stroke__idebiaGCST90018864chunk7-1.png]

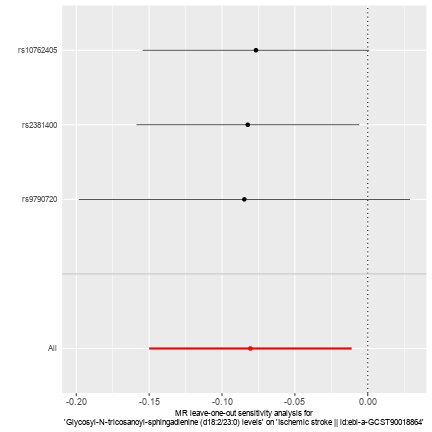

Supplement: Supplementary file 1 [file DataSheet1.ZIP › serum_metabolites/figure/GlycosylNtricosanoylsphingadienine_d182230_levels_against_Ischemic_stroke__idebiaGCST90018864chunk8-1.png]

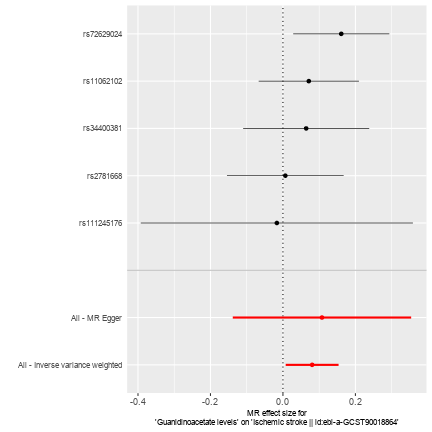

Supplement: Supplementary file 1 [file DataSheet1.ZIP › serum_metabolites/figure/Guanidinoacetate_levels_against_Ischemic_stroke__idebiaGCST90018864chunk5-1.png]

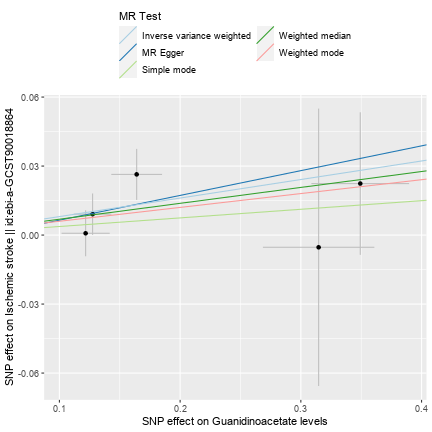

Supplement: Supplementary file 1 [file DataSheet1.ZIP › serum_metabolites/figure/Guanidinoacetate_levels_against_Ischemic_stroke__idebiaGCST90018864chunk6-1.png]

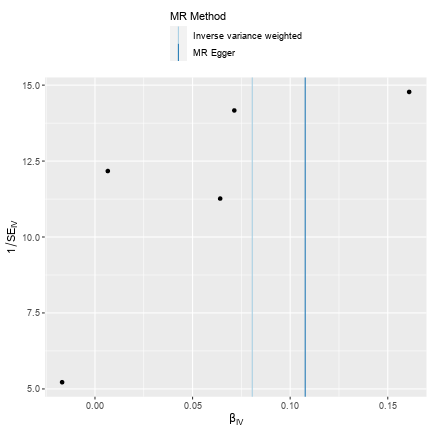

Supplement: Supplementary file 1 [file DataSheet1.ZIP › serum_metabolites/figure/Guanidinoacetate_levels_against_Ischemic_stroke__idebiaGCST90018864chunk7-1.png]

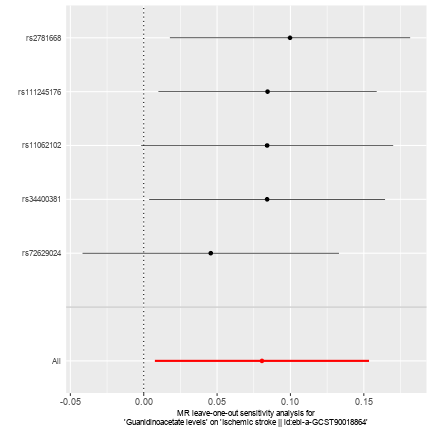

Supplement: Supplementary file 1 [file DataSheet1.ZIP › serum_metabolites/figure/Guanidinoacetate_levels_against_Ischemic_stroke__idebiaGCST90018864chunk8-1.png]

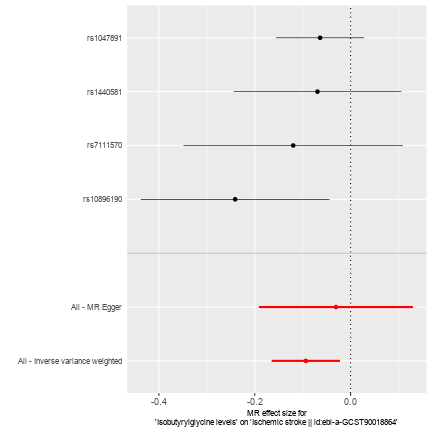

Supplement: Supplementary file 1 [file DataSheet1.ZIP › serum_metabolites/figure/Isobutyrylglycine_levels_against_Ischemic_stroke__idebiaGCST90018864chunk5-1.png]

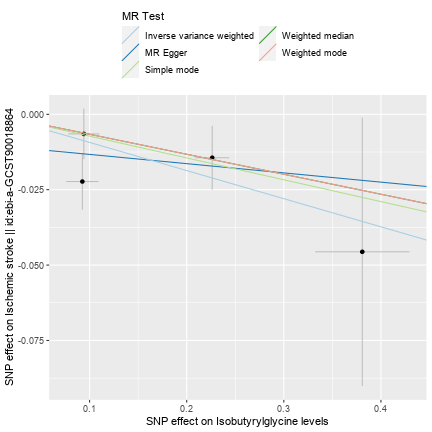

Supplement: Supplementary file 1 [file DataSheet1.ZIP › serum_metabolites/figure/Isobutyrylglycine_levels_against_Ischemic_stroke__idebiaGCST90018864chunk6-1.png]

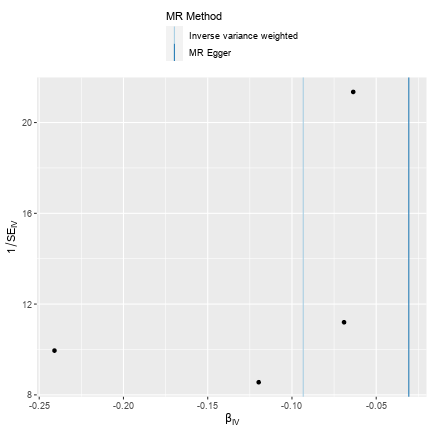

Supplement: Supplementary file 1 [file DataSheet1.ZIP › serum_metabolites/figure/Isobutyrylglycine_levels_against_Ischemic_stroke__idebiaGCST90018864chunk7-1.png]

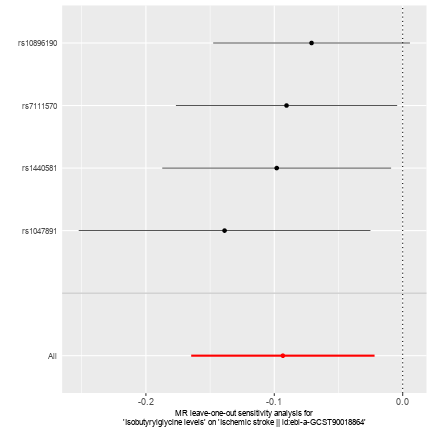

Supplement: Supplementary file 1 [file DataSheet1.ZIP › serum_metabolites/figure/Isobutyrylglycine_levels_against_Ischemic_stroke__idebiaGCST90018864chunk8-1.png]

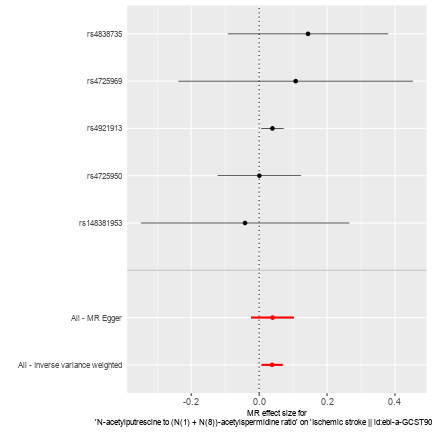

Supplement: Supplementary file 1 [file DataSheet1.ZIP › serum_metabolites/figure/Nacetylputrescine_to_N1__N8acetylspermidine_ratio_against_Ischemic_stroke__idebiaGCST90018864chunk5-1.png]

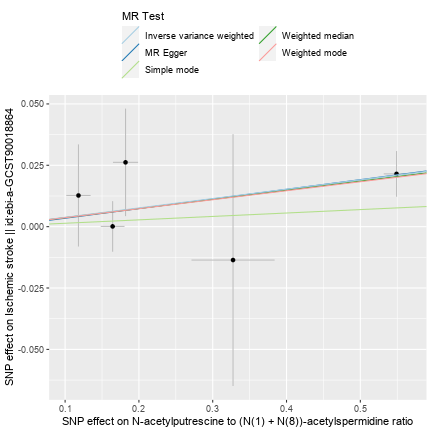

Supplement: Supplementary file 1 [file DataSheet1.ZIP › serum_metabolites/figure/Nacetylputrescine_to_N1__N8acetylspermidine_ratio_against_Ischemic_stroke__idebiaGCST90018864chunk6-1.png]

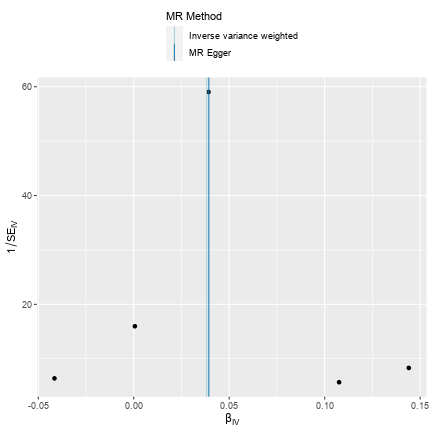

Supplement: Supplementary file 1 [file DataSheet1.ZIP › serum_metabolites/figure/Nacetylputrescine_to_N1__N8acetylspermidine_ratio_against_Ischemic_stroke__idebiaGCST90018864chunk7-1.png]

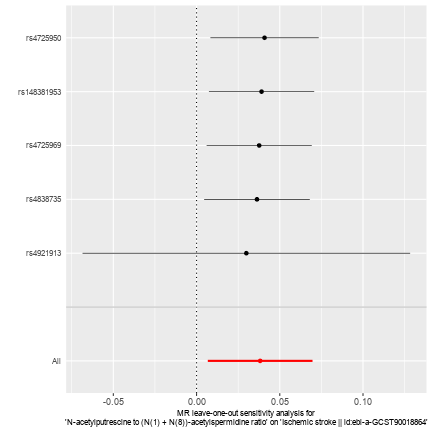

Supplement: Supplementary file 1 [file DataSheet1.ZIP › serum_metabolites/figure/Nacetylputrescine_to_N1__N8acetylspermidine_ratio_against_Ischemic_stroke__idebiaGCST90018864chunk8-1.png]

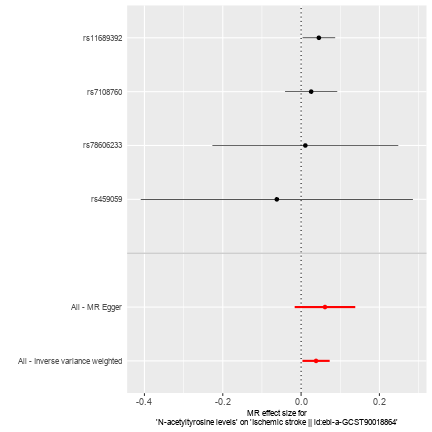

Supplement: Supplementary file 1 [file DataSheet1.ZIP › serum_metabolites/figure/Nacetyltyrosine_levels_against_Ischemic_stroke__idebiaGCST90018864chunk5-1.png]

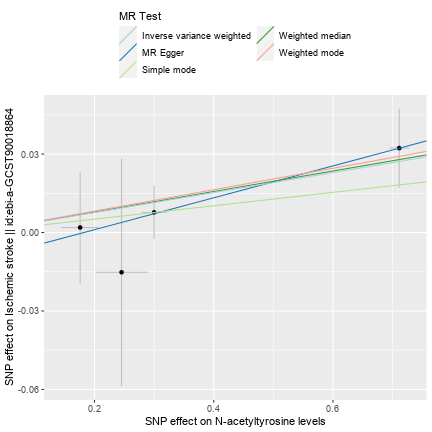

Supplement: Supplementary file 1 [file DataSheet1.ZIP › serum_metabolites/figure/Nacetyltyrosine_levels_against_Ischemic_stroke__idebiaGCST90018864chunk6-1.png]

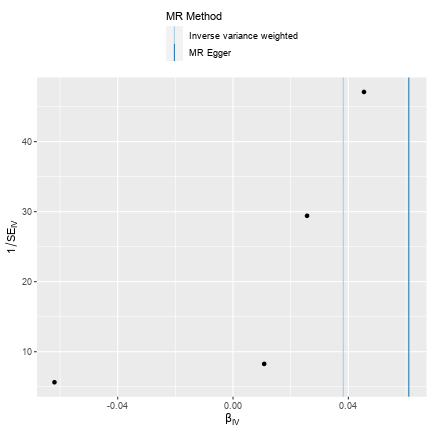

Supplement: Supplementary file 1 [file DataSheet1.ZIP › serum_metabolites/figure/Nacetyltyrosine_levels_against_Ischemic_stroke__idebiaGCST90018864chunk7-1.png]

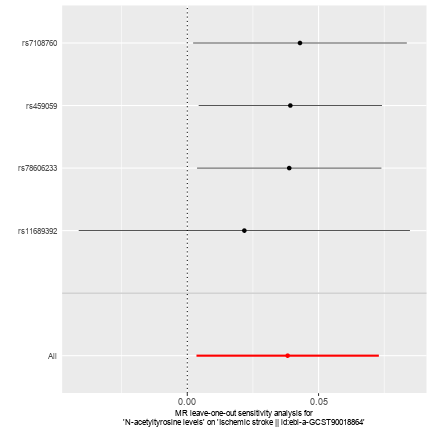

Supplement: Supplementary file 1 [file DataSheet1.ZIP › serum_metabolites/figure/Nacetyltyrosine_levels_against_Ischemic_stroke__idebiaGCST90018864chunk8-1.png]

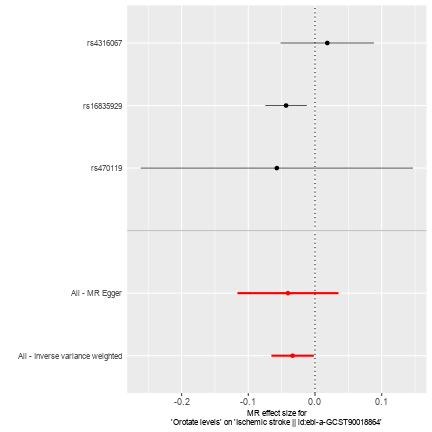

Supplement: Supplementary file 1 [file DataSheet1.ZIP › serum_metabolites/figure/Orotate_levels_against_Ischemic_stroke__idebiaGCST90018864chunk5-1.png]

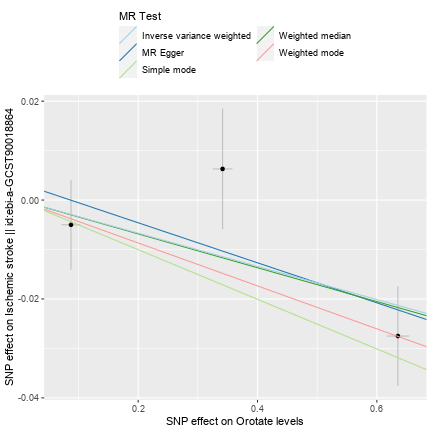

Supplement: Supplementary file 1 [file DataSheet1.ZIP › serum_metabolites/figure/Orotate_levels_against_Ischemic_stroke__idebiaGCST90018864chunk6-1.png]

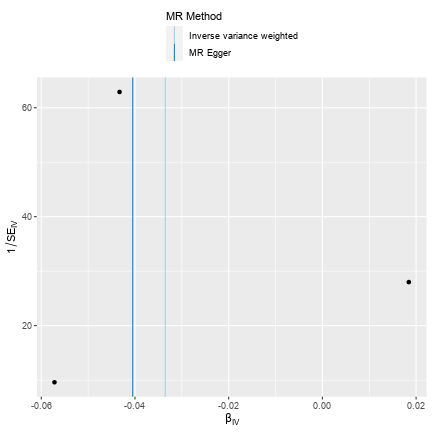

Supplement: Supplementary file 1 [file DataSheet1.ZIP › serum_metabolites/figure/Orotate_levels_against_Ischemic_stroke__idebiaGCST90018864chunk7-1.png]

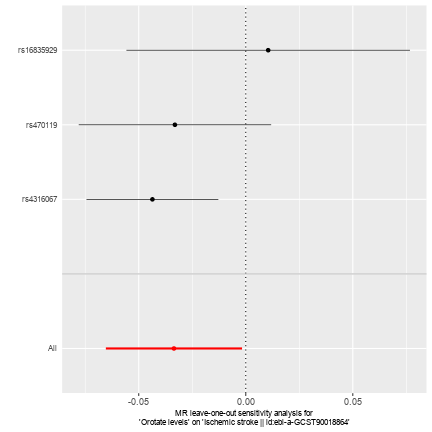

Supplement: Supplementary file 1 [file DataSheet1.ZIP › serum_metabolites/figure/Orotate_levels_against_Ischemic_stroke__idebiaGCST90018864chunk8-1.png]

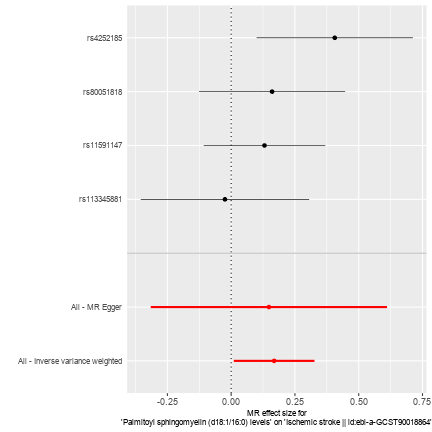

Supplement: Supplementary file 1 [file DataSheet1.ZIP › serum_metabolites/figure/Palmitoyl_sphingomyelin_d181160_levels_against_Ischemic_stroke__idebiaGCST90018864chunk5-1.png]

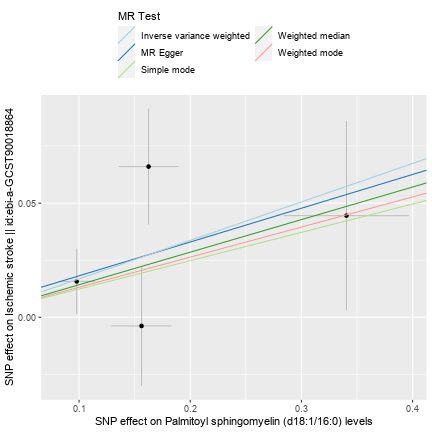

Supplement: Supplementary file 1 [file DataSheet1.ZIP › serum_metabolites/figure/Palmitoyl_sphingomyelin_d181160_levels_against_Ischemic_stroke__idebiaGCST90018864chunk6-1.png]

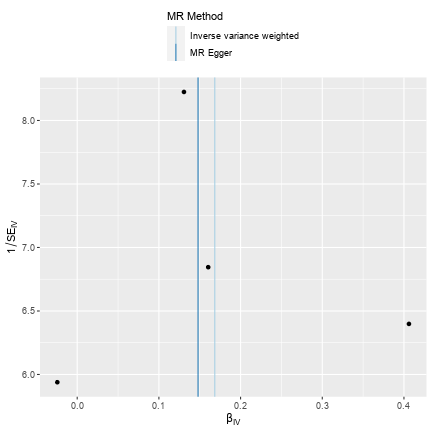

Supplement: Supplementary file 1 [file DataSheet1.ZIP › serum_metabolites/figure/Palmitoyl_sphingomyelin_d181160_levels_against_Ischemic_stroke__idebiaGCST90018864chunk7-1.png]

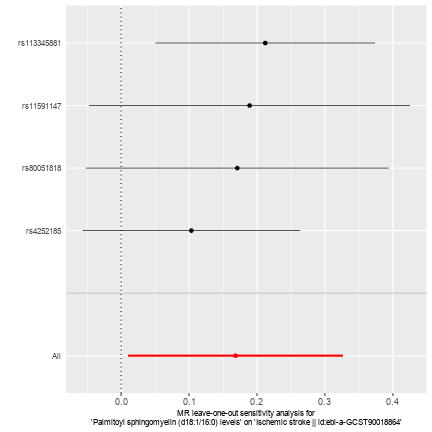

Supplement: Supplementary file 1 [file DataSheet1.ZIP › serum_metabolites/figure/Palmitoyl_sphingomyelin_d181160_levels_against_Ischemic_stroke__idebiaGCST90018864chunk8-1.png]

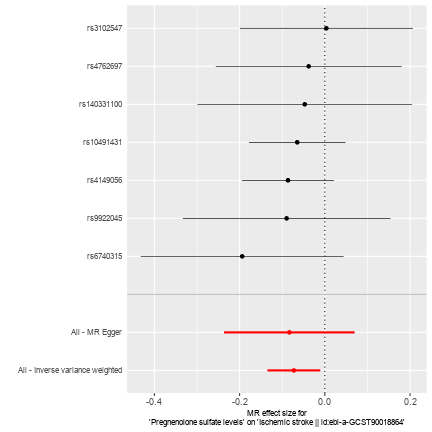

Supplement: Supplementary file 1 [file DataSheet1.ZIP › serum_metabolites/figure/Pregnenolone_sulfate_levels_against_Ischemic_stroke__idebiaGCST90018864chunk5-1.png]

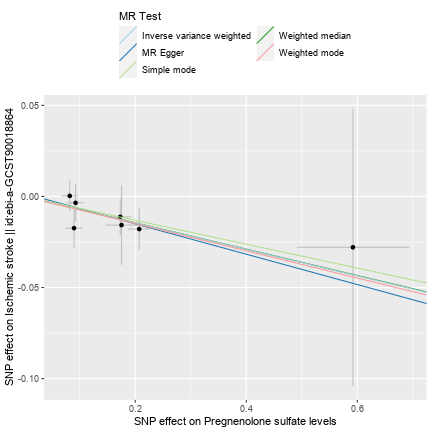

Supplement: Supplementary file 1 [file DataSheet1.ZIP › serum_metabolites/figure/Pregnenolone_sulfate_levels_against_Ischemic_stroke__idebiaGCST90018864chunk6-1.png]

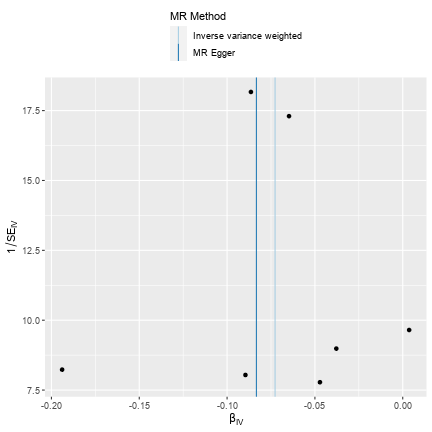

Supplement: Supplementary file 1 [file DataSheet1.ZIP › serum_metabolites/figure/Pregnenolone_sulfate_levels_against_Ischemic_stroke__idebiaGCST90018864chunk7-1.png]

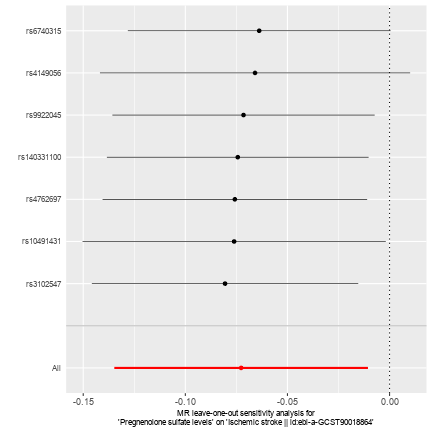

Supplement: Supplementary file 1 [file DataSheet1.ZIP › serum_metabolites/figure/Pregnenolone_sulfate_levels_against_Ischemic_stroke__idebiaGCST90018864chunk8-1.png]

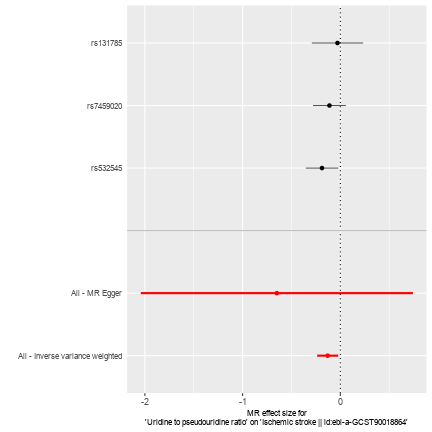

Supplement: Supplementary file 1 [file DataSheet1.ZIP › serum_metabolites/figure/Uridine_to_pseudouridine_ratio_against_Ischemic_stroke__idebiaGCST90018864chunk5-1.png]

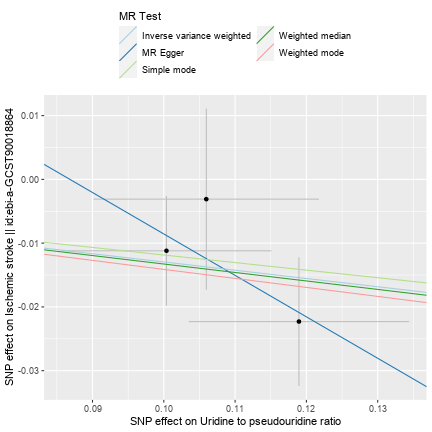

Supplement: Supplementary file 1 [file DataSheet1.ZIP › serum_metabolites/figure/Uridine_to_pseudouridine_ratio_against_Ischemic_stroke__idebiaGCST90018864chunk6-1.png]

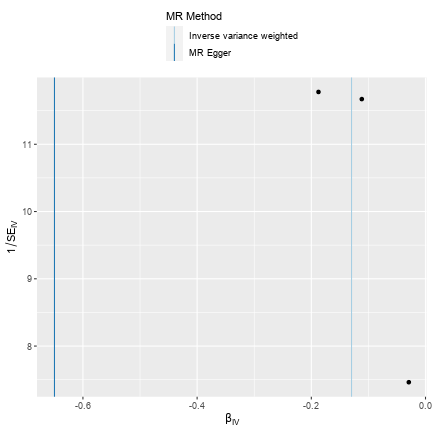

Supplement: Supplementary file 1 [file DataSheet1.ZIP › serum_metabolites/figure/Uridine_to_pseudouridine_ratio_against_Ischemic_stroke__idebiaGCST90018864chunk7-1.png]

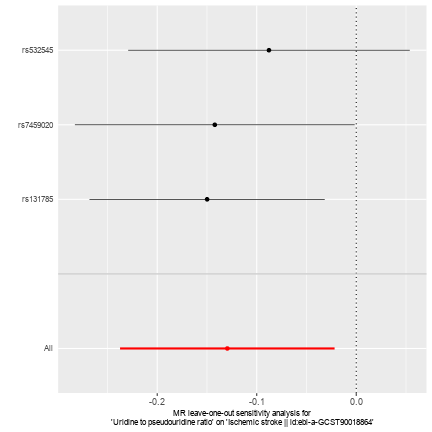

Supplement: Supplementary file 1 [file DataSheet1.ZIP › serum_metabolites/figure/Uridine_to_pseudouridine_ratio_against_Ischemic_stroke__idebiaGCST90018864chunk8-1.png]

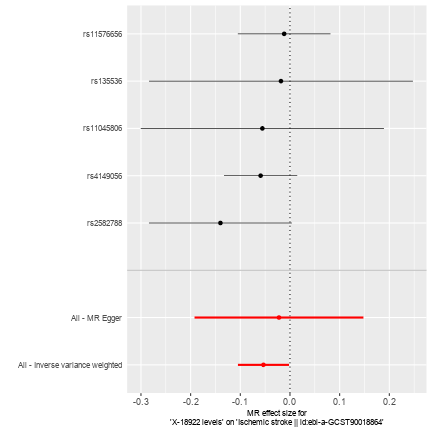

Supplement: Supplementary file 1 [file DataSheet1.ZIP › serum_metabolites/figure/X18922_levels_against_Ischemic_stroke__idebiaGCST90018864chunk5-1.png]

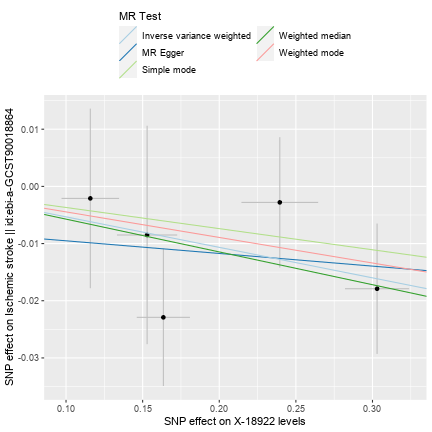

Supplement: Supplementary file 1 [file DataSheet1.ZIP › serum_metabolites/figure/X18922_levels_against_Ischemic_stroke__idebiaGCST90018864chunk6-1.png]

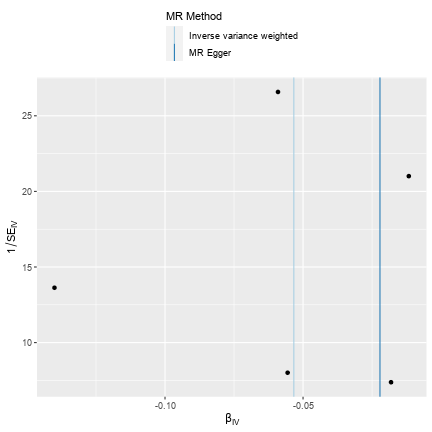

Supplement: Supplementary file 1 [file DataSheet1.ZIP › serum_metabolites/figure/X18922_levels_against_Ischemic_stroke__idebiaGCST90018864chunk7-1.png]

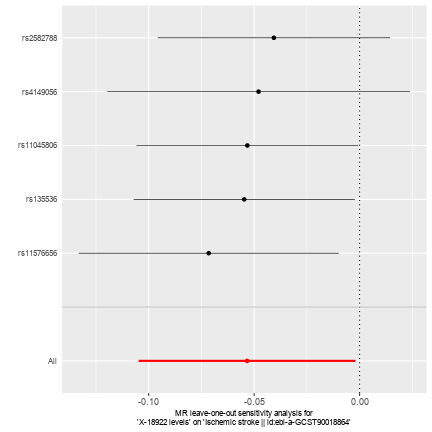

Supplement: Supplementary file 1 [file DataSheet1.ZIP › serum_metabolites/figure/X18922_levels_against_Ischemic_stroke__idebiaGCST90018864chunk8-1.png]

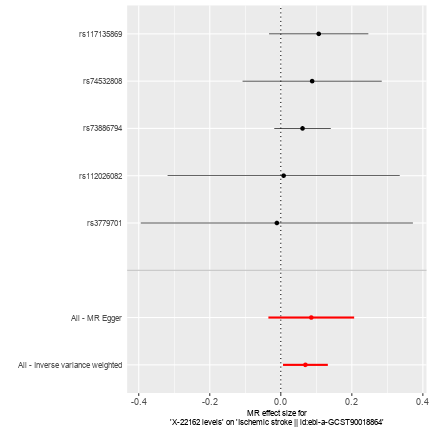

Supplement: Supplementary file 1 [file DataSheet1.ZIP › serum_metabolites/figure/X22162_levels_against_Ischemic_stroke__idebiaGCST90018864chunk5-1.png]

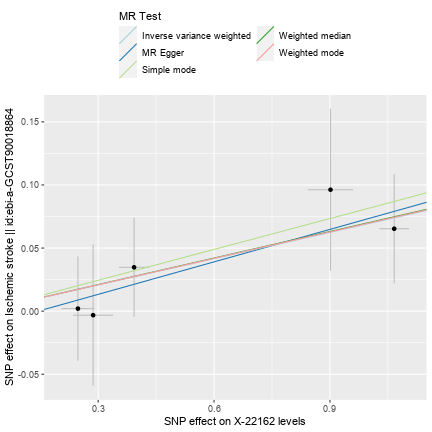

Supplement: Supplementary file 1 [file DataSheet1.ZIP › serum_metabolites/figure/X22162_levels_against_Ischemic_stroke__idebiaGCST90018864chunk6-1.png]

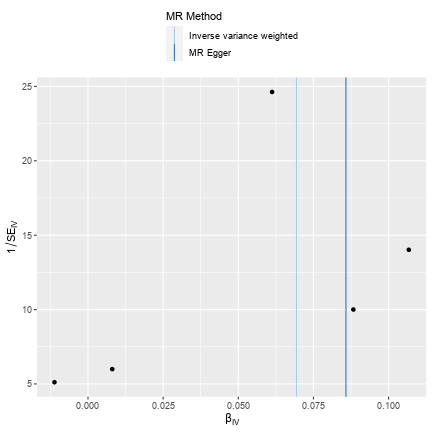

Supplement: Supplementary file 1 [file DataSheet1.ZIP › serum_metabolites/figure/X22162_levels_against_Ischemic_stroke__idebiaGCST90018864chunk7-1.png]

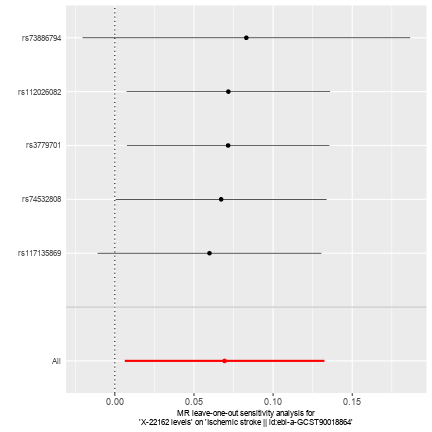

Supplement: Supplementary file 1 [file DataSheet1.ZIP › serum_metabolites/figure/X22162_levels_against_Ischemic_stroke__idebiaGCST90018864chunk8-1.png]

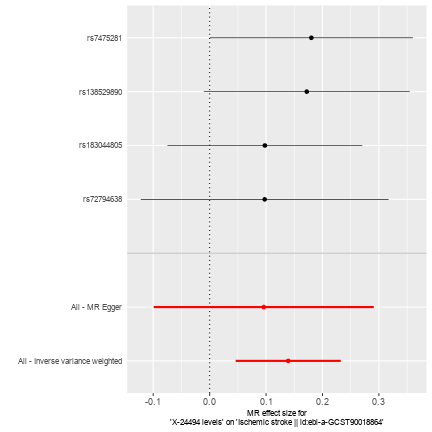

Supplement: Supplementary file 1 [file DataSheet1.ZIP › serum_metabolites/figure/X24494_levels_against_Ischemic_stroke__idebiaGCST90018864chunk5-1.png]

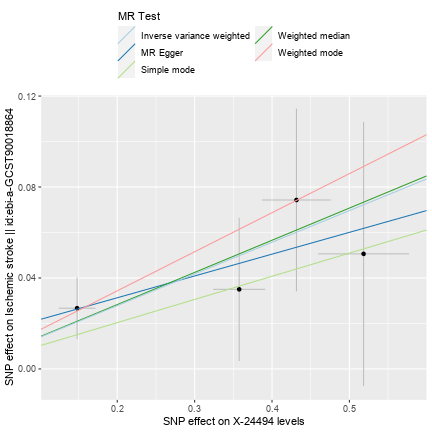

Supplement: Supplementary file 1 [file DataSheet1.ZIP › serum_metabolites/figure/X24494_levels_against_Ischemic_stroke__idebiaGCST90018864chunk6-1.png]

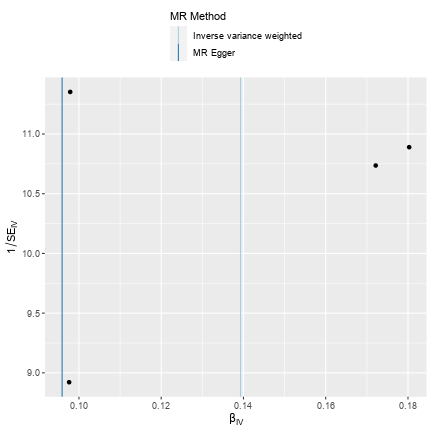

Supplement: Supplementary file 1 [file DataSheet1.ZIP › serum_metabolites/figure/X24494_levels_against_Ischemic_stroke__idebiaGCST90018864chunk7-1.png]

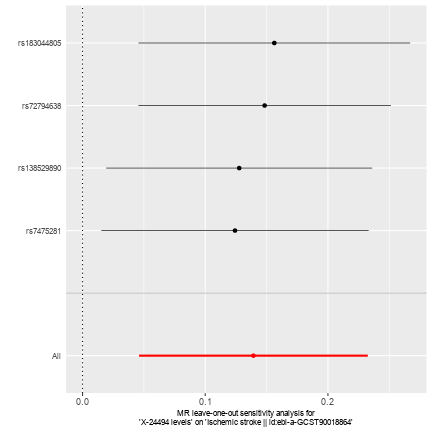

Supplement: Supplementary file 1 [file DataSheet1.ZIP › serum_metabolites/figure/X24494_levels_against_Ischemic_stroke__idebiaGCST90018864chunk8-1.png]
