# Supplementary material for: Causal relationship between human blood metabolites and risk of ischemic stroke: a Mendelian randomization study
Source: Front Genet. 2024 Jan 19;15:1333454. doi: 10.3389/fgene.2024.1333454 (PMC10834680; doi:10.3389/fgene.2024.1333454)
Supplement: Supplementary file 1 [file DataSheet1.ZIP › serum_metabolites/GCST90200706_funnel_plot.pdf]

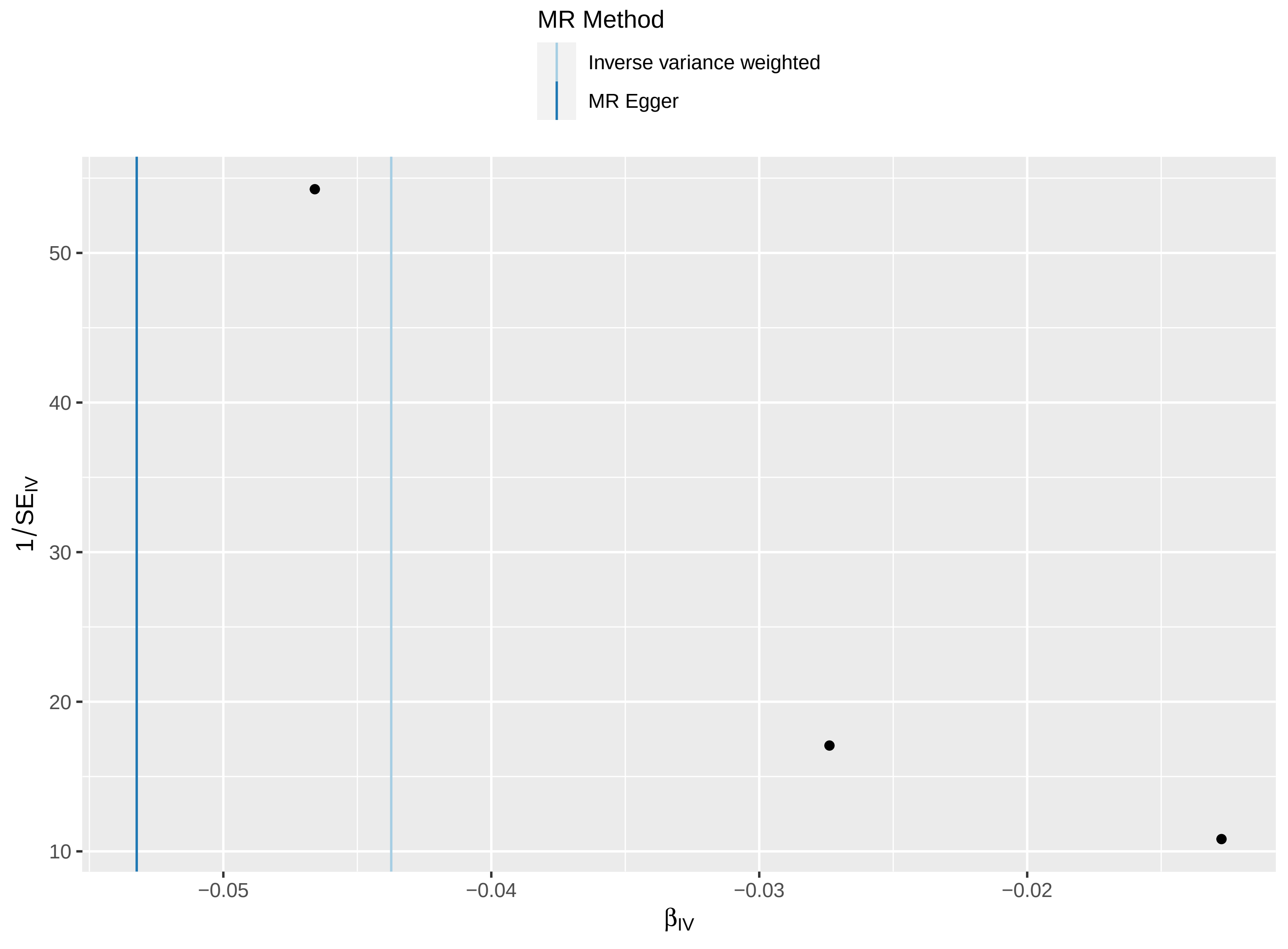
MR Method
Inverse variance weighted
MR Egger
1/SE_IV
50
40
30
20
10
−0.05
−0.04
−0.03
−0.02
β_IV
