# Supplementary material for: Causal relationship between human blood metabolites and risk of ischemic stroke: a Mendelian randomization study
Source: Front Genet. 2024 Jan 19;15:1333454. doi: 10.3389/fgene.2024.1333454 (PMC10834680; doi:10.3389/fgene.2024.1333454)
Supplement: Supplementary file 1 [file DataSheet1.ZIP › serum_metabolites/GCST90200726_forest_plot.pdf]

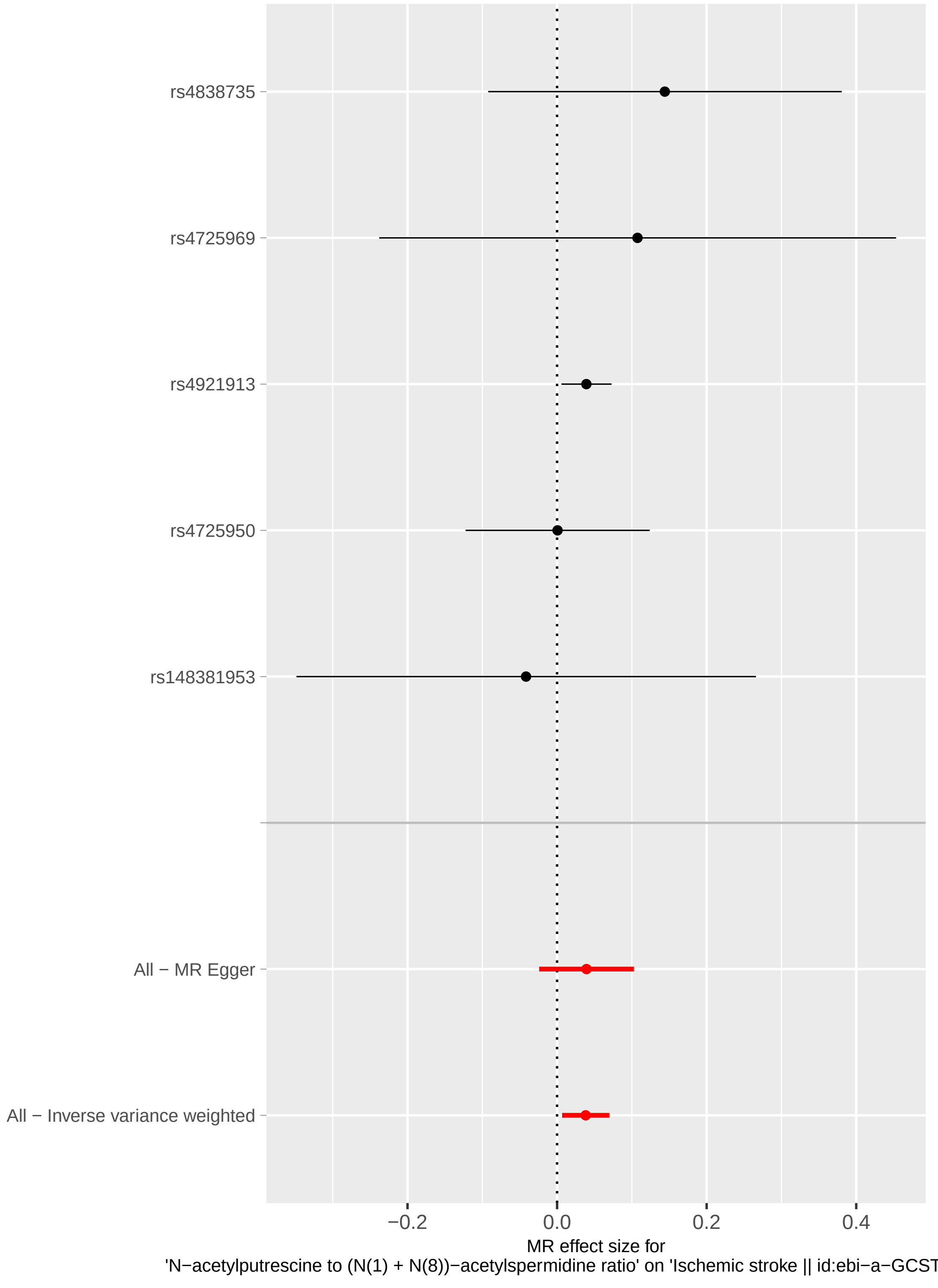
rs4838735
rs4725969
rs4921913
rs4725950
rs148381953
All − MR Egger
All − Inverse variance weighted
−0.2
0.0
0.2
0.4
MR effect size for
'N−acetylputrescine to (N(1) + N(8))−acetylspermidine ratio' on 'Ischemic stroke || id:ebi−a−GCST
