# Supplementary material for: Causal relationship between human blood metabolites and risk of ischemic stroke: a Mendelian randomization study
Source: Front Genet. 2024 Jan 19;15:1333454. doi: 10.3389/fgene.2024.1333454 (PMC10834680; doi:10.3389/fgene.2024.1333454)
Supplement: Supplementary file 1 [file DataSheet1.ZIP › serum_metabolites/GCST90200726_funnel_plot.pdf]

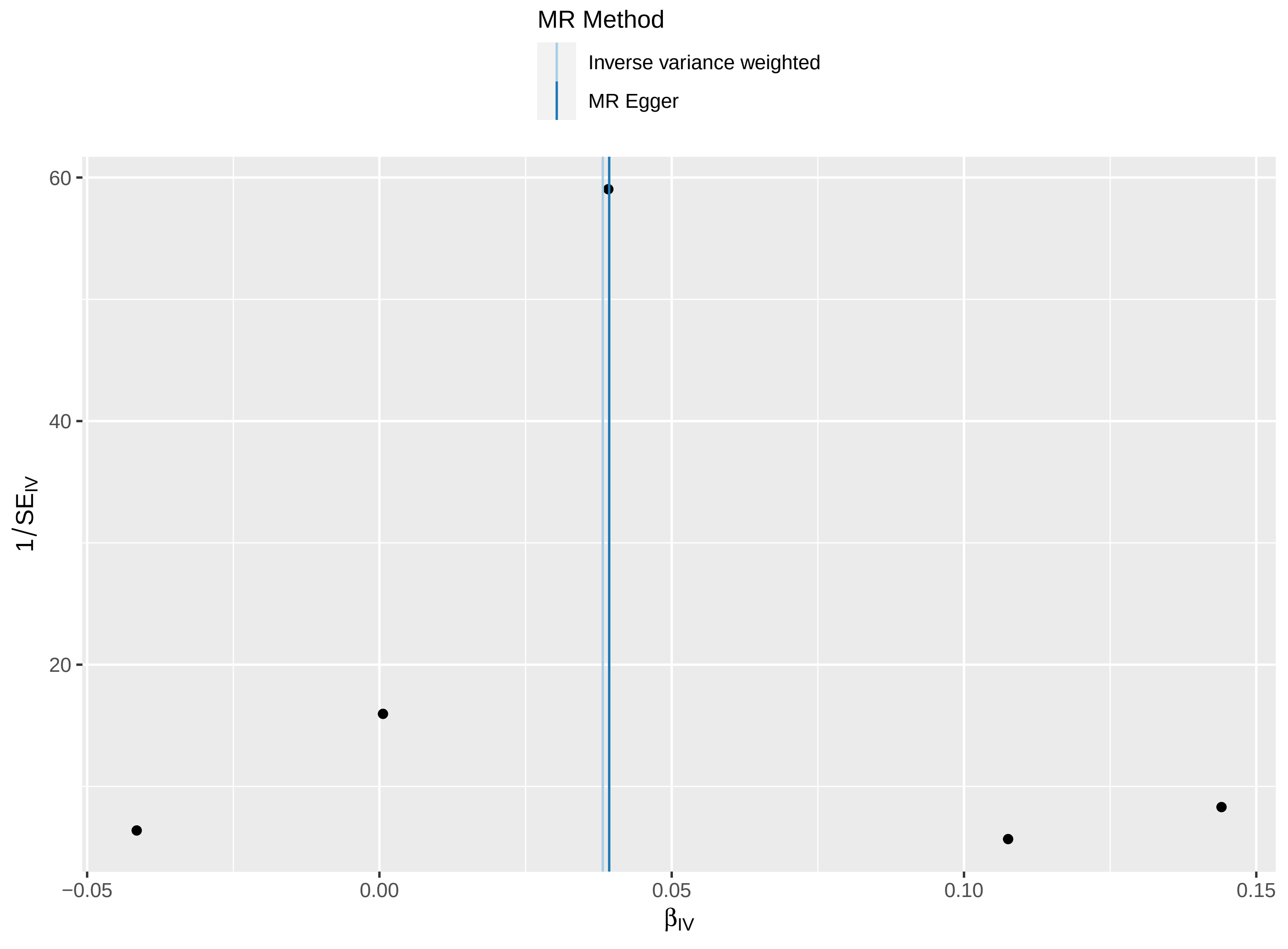

MR Method
Inverse variance weighted
MR Egger
60
40
20
1/SE_IV
−0.05
0.00
0.05
0.10
0.15
β_IV
