# Supplementary material for: Causal relationship between human blood metabolites and risk of ischemic stroke: a Mendelian randomization study
Source: Front Genet. 2024 Jan 19;15:1333454. doi: 10.3389/fgene.2024.1333454 (PMC10834680; doi:10.3389/fgene.2024.1333454)
Supplement: Supplementary file 1 [file DataSheet1.ZIP › serum_metabolites/GCST90200726_leaveOneOut_plot.pdf]

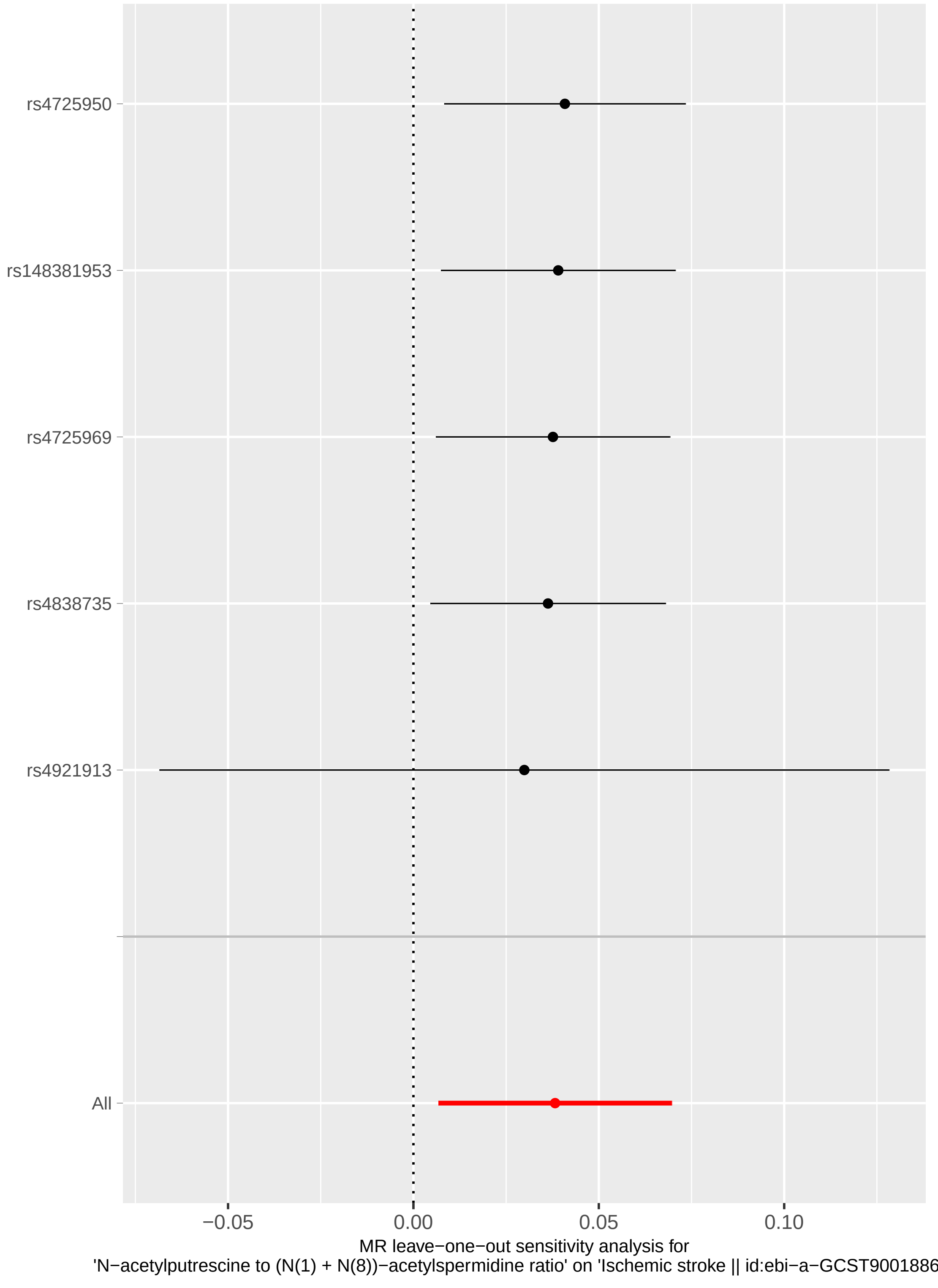

rs4725950
rs148381953
rs4725969
rs4838735
rs4921913
All
−0.05
0.00
0.05
0.10
MR leave−one−out sensitivity analysis for
'N−acetylputrescine to (N(1) + N(8))−acetylspermidine ratio' on 'Ischemic stroke || id:ebi−a−GCST9001886
