# Supplementary material for: Causal relationship between human blood metabolites and risk of ischemic stroke: a Mendelian randomization study
Source: Front Genet. 2024 Jan 19;15:1333454. doi: 10.3389/fgene.2024.1333454 (PMC10834680; doi:10.3389/fgene.2024.1333454)
Supplement: Supplementary file 1 [file DataSheet1.ZIP › serum_metabolites/GCST90200774_funnel_plot.pdf]

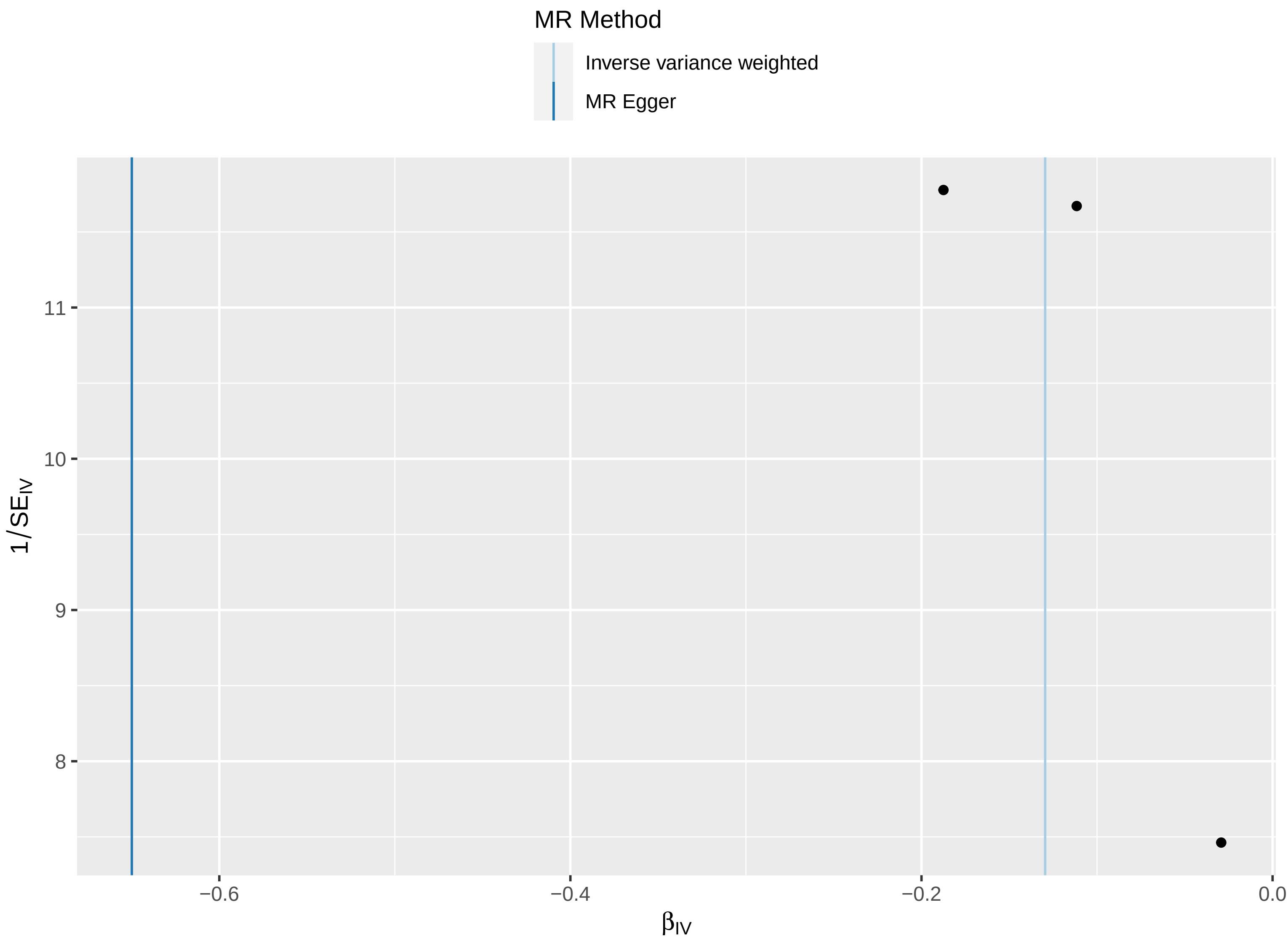
MR Method
Inverse variance weighted
MR Egger
11
10
9
8
1/SE_IV
−0.6
−0.4
−0.2
0.0
β_IV
