# Supplementary material for: Causal relationship between human blood metabolites and risk of ischemic stroke: a Mendelian randomization study
Source: Front Genet. 2024 Jan 19;15:1333454. doi: 10.3389/fgene.2024.1333454 (PMC10834680; doi:10.3389/fgene.2024.1333454)
Supplement: Supplementary file 1 [file DataSheet1.ZIP › serum_metabolites/GCST90201015_forest_plot.pdf]

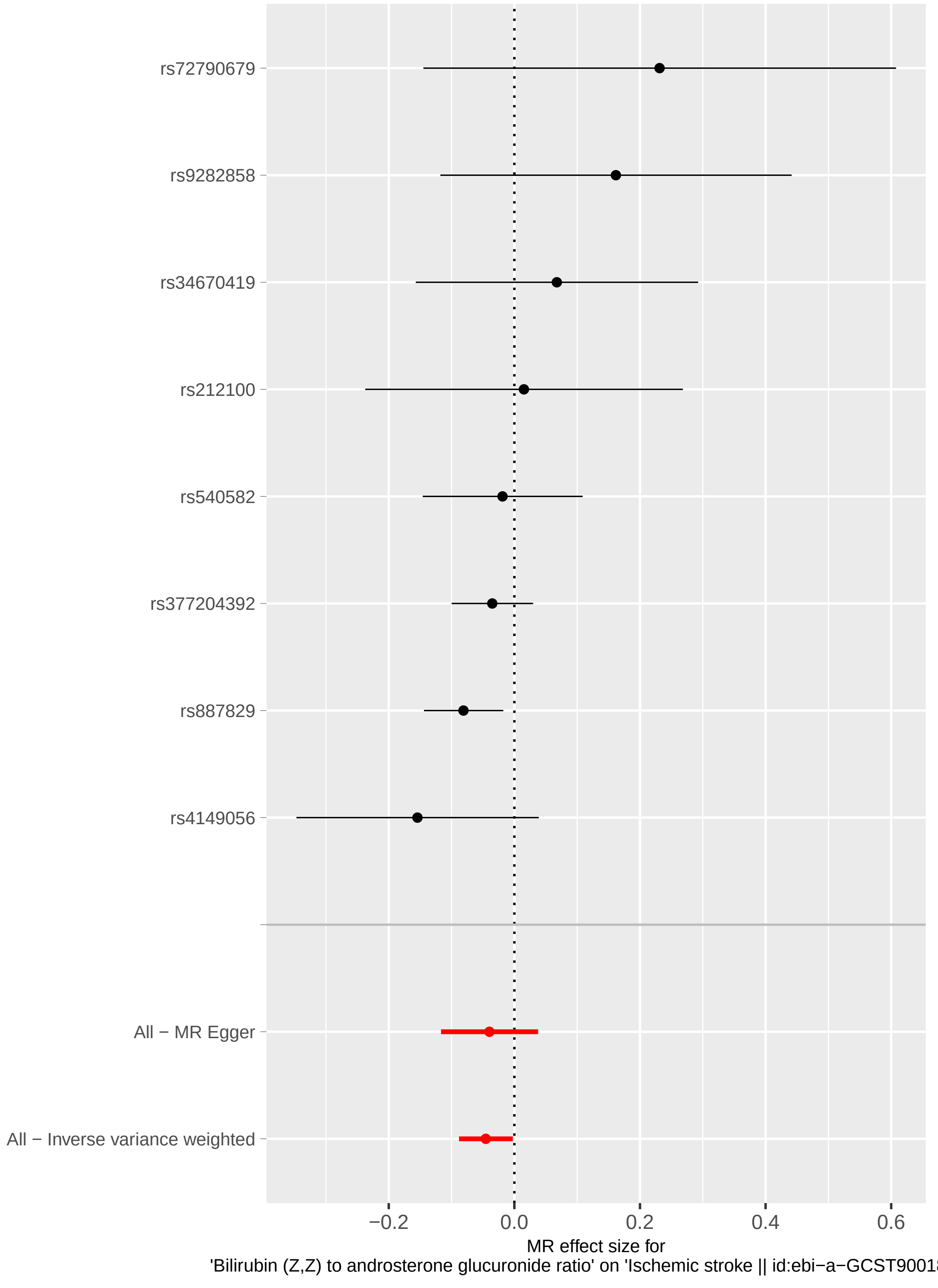
rs72790679
rs9282858
rs34670419
rs212100
rs540582
rs377204392
rs887829
rs4149056
All − MR Egger
All − Inverse variance weighted
−0.2
0.0
0.2
0.4
0.6
MR effect size for
'Bilirubin (Z,Z) to androsterone glucuronide ratio' on 'Ischemic stroke || id:ebi−a−GCST9001
