# Supplementary material for: Causal relationship between human blood metabolites and risk of ischemic stroke: a Mendelian randomization study
Source: Front Genet. 2024 Jan 19;15:1333454. doi: 10.3389/fgene.2024.1333454 (PMC10834680; doi:10.3389/fgene.2024.1333454)
Supplement: Supplementary file 1 [file DataSheet1.ZIP › serum_metabolites/GCST90201015_leaveOneOut_plot.pdf]

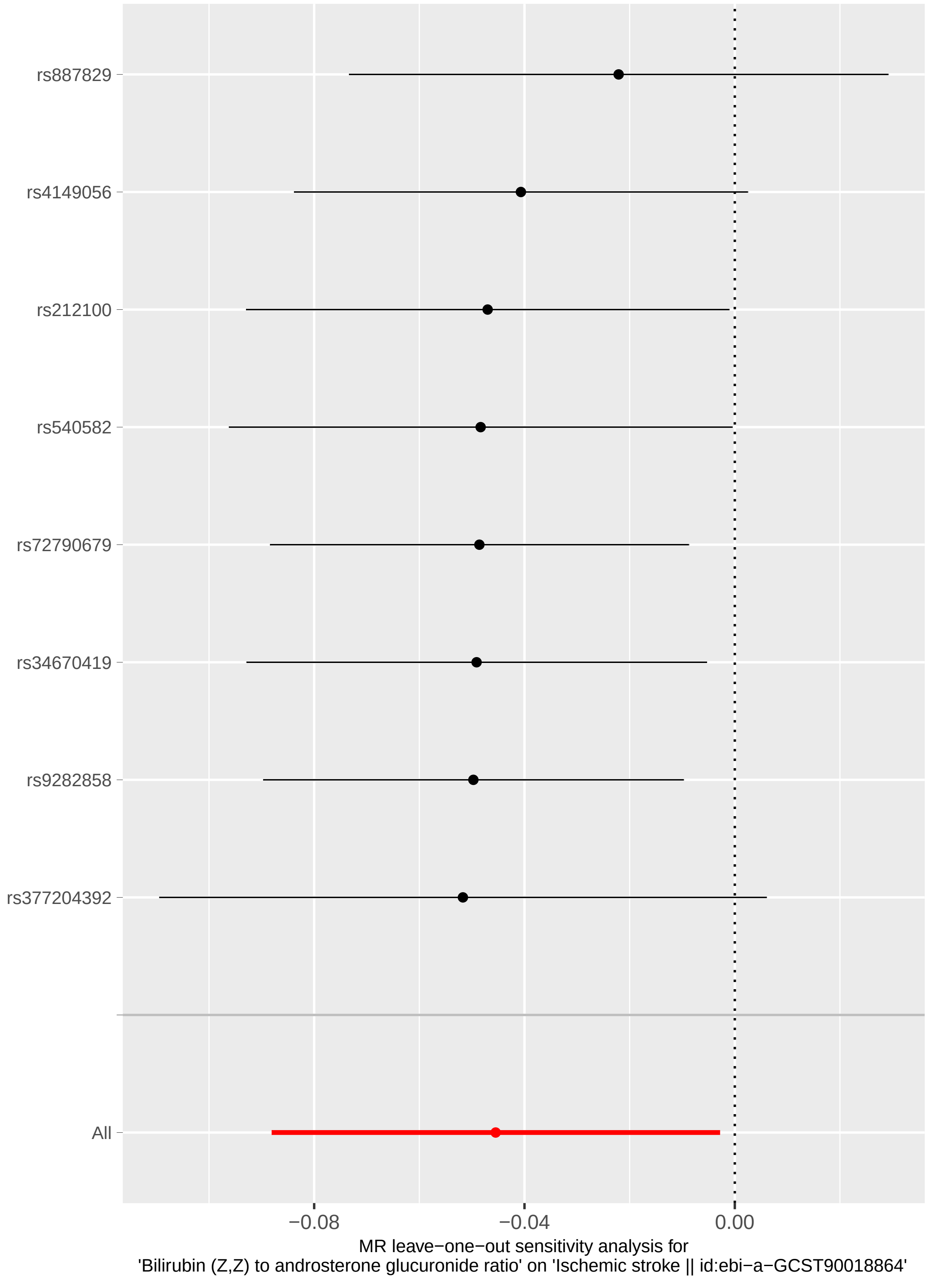
rs887829
rs4149056
rs212100
rs540582
rs72790679
rs34670419
rs9282858
rs377204392
All
−0.08
−0.04
0.00
MR leave−one−out sensitivity analysis for
'Bilirubin (Z,Z) to androsterone glucuronide ratio' on 'Ischemic stroke || id:ebi−a−GCST90018864'
