# Supplementary material for: Causal relationship between human blood metabolites and risk of ischemic stroke: a Mendelian randomization study
Source: Front Genet. 2024 Jan 19;15:1333454. doi: 10.3389/fgene.2024.1333454 (PMC10834680; doi:10.3389/fgene.2024.1333454)
Supplement: Supplementary file 1 [file DataSheet1.ZIP › serum_metabolites/GCST90201016_forest_plot.pdf]

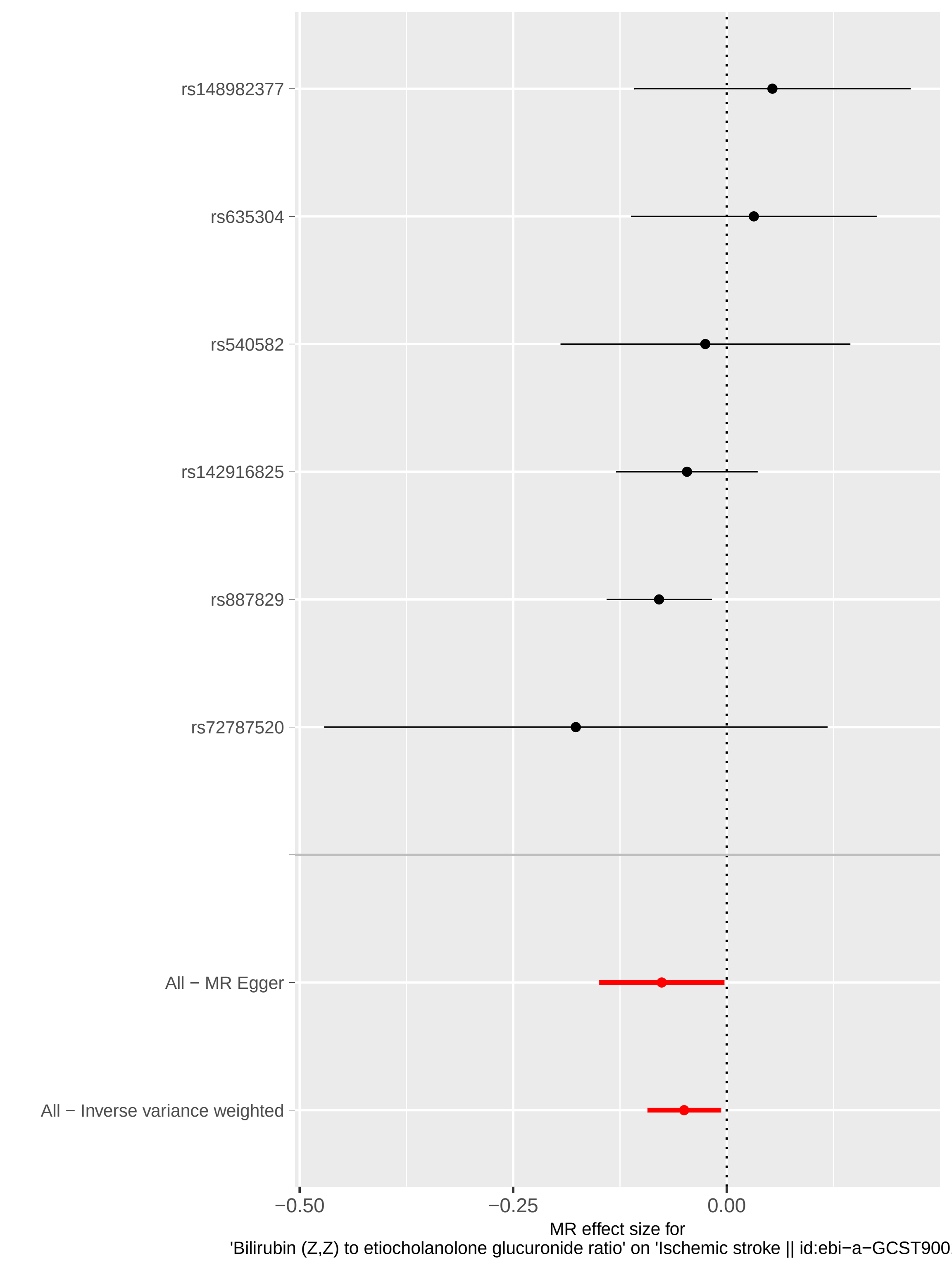
rs148982377
rs635304
rs540582
rs142916825
rs887829
rs72787520
All − MR Egger
All − Inverse variance weighted
−0.50
−0.25
0.00
MR effect size for
'Bilirubin (Z,Z) to etiocholanolone glucuronide ratio' on 'Ischemic stroke || id:ebi−a−GCST900
