# Supplementary material for: Causal relationship between human blood metabolites and risk of ischemic stroke: a Mendelian randomization study
Source: Front Genet. 2024 Jan 19;15:1333454. doi: 10.3389/fgene.2024.1333454 (PMC10834680; doi:10.3389/fgene.2024.1333454)

id.exposure	nsnp	OR (95% CI)	pval
GCST90199626	4	0.89 (0.83 to 0.97)	5.8e−03
GCST90199669	2	1.24 (1.08 to 1.44)	2.7e−03
GCST90199782	5	1.09 (1.01 to 1.17)	2.4e−02
GCST90199798	4	0.91 (0.85 to 0.98)	1.0e−02
GCST90199815	5	1.09 (1.02 to 1.16)	1.1e−02
GCST90199841	8	0.97 (0.94 to 1.00)	4.1e−02
GCST90199873	7	0.93 (0.87 to 0.99)	2.2e−02
GCST90199876	4	1.18 (1.01 to 1.39)	3.7e−02
GCST90200081	5	0.92 (0.84 to 1.00)	4.6e−02
GCST90200114	3	0.92 (0.86 to 0.99)	2.3e−02
GCST90200127	3	1.08 (1.01 to 1.16)	2.2e−02
GCST90200152	3	0.92 (0.86 to 0.98)	1.1e−02
GCST90200384	5	1.08 (1.01 to 1.17)	3.0e−02
GCST90200392	3	0.97 (0.94 to 1.00)	3.9e−02
GCST90200578	5	0.95 (0.90 to 1.00)	4.2e−02
GCST90200608	5	1.07 (1.01 to 1.14)	3.1e−02
GCST90200635	4	1.15 (1.05 to 1.26)	3.4e−03
GCST90200661	6	1.07 (1.01 to 1.15)	3.2e−02
GCST90200670	3	0.93 (0.87 to 0.99)	2.8e−02
GCST90200675	4	1.04 (1.00 to 1.08)	3.1e−02
GCST90200680	4	1.04 (1.02 to 1.07)	2.2e−03
GCST90200686	3	0.96 (0.93 to 0.99)	1.1e−02
GCST90200703	3	0.95 (0.92 to 0.99)	7.0e−03
GCST90200706	3	0.96 (0.93 to 0.99)	1.1e−02
GCST90200726	5	1.04 (1.01 to 1.07)	1.7e−02
GCST90200774	3	0.88 (0.79 to 0.98)	1.8e−02
GCST90201015	8	0.96 (0.92 to 1.00)	3.6e−02
GCST90201016	6	0.95 (0.91 to 0.99)	2.3e−02

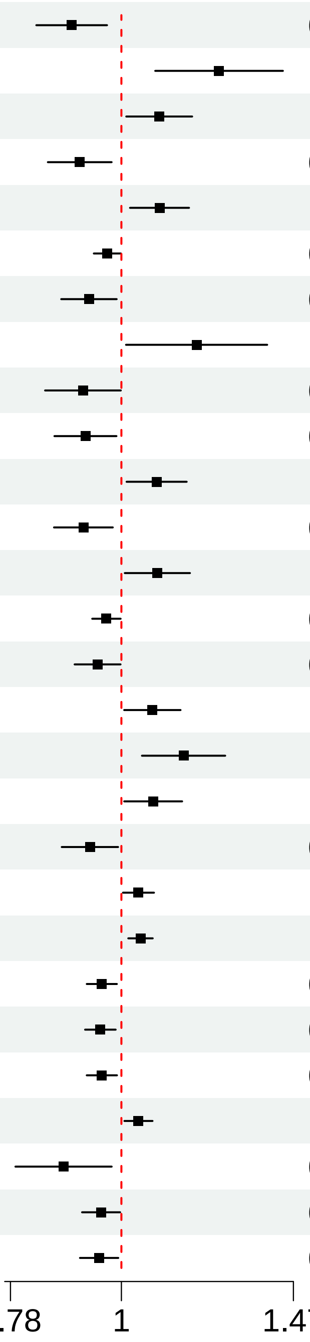

Supplement: Supplementary file 1 [file DataSheet1.ZIP › serum_metabolites/MR_forest.pdf]
