# Supplementary material for: Causal relationship between human blood metabolites and risk of ischemic stroke: a Mendelian randomization study
Source: Front Genet. 2024 Jan 19;15:1333454. doi: 10.3389/fgene.2024.1333454 (PMC10834680; doi:10.3389/fgene.2024.1333454)
Supplement: Supplementary file 1 [file DataSheet1.ZIP › serum_metabolites/TwoSampleMR.1oleoyl2docosahexaenoylGPC_181226_levels_against_Ischemic_stroke__idebiaGCST90018864.html]

Two sample MR report


# Two sample MR report

---

## 1-oleoyl-2-docosahexaenoyl-GPC (18:1/22:6) levels against Ischemic stroke || id:ebi-a-GCST90018864

Date: **29 October, 2023**

---

### Results from two sample MR:

| method | nsnp | b | se | pval |
| --- | --- | --- | --- | --- |
| MR Egger | 5 | -0.322 | 0.308 | 0.372 |
| Weighted median | 5 | -0.092 | 0.055 | 0.092 |
| Inverse variance weighted | 5 | -0.086 | 0.043 | 0.046 |
| Simple mode | 5 | -0.009 | 0.081 | 0.915 |
| Weighted mode | 5 | -0.146 | 0.080 | 0.143 |

---

### Heterogeneity tests

| method | Q | Q\_df | Q\_pval |
| --- | --- | --- | --- |
| MR Egger | 2.47 | 3 | 0.480 |
| Inverse variance weighted | 3.07 | 4 | 0.546 |

---

### Test for directional horizontal pleiotropy

| egger\_intercept | se | pval |
| --- | --- | --- |
| 0.026 | 0.034 | 0.495 |

---

### Test that the exposure is upstream of the outcome

| snp\_r2.exposure | snp\_r2.outcome | correct\_causal\_direction | steiger\_pval |
| --- | --- | --- | --- |
| 0 | 0 | TRUE | 0.35 |

Note - R^2^ values are approximate

---

### Forest plot of single SNP MR

---

### Comparison of results using different MR methods

---

### Funnel plot

---

### Leave-one-out sensitivity analysis
